# Supplementary material for: A scoping review of the impacts of COVID-19 physical distancing measures on vulnerable population groups
Source: Nat Commun. 2023 Feb 3;14:599. doi: 10.1038/s41467-023-36267-9 (PMC9897623; doi:10.1038/s41467-023-36267-9)
Supplement: Supplementary file 1 — Supplementary Information [file 41467_2023_36267_MOESM1_ESM.pdf]

Supplemental Information - A scoping review of the impacts of COVID-19 physical distancing measures on vulnerable population groups

Lili Li,[1] Araz Taeihagh[1,*] and Si Ying Tan[2]

[1] Policy Systems Group, Lee Kuan Yew School of Public Policy, National University of Singapore

[2] Alexandra Research Centre for Healthcare in The Virtual Environment (ARCHIVE), Department of Healthcare Redesign, Alexandra Hospital, National University Health System, Singapore

Content

Supplemental Information - A scoping review of the impacts of COVID-19 physical distancing measures on vulnerable population groups 1

Supplementary Table 1. List of studies included in the first round in 2021 3

Supplementary Table 2. List of studies added in 2022 26

Supplementary Table 3. Distribution of studies across countries and vulnerable populations 37

Supplementary Table 4. Summarized characteristics of included studies 38

Supplementary Table 5. Data for Fig. 2a global map 39

Supplementary Table 6. Data for Fig. 2b data distribution around Europe 40

Supplementary Table 7. Data for Fig. 2c data distribution around Asia 41

Supplementary Text 42

A1. Scope of physical distancing measures 42

A2. Classification of vulnerability 43

A3. Data and methods 45

A4. Results 46

1) Older people, vulnerability & physical distancing 46

2) Children/students, vulnerability & physical distancing 47

3) Low-income populations, vulnerability & physical distancing 50

4) Migrant workers, vulnerability & physical distancing 53

5) People in prison, vulnerability & physical distancing 54

6) People with disabilities, vulnerability & physical distancing 55

7) Sex workers, vulnerability & physical distancing 57

8) Victims of domestic violence, vulnerability & physical distancing 58

9) Refugees, vulnerability & physical distancing 58

* Corresponding author: Araz Taeihagh, Lee Kuan Yew School of Public Policy, National University of Singapore, 469B Bukit Timah Road, Li Ka Shing Building, Singapore 259771, spparaz@nus.edu.sg

10) Ethnic minority, vulnerability & physical distancing 59
11) People from sexual and gender minorities, vulnerability & physical distancing 60
12) Summary of results 61
References 63

Supplementary Table 1. List of studies included in the first round in 2021

No.	Title	Population of interest	First author	Year	Country	Study design/type	Data source	Brief description
1	Sex Workers Should Not Be Forgotten in Africa's COVID-19 Response	Sex workers	Adebisi, Y.A.	2020	Africa	Perspective piece	Secondary data	It advocates for an inclusive policy response amid the Covid-19 in Africa that take into account the sex workers' healthcare access and difficulties.
2	How the Coronavirus Lockdown Impacts the Impoverished in India	Low-income populations	Agoramoorthy, G.	2021	India	Perspective piece	Secondary data	The article presents the impact of the Covid-19 lockdown on the low income population in India.
3	COVID-19: US shelter-in-place orders and demographic characteristics linked to cases, mortality, and recovery rates	Low-income populations, particularly the homeless	Alderman, J.	2020	USA	Quantitative study (Multivariate regression analysis)	Secondary data	The article analyses how COVID-19 infections, mortality and recovery rates were related with the duration of states' shelter-in-place measures and demographic characteristics.
4	Refugees and covid-19: Achieving a comprehensive public health response	Refugees	Alemi, Q.	2020	Not specific	Editorial	Secondary data	This commentary advocates for thorough protective health measures to shield the refugees in US against COVID-19.
5	Effects of COVID-19 pandemic and lockdown on lifestyle and mental health of students: A retrospective study from Karachi, Pakistan	Children/Students	Ali, A.	2021	Pakistan	Retrospective cross-sectional study (Survey and regression analysis)	Primary survey data	The article examines changes in students' sleep patterns, time perception, and usage of digital media during the lockdown in Pakistan, and how those changes affected mental health of students.
6	How should policy responses to the COVID-19 pandemic differ in the developing world?	Low-income populations	Alon, T.	2020	Not specific	Model simulation	Secondary data	The article simulates how developing countries differ from the developed countries in terms of the effects of various COVID-19 policy measures.
7	Digital technologies as a panacea for social isolation and loneliness among older adults: An intervention model for flourishing and wellbeing visual technologies as a panacea for social isolation	Older people	Amundsen, D.	2021	Not specific	Literature review	Secondary data	This article calls for technology to be considered as part of the solution to alleviate social isolation among the older people.
8	Covid-19 Lockdowns, Income Distribution, and Food Security: An analysis for South Africa	Low-income populations	Arndt, C.	2020	South Africa	Single country case study (Input-output tables)	Secondary data	The article investigates the implications of the COVID-19 lockdown for income distribution and food security.

No.	Title	Population of interest	First author	Year	Country	Study design/type	Data source	Brief description
9	Association between statewide school closure and COVID-19 incidence and mortality in the US	Children/Students	Auger, K. A.	2020	USA	Quantitative study (Interrupted time series analysis)	Secondary data	It assesses how school closure and its timing affected reduction of COVID-19 incidence and mortality.
10	The Psychological Effects of COVID-19 Pandemic Related Lockdown in Children	Children/Students	Babu, T.A.	2020	India	Correspondence	Secondary data	It addresses the psychological effects of the lockdown in India among children.
11	Coronavirus Disease 2019, School Closures, and Children's Mental Health	Children/Students	Bahn, G. H.	2020	Not Specific	Literature review	Secondary data	The article reviews how existing studies measure mental health issues of Children due to the COVID-19 and related school closures.
12	Socially Distanced or Socially Connected? Well-being through ICT Usage among the Indian Elderly during COVID-19	Older people	Bakshi, T.	2021	India	Qualitative study (interview)	Primary interview data	This article weighs the pros and cons of introducing technological usage among the older adults during the COVID-19 situation.
13	A county-level study of the effects of state-mandated COVID-19 lockdowns on urban and rural restaurant visits using consumers' cell phone geo-location data	Low-income populations, particularly low-income rural areas	Banerjee, T.	2021	USA	Quantitative study (difference-in-difference method)	Secondary data	It evaluates the effects of mandatory COVID-19 lockdowns on rural and urban restaurant visits in states of the US.
14	The benefits and costs of social distancing in high- and low-income countries	Low-income populations	Barnett-Howell, Z.	2021	178 countries	Model simulation	Secondary data	The article simulates the benefits and costs of various social distancing policy scenarios in high and low-income country settings.
15	Suffering in silence: How COVID-19 school closures inhibit the reporting of child maltreatment	Victims of domestic violence (child maltreatment)	Baron, E. J.	2020	USA	Quantitative study (regression analysis and prediction)	Secondary data	The article illustrates that school closures during the COVID-19 may impede the reporting of child maltreatment.
16	COVID Social Distancing and the Poor: An Analysis of the Evidence for England	Low-income populations	Basu, P.	2021	UK	Quantitative study (Panel regression analysis)	Secondary data	The article assesses the behavioral response of the low income populations to virus threats and social distancing policy measures in England.
17	All things equal? Heterogeneity in policy effectiveness against COVID-19 spread in Chile	Low-income populations	Bennett, M.	2021	Chile	Quantitative study (Augmented Synthetic Control Method)	Secondary data	The paper examines the effectiveness of social distancing and testing policy measures on containing the COVID-19 spread and the heterogeneity of the effectiveness across populations at different income levels.

No.	Title	Population of interest	First author	Year	Country	Study design/type	Data source	Brief description
18	Anxiety level among students of different college and universities in India during lock down in connection to the COVID-19 pandemic	Children/Students	Biswas, S.	2021	India	Cross-sectional study (Survey and statistical analysis)	Primary survey data	The article examines the mental anxiety experienced by college and university studdents during the nationwide lockdown in India.
19	Economic and social consequences of human mobility restrictions under COVID-19	Low-income populations	Bonaccorsi, G.	2020	Italy	Quantitative study (Regression analysis)	Secondary data	The article studies the socioeconomic consequences of COVID-19 lockdown in Italy.
20	Alarming trends in US domestic violence during the COVID-19 pandemic	Victims of domestic violence	Boserup, B.	2020	USA	Letter	Secondary data	It shows the alarming increase in domestic violence in the US in the era of the COVID-19.
21	Using social contact data to predict and compare the impact of social distancing policies with implications for school re-opening	Children/Students	Brooks-Pollock, E.	2020	UK	Model simulation	Secondary data	The article simulates the impact of school reopening on the reproduction number taking into account other social distancing policy measures in the UK.
22	Effectiveness of isolation policies in schools: Evidence from a mathematical model of influenza and COVID-19	Children/Students	Burns, A.A.C.	2021	USA	Model simulation	Secondary data	The article simulates the effectiveness of social distancing policy measures in schools, including isolating the infected cases or symptom-based isolation as well as shortening school weeks.
23	Gendered effects of school closures during the COVID-19 pandemic	Children/Students	Burzynska, K.	2020	Not specific	Corresponden ce	Secondary data	It argues that girls suffer more from unintended consequences of school closures.
24	Sex workers are returning to work and require enhanced support in the face of COVID-19: Results from a longitudinal analysis of online sex work activity and a content analysis of safer sex work guidelines	Sex workers	Callander, D.	2020	Not specific	Letter	Secondary data	It shows sex workers' activity after a period of social distancing and analyses content of five sex work and COVID-19 guidelines distributed by community-led organisations.
25	Older Adults' Intention to Socially Isolate Once COVID-19 Stay-at-Home Orders Are Replaced With "Safer-at-Home" Public Health Advisories: A Survey of Respondents in Maryland	Older people	Callow, M.A.	2020	USA	Descriptive cross-sectional study (survey and regression analysis)	Primary survey data	This article explores the mindset and behaviours of the older people when COVID-19 guidelines are changed.
26	Unequal effects of the national lockdown on mental and social health in Italy	Children/Students	Caroppo, E.	2020	Italy	Observational study (survey and statistical analysis)	Primary survey data	The article presents that the effects of the nationwide lockdown in Italy on mental and social health were unequally distributed among different gender and age groups.

No.	Title	Population of interest	First author	Year	Country	Study design/type	Data source	Brief description
27	Mental Health and Holistic Care of Migrant Workers in Singapore during the COVID-19 pandemic	Migrant Workers	Chan, L. G.	2020	Singapore	Viewpoint piece	Secondary data	It discusses the mental health and other healthcare access among migrant workers in Singapore at the era of the COVID-19.
28	Differential Impact of Mitigation Policies and Socioeconomic Status on COVID-19 Prevalence and Social Distancing in the United States	Low-income populations	Chang, H.-Y.	2021	USA	Quantitative study (Comparative interrupted time-series analysis)	Secondary data	The article measured the differential effect of stay-at-home orders on COVID-19 infections and residents' mobility over communities with different socioeconomic status.
29	Debates around the Role of School Closures in the Coronavirus 2019 Pandemic-Reply	Children/Students	Cheng, S. O.	2021	Not specific	Letter	Secondary data	It presents that Taiwan slowed the COVID-19 transmission without closing schools, and Denmark and Norway saw continues declines in new infections after reopening schools.
30	Impact of covid-19 pandemic lockdown on mental and physical health of students: A cross-sectional study	Children/Students	Chitapure, T.	2020	India	Cross-sectional study (survey and statistical analysis)	Primary survey data	A survey was conducted to examine the impact of the COVID-19 lockdown in India on students' mental and physical health.
31	COVID-19 Social Distancing Measures and Loneliness Among Older Adults	Older people	Choi, E. Y.	2020	USA	Cross sectional study (Regression analysis)	Secondary data	This article investigates how social distancing guidelines may induce feelings of isolation among the older people.
32	COVID 19 pandemic: Mental health challenges of internal migrant workers of India	Migrant Workers	Choudhari, R.	2020	India	Qualitative study	Secondary data	The article illustrates how the COVID-19 negatively affected the mental health of internal migrant workers.
33	A scenario to safely ease the covid-19 lockdown while allowing economic recovery	Older people	Claverie, J.-M.	2020	France	Correspondence	Secondary data	It suggests that the lockdown be gradually released while allowing economic recovery.
34	A call for strengthened evidence on targeted, non-pharmaceutical interventions against COVID-19 for the protection of vulnerable individuals in sub-Saharan Africa	Older people	Colebunders, R.	2020	sub-Saharan Africa	Single country case study (descriptive research)	Secondary data	It advocates the existing social distancing measures to control the COVID-19 transmission to be complemented with targeted policy measures to safeguard the vulnerable individuals.
35	COVID-19 control in low-income settings and displaced populations: what can realistically be done?	Low-income populations	Dahab, M.	2020	Not specific	Comment	Secondary data	In contrast to draconian containment strategies on a wide scale, the article argues for economically and socially amenable policy measures to contain the COVID-19

No.	Title	Population of interest	First author	Year	Country	Study design/type	Data source	Brief description
								spread in low-income esettings and among displaced populations.
36	Psycho-social impact of social distancing and isolation due to the COVID-19 containment measures on patients with physical disabilities	People with disabilities	Dalise, S.	2021	Italy	Cross-sectional observational study (interview and statistical analysis)	Primary interview data	The article presents the impact of social distancing and isolation on social condition and mental health among patients with physical disabilities during the COVID-19
37	Elderly people and responses to COVID-19 in 27 Countries	Older people	Daoust, J.-F.	2020	27 countries (Australia, Brazil, Canada, Denmark, Finland, France, Germany, Hong Kong, Italy, Japan, Malaysia, South Korea, Mexico, Netherlands, Norway, Philippines, Saudi Arabia, Singapore, South Korea, Spain, Sweden, Taiwan, Thailand, United Arab Emir- ates (UAE), United Kingdom (UK), United States (USA) and Vietnam)	Quantitative study (Regression analysis)	Secondary survey data	This article utilised data from various countries to look at how the older adults are reacting to the COVID-19 situation.
38	How Are School Districts Addressing Student Social-Emotional Needs during the	Children/Students	DeArmond, M.	2021	USA	Research brief (content analysis)	Secondary data	The article discusses whether and how remote learning and school reopening guidelines of different school districts

No.	Title	Population of interest	First author	Year	Country	Study design/type	Data source	Brief description
	Pandemic? Center on Reinventing Public Education							addressed social-emotional needs of students.
39	Understanding patterns of adherence to COVID-19 mitigation measures: a qualitative interview study	Low-income populations; ethnic minority	Denford, S.	NA	UK	Qualitative study (Interview)	Primary interview data	The article presents the pattens of low-income and BAME communities' compliance with social distancing.
40	COVID-19 and School Closures	Children/Students	Donohue, J.M.	2020	USA	Editorial/comment	Secondary data	It comments on Auger et al (2020) and addresses negative consequences of school closures.
41	COVIDApp as an Innovative Strategy for the Management and Follow-Up of COVID-19 Cases in Long-Term Care Facilities in Catalonia: Implementation Study	Older people	Echeverría, P.	2020	Spain	Single case study (Statistical analysis)	Secondary data	This article introduces and explores the effectiveness of utilising a digital platform to lower the number of COVID-19 cases within places that houses many older adults.
42	School Closure During the Coronavirus Disease 2019 (COVID-19) Pandemic: An Effective Intervention at the Global Level?	Children/Students	Esposito, S.	2020	Global	Opinion/view point piece	Secondary data	It discusses the effectiveness of school closures in response to COVID-19 across countries.
43	COVID-19 and the re-opening of schools: A policy maker's dilemma	Children/Students	Fantini, M.P.	2020	Not specific	Letter	Secondary data	It discusses strategies for a safe reopening of schools for children aged 2-10 years old.
44	An International Review of Plans and Actions for School Reopening	Students, including students from low-income families	Fitzpatrick, R.	2020	Global	Report	Secondary data	The report summarises impacts of the school closure and the contexts and plans for school reopening.
45	From schoolification of children to schoolification of parents? -educational policies in COVID times	Children/Students	Formosinho, J.	2021	Portugal	Literature review	Secondary data	The article assesses the impact of the COVID-19 pandemic on early years education (e.g., kindergarden).
46	Early impact of social distancing in response to COVID-19 on hospitalizations for acute bronchiolitis in infants in Brazil	Children/Students	Friedrich, F.	2020	Brazil	Quantitative study (Statistical analysis)	Secondary data	The article evaluates the association between social distancing and hospital admissions for acute bronchiolitis in infants.
47	COVID-19 pandemic and social distancing in prisons	People in prison	Fróis, C.	2020	Portugal	Comment	Secondary data	It comments on Portuguese government's policy decision to release about 10% of people in prison.

No.	Title	Population of interest	First author	Year	Country	Study design/type	Data source	Brief description
48	Socioeconomic Disparities in Social Distancing during the COVID-19 Pandemic in the United States: Observational Study	Low-income populations	Garnier, R.	2021	USA	Observational study (using regression analysis)	Secondary data	The article investigates the relationship between mobility patterns and socioeconomic characteristics amid the COVID-19 pandemic in the US.
49	Informing children citizens efficiently to better engage them in the fight against COVID-19 pandemic	Children/Students	Ghia, J.-E.	2020	Not specific	Viewpoint piece	Secondary data	It advocates that reliable COVID-19 related information should be communicated to children to engage them in the fight against the pandemic.
50	Access to healthcare in a time of COVID-19: Sex workers in crisis in Nairobi, Kenya	Sex workers	Gichuna, S.	2020	Kenya	Qualitative study (interview)	Primary interview data	The article investigates the effects of COVID-19 and related social distancing restrictions on access to healthcare for sex workers in Nairobi.
51	Modelling interventions to control COVID-19 outbreaks in a refugee camp	Refugees	Gilman, R. T.	2020	Greece	Model simulation	Secondary data	This article simulates possible solutions and assess the possibility of it mitigating the COVID-19 infection rate within a refugee camp.
52	"We Need to Do Better by Kids": Changing Routines in U.S. Schools in Response to COVID-19 School Closures	Students, including students from ethnic minority backgrounds	Grooms, A. A.	2021	USA	Exploratory case study (Interview)	Primary interview data	The article examines how principals of K-12 schools made decisions responding to school closures to support remote learning, especially for students from racially and socioeconomically minoritized backgrounds.
53	COVID-19 lockdown and penalty of joblessness on income and remittances: A study of inter-state migrant labourers from Assam, India	Migrant workers, including the older migrant workers	Guha, P	2020	India	Single case study (Regression analysis)	Primary survey data	The article analyses how the lockdown in India negatively affected the employment condition, income and remittances among inter-state migrant workers.
54	Immediate impact of stay-at-home orders to control COVID-19 transmission on socioeconomic conditions, food insecurity, mental health, and intimate partner violence in Bangladeshi women and their families: an interrupted time series	Victims of domestic violence	Hamadani, J.D.	2020	Bangladesh	Quantitative study (Interrupted time series analysis)	Primary interview data	The article examines the impact of stay-at-home orders on Bangladeshi women and their families in terms of intimate partner violence, mental health, food insecurity, and other socioeconomic conditions.
55	Disparities in COVID-19 mortality by county racial composition and the role of spring social distancing measures	Ethnic minority	Hamman, M.K.	2021	USA	Quantitative study (Nonlinear regression decomposition and event study analysis)	Secondary data	The article analyses mortality rates across counties in the US with various Black populatioon shares, and the difference in the impact of social distancing measures.

No.	Title	Population of interest	First author	Year	Country	Study design/type	Data source	Brief description
56	Social Distancing and Incarceration: Policy and Management Strategies to Reduce COVID-19 Transmission and Promote Health Equity Through Decarceration	People in prison	Henry, B. F.	2020	Global	Perspective piece	Secondary data	It discusses the feasibility of decarceration to promote health equity and contain the epidemic while maintaining public safety.
57	Associations Between Physical Distancing and Mental Health, Sexual Health and Technology Use Among Gay, Bisexual and Other Men Who Have Sex with Men During the COVID-19 Pandemic	People from sexual and gender minorities	Holloway, I. W.	2021	Not specific	Cross-sectional study (survey and regression analysis)	Primary survey data	Based on an online survey, the article analyses the relation between physical distancing and the GBMSM (gay, bisexual, and other men who have sex with men) population's mental and sexual health and technology usage.
58	COVID-19 and schooling: evaluation, assessment, and accountability in times of crises-reacting quickly to explore key issues for policy, practice and research with the school barometer	Children/Students	Huber, S. G.	2020	Germany, Austria, Switzerland	Explorative study using survey and statistical analysis	Primary survey data	It presents and discusses a survey, the School Barometer, that was conducted in Germany, Austria and Switzerland to assess and evaluate the school situation at the initial stage of the school lockdown caused by COVID-19.
59	covid-19 lock-down socio-economic challenges faced by households in rural areas: a perspective from Vhembe District, South Africa	Low-income populations, particularly people in low-income rural areas	Iwara, I. O.	2020	South Africa	Single district case study (interview and qualitative analysis)	Primary interview data	Socio-economic challenges that rural households faced in South Africa during the nationwide lockdown were identified.
60	Was school closure effective in mitigating coronavirus disease 2019 (COVID-19)? Time series analysis using Bayesian inference	Children/Students	Iwata, K.	2020	Japan	Quantitative study (Time series analysis)	Secondary data	Effectiveness of school closues on curbing the COVID-19 transmission was analysed in Japan context.
61	Covid-19 lockdown and physical distancing policies are elitist: towards an indigenous (Afro-centred) approach to containing the pandemic in sub-urban slums in Nigeria	Low-income populations	Iwuoha, V. C.	2020	Nigeria	Qualitative study (Fieldwork and interviews)	Primary observational and interview data	The article examines the impact of COVID-19 lockdown and physical distancing on the extremely destitute and marginalised populations and argues for an indigenous approach to manage the COVID-19 pandemic in Nigeria.
62	Protecting sex workers in Thailand during the COVID-19 pandemic: opportunities to build back better	Sex workers	Janyam, S.	2020	Thailand	Perspective piece	Primary survey data	The article shows challenges that sex workers face amid the COVID-19 in Thailand and suggests the government to consider decriminalisation of sex workers and involvement of community-led organisations to protect sex workers.

No.	Title	Population of interest	First author	Year	Country	Study design/type	Data source	Brief description
63	Neighbourhood income and physical distancing during the COVID-19 pandemic in the United States	Low-income populations	Jay, J.	2020	USA	Quantitative study (Different-in-differences method)	Secondary data	The article analyses the relationship between income levels and adherence to physical distancing in communities of the US.
64	Modelling the impact of rapid tests, tracing and distancing in lower-income countries suggest optimal policies varies with rural-urban settings	Low-income populations	Jiang, X.	2021	Kenya, South Africa, Nigeria	Model simulation	Secondary data	The article simulates the differential impact of testing, trading and social distancing between rural and urban communities in low-and middle-income countries.
65	Exploring the mental health experiences of young trans and gender diverse people during the Covid-19 pandemic	People from sexual and gender minorities	Jones, B.A.	2021	UK	Mixed methods (survey with qualitative and quantitative questions)	Primary survey data	The article explores the impact of COVID-19 on the mental health of young trans and gender diverse people.
66	Assessing the nationwide impact of COVID-19 mitigation policies on the transmission rate of SARS-CoV-2 in Brazil	Low-income populations	Jorge, D. C. P.	2020	Brazil	Model simulation	Secondary data	The article evaluates the nationwide impact of governmental interventions to mitigate the COVID-19 transmission in Brazil.
67	Adherence to Public Health Measures Mitigates the Risk of COVID-19 Infection in Older Adults: A Community-Based Study	Older people	Juhn, Y. J.	2021	USA	Cross-sectional study (Statistical analysis)	Primary data	It highlights the importance of following public health guidelines to lower the risk of being infected with COVID-19 among the older people.
68	Coronavirus infections and deaths by poverty status: The effects of social distancing	Low-income populations	Jung, J.	2021	USA	Quantitative study (Regression analysis)	Secondary data	The article studies the patterns of COVID-19 infections and death rates across counties at different poverty and income levels in the US.
69	A crisis within the crisis: The mental health situation of refugees in the world during the 2019 coronavirus (2019-nCoV) outbreak	Refugees	Júnior, J.G.	2020	Not specific	Letter	Secondary data	This article explores the different elements that may lead to a deterioration of the mental well-being among refugees.
70	Investigating the E-Learning Challenges Faced by Students during Covid-19 in Namibia	Students, including students from low-income background	Kaisara, G.	2021	Namibia	Qualitative study (Thematic content analysis)	Primary survey data	The article reports the challenges that undergraduate students in Namibia faced to pursue e-learning during the COVID-19 lockdown.
71	"PHONE-CONNECT" - Prescribing Phones to Address Health Equity Needs in the COVID-19 Era	Low-income populations, particularly people in poverty or homeless	Kazevman, G.	2021	Canada	Single case study (Descriptive research)	Primary interview data	The article describes the PHONE-CONNECT program which enhance phone access to address health equity needs during the COVID-19 pandemic.

No.	Title	Population of interest	First author	Year	Country	Study design/type	Data source	Brief description
72	Evaluation of a novel community-based COVID-19 'Test-to-Care model' for low-income populations	Low-income populations	Kerkhoff, A. D.	2020	USA	Epidemiologic surveillance study (Demonstration project and interviews and survey among participants)	Primary interview and survey data	The article evaluates a "Test-to-care" programme which supports low-income populations diagnosed with COVID-19.
73	An adaptive governance and health system response for the COVID-19 emergency	Low-income people	Khan, M.	2021	Bangladesh	Letter	Secondary data	It advocates that an adaptive response strategy that can iteratively select the optimal set of health response measures and the network of organisations should be taken to respond to the COVID-19 crisis.
74	Language in a Time of COVID-19: Literacy Bias Ethnic Minorities Face During COVID-19 from Online Information in the UK	Ethnic minority	Khan, S.	2020	UK	Cross-sectional study (statistical analysis)	Secondary data	The article examines availability and readability of translated online COVID-19 information to the British ethnic minorities.
75	Recommendation for response to the COVID-19 pandemic: Korean context of distancing in daily life, considering vulnerable population	General vulnerable	Kim, J. Y.	2020	South Korea	Comment	Secondary data	It presents the COVID-19 policy response in Korea, i.e., "distancing in daily life".
76	Early school closures can reduce the first-wave of the COVID-19 pandemic development	Children/Students	Klimek-Tulwin, M.	2020	Argentina, Brazil, Belgium, Czechia, Finland, France, Germany, Italy, Norway, Spain, UK, Poland, Romania, Estonia, Hungary, Latvia, Lithuania, Japan	Quantitative study (Statistical analysis)	Secondary data	The article evaluates the effectiveness of school closures on containing the COVID-19 pandemic.
77	The role of mask mandates, stay at home orders and school closure in curbing the COVID-19 pandemic prior to vaccination	Children/Students	Krishnamachari, B.	2021	USA	Quantitative study (Regression analysis)	Secondary data	It assesses the impact of school closures, mask mandates, and stay-at-home orders on curbing the cumulative infection rates of COVID-19.

No.	Title	Population of interest	First author	Year	Country	Study design/type	Data source	Brief description
78	Measures implemented in the school setting to contain the COVID-19 pandemic: a rapid scoping review	Children/Students	Krishnaratne, S.	2020	Global	Literature review	Secondary data	It reviews measures implemented in school settings when schools are open, and how existing studies evaluate their impacts.
79	Migrant Workers and Human Rights: A Critical Study on India's COVID-19 Lockdown Policy	Migrant workers	Kumar, S.	2021	India	Single case study (Descriptive research)	Secondary data	The article argues that the nationwide lockdown in India caused various difficulties for the internal migrant workers.
80	Socio-economic impact of first 21 days nationwide lockdown-1 on the spread of SARS-COV-2 in India in relation to health	Migrant workers; students; low-income populations	Kumar, S.	2020	India	Literature review	Secondary data	It mainly shows the socio-economic implications of India's lockdown in the first 21 days.
81	COVID-19 response strategies: Considering inequalities between and within countries	Low-income populations	Lau, L.L.	2020	Philippines	Comment	Secondary data	The article argues that COVID-19 control measures need to be context specific and account for both between- country and within - country inequalities.
82	The psychological consequences of COVID-19 lockdowns	Ethnic minority	Le, K.	2021	USA	Quantitative study (survey and difference-in-differences analysis)	Primary survey data	The article shows adverse psychological impact of COVID-19 lockdowns.
83	Modelling the impact of school reopening on SARS-CoV-2 transmission using contact structure data from Shanghai	Children/Students	Lee, B.	2020	China	Model simulation	Secondary data	COVID-19 transmission under various policy scenarios of school reopening in Shanghai was simulated.
84	Facing COVID-19 and Refugee Camps on the U.S. Border	Refugees	Leiner, A.	2020	USA	Letter	Secondary data	This letter remarks about the situation with the refugees living on the US border.
85	Cross-sectional study of self-concept and gender in relation to physical activity and martial arts in Spanish adolescents during the COVID-19 lockdown	Children/Students	Lindell-Postigo, D.	2020	Spain	Cross-sectional study (Survey and statistical analysis)	Primary survey data	The article examines the association between the Spanish lockdown amid the COVID-19 and adolescents' self-concept and physical activity.
86	Sweden's Response to the COVID-19 Outbreak	People in prison	Lindström, M.	2020	Sweden	Single country case study	Secondary data	It discusses Sweden's policy measures taken to reduce the COVID-19 transmission.

No.	Title	Population of interest	First author	Year	Country	Study design/type	Data source	Brief description
87	Containing COVID-19 in rural and remote areas: Experiences from China	Low-income populations, particularly people in low-income rural/remote areas	Liu, X.	2020	China	Perspective piece	Primary survey data	The article describes practices and measures for controlling the COVID-19 spread in Chinese rural and remote areas.
88	Reopening Schools during the COVID-19 Pandemic: Overview and Rapid Systematic Review of Guidelines and Recommendations on Preventive Measures and the Management of Cases	Children/Students	Lo Moro, G.	2020	"France, Ireland, Italy, Luxembourg, Malta, Portugal, Spain, the United Kingdom, and San Marino"	Rapid systematic review	Secondary data	The systematic review examines the social distancing and quarantine measures planned or adopted across countries to manage the COVID-19 pandemic in school settings when schools reopen.
89	Socio-Educational Policies and Covid-19 - A Case Study on Finland and Sweden in the Spring 2020	Children/Students	Loima, J.	2020	Finland and Sweden	Qualitative case study/Hermeneutic phenomenology research method	Secondary data	It analyses socio-educational policy measures and argumentation in Finland and Sweden in March-May 2020.
90	Are stay-at-home orders more difficult to follow for low-income groups?	Low-income populations	Lou, J.	2020	USA	Quantitative study (Difference-in-difference-in-differences analysis)	Secondary data	The article presents different levels of adherence to stay-at-home orders among different income-level population groups.
91	Knowledge, Beliefs/Attitudes, and Practices of Rural Residents in the Prevention and Control of COVID-19: An Online Questionnaire Survey	Low-income populations, particularly people in low-income rural/remote mountain areas	Ma, L.	2020	China	Descriptive research (survey and statistical analysis)	Primary survey data	The article examines Chinese rural residents' knowledge, attitudes/beliefs and behavioral responses to prevent and control the spread of COVID-19.
92	Sexual health among Kenyan male sex workers in a time of COVID-19	Sex workers	Macharia, P.	2021	Kenya	Single case study (qualitative)	Primary data (informal interviews)	The article examines the effects of COVID-19 and the resultant lockdown on the access to sexual health services for marginalised male sex workers.
93	Household response to an extreme shock: Evidence on the immediate impact of the Covid-19 lockdown on economic outcomes and well-being in rural Uganda	Low-income populations, particularly people in low-income rural areas	Mahmud, M.	2021	Uganda	Mixed methods (interview and regression analysis)	Primary interview data	The article evaluates the impact of the COVID-19 lockdown in Uganda on the economic and well-being aspects of rural households.

No.	Title	Population of interest	First author	Year	Country	Study design/type	Data source	Brief description
94	Reopening in the Shadow of COVID-19: Beginning the First Full Coronavirus School Year	Children/Students	Malkus, N.	2020	USA	Report (Survey method)	Secondary data	The article describes how school districts reopened schools in fall 2020 in the US, and examines different models of operation and instruction across districts.
95	A review of India's response to COVID-19 outbreak and impact of lockdown on Indian economy	Low-income populations	Mandal, K. K.	2020	India	Single country case study (qualitative)	Secondary data	The article reviews the policy measures taken by India to combat the COVID-19 and analyses the impact of lockdown on the economy in India.
96	COVID-19 Lockdown Anxieties: Is student a vulnerable group?	Children/Students	Manjareeka, M.	2020	India	Prospective observational study (Survey)	Primary survey data	The article assesses students' anxiety issues during the COVID-19 lockdown compared to what they experienced prior to their regular exams.
97	"We've got through hard times before: acute mental distress and coping among disadvantaged groups during COVID-19 lockdown in North India - a qualitative study"	Low-income populations (including those living in slums), and those with psychosocial disabilities	Mathias, K.	2020	India	Qualitative study (Interview)	Primary interview data	The article examines the relationship between COVID-19 and acute mental distress as well as approaches that helped disadvantaged groups cope through the lockdown in India.
98	Urgent actions and policies needed to address COVID-19 among UK ethnic minorities	Ethnic minority	Mathur, R.	2020	UK	Comment	Secondary data	It shows concerns of the disproportionate impact of COVID-19 among the UK ethnic minorities and recommends the actions and policies needed.
99	COVID-19 and U.S.-based refugee populations: Commentary	Refugees	Mattar, S.	2020	USA	Case Study	Secondary data	This commentary encapsulates the key measures employed to protect the refugees in US.
100	Analysing the views of people in custody about the management of the COVID-19 pandemic in the Scottish Prison Estate	People in prison	Maycock, M.	2021	UK	Qualitative study (survey through letter writing combined with thematic analysis)	Primary survey data	The article assesses the COVID-19 policy response in the Scottish Prison Estate through analysing the views of people in custody.
101	COVID-19 outbreak, social distancing and mass testing in Kenya-insights from a mathematical model	Low-income populations, particularly people living in informal settlements	Mbogo, R.W.	2021	Kenya, South Africa	Model simulation	Secondary data	The article simulates the temporal dynamics of COVID-19 transmission, and which population to target for implementing mass testing.
102	Evaluating the Effect of COVID-19 Pandemic Lockdown on Long-Term Care Residents' Mental Health: A	Older people	McArthur, C.	2021	Canada	Quantitative study (Longitudinal generalized	Secondary data	This article assesses the impact of COVID-19 lockdown on the mental well-being of older adults who are housed in nursing homes.

No.	Title	Population of interest	First author	Year	Country	Study design/type	Data source	Brief description
	Data-Driven Approach in New Brunswick					estimating equations)		
103	Effect of COVID-19 lockdown on hospital admissions and mortality in rural KwaZulu-Natal, South Africa: interrupted time series analysis	Low-income populations	McIntosh, A.	2021	South Africa	Quantitative study (Interrupted time-series analysis)	Secondary data	The article assesses the effect of COVID-19 lockdown on all-cause hospital admissions and mortality.
104	Public Health measures to control the spread of COVID-19 amongst the migrant population in Malta	Migrant workers	Melillo, T.	2020	Malta	Single country case study	Secondary data	It briefly shows that the COVID-19 policy measures for the general population in Malta were not always accessible or feasible for migrant workers.
105	Socially distant and out of reach: Unintended consequences of COVID-19 prevention efforts on transgender and gender non-binary populations in Puerto Rico	People from sexual and gender minorities	Melin, K.	2021	USA	Observational study (qualitative)	Secondary data	The article describes the unintended consequences of COVID-19 policy responses on transgender and nonbinary populations.
106	Reopening Schools in the Context of COVID-19: Health and Safety Guidelines from Other Countries. Policy Brief.	Children/Students	Melnick, H.	2020	USA	Policy Brief	Secondary data	This brief describes health and safety guidelines in China, Denmark, Norway, Singapore, and Taiwan to inform plans for school reopening in the US.
107	Effect of the COVID-19 lockdown on disease recognition and utilisation of healthcare services in the older population in Germany: a cross-sectional study	Older people	Michalowsky, B.	2021	Germany	Cross-sectional observational study	Secondary data	This article examines how the older people are responding to the lockdown in terms of seeking healthcare providers and facilities.
108	Reducing SARS-CoV-2 transmission in the UK: A behavioural science approach to identifying options for increasing adherence to social distancing and shielding vulnerable people	General vulnerable	Michie, S.	2020	UK	Descriptive research	Secondary data	It argues for a behavioural science approach to inform policy makers in UK to enhance adherence to social distancing and protect vulnerable populations.
109	Social Distancing During COVID-19: Will it Change the Indian Society?	Older people; victims of domestic violence; low-income populations	Mishra, M.	2020	India	Single country case study (Descriptive research)	Secondary data	It presents the impact of social distancing on the Indian society amid the COVID-19 pandemic.

No.	Title	Population of interest	First author	Year	Country	Study design/type	Data source	Brief description
110	COVID-19 and Social Mobility Impact Brief #1: School Shutdown. Research Brief.	Students, including students from low-income families	Montacute, C.	2020	UK	Research brief	Secondary data	The brief discusses issues that school aged pupils face, including the resources available to them and the impact of school closure on the schoolwork completion.
111	Unspoken inequality: how COVID-19 has exacerbated existing vulnerabilities of asylum-seekers, refugees, and undocumented migrants in South Africa	Refugees	Mukumbang, F.C.	2020	South Africa	Comment	Secondary data	This commentary shares about the worsening repercussions for the displaced persons in South Africa during COVID-19.
112	Contact Tracing and the COVID-19 Response in Africa: Best Practices, Key Challenges, and Lessons Learned from Nigeria, Rwanda, South Africa, and Uganda	Low-income populations	Nachega, J. B.	2021	Africa - Nigeria, Rwanda, South Africa, and Uganda	Perspective piece	Secondary data	The article discusses best practices of COVID-19 contract tracing in Nigeria, Rwanda, South Africa, and Uganda, and their key challenges and lessons learned.
113	Simulation-Based Estimation of SARS-CoV-2 Infections Associated with School Closures and Community-Based Nonpharmaceutical Interventions in Ontario, Canada	Students, including students from low-income background	Naimark, D.	2021	Canada	Model simulation	Secondary data	The article simulates how COVID-19 infections relate with school closures and compares it with how COVID-19 infections relate with other community-based nonpharmaceutical interventions.
114	"Physical Sex Is Over for Now": Impact of COVID-19 on the Well-Being and Sexual Health of Adolescent Sexual Minority Males in the US	People from sexual and gender minorities	Nelson, K. M.	2020	USA	Mixed methods (survey with qualitative and quantitative questions)	Primary survey data	The article explores the impact of COVID-19's on adolescent sexual minority males' well-being and sexual health.
115	Cross-sectional survey of depressive symptoms and suicide-related ideation at a Japanese national university during the COVID-19 stay-home order	Children/Students	Nomura, K.	2021	Japan	Cross-sectional study (Survey and statistical analysis)	Primary survey data	The article examines the mental health issues, including depression and suicide-related ideation, among university students in Japan during the stay-at-home orders.
116	Distance Learning: Challenges Providing Services to K-12 English Learners and Students with Disabilities during COVID-19. Report to Congressional Committees. GAO-21-43	Students with Disabilities	Nowicki, J. M.	2020	USA	Government report	Primary interview data	It illustrates the challenges when offering distance learning to K-12 students with disabilities and English learners.

No.	Title	Population of interest	First author	Year	Country	Study design/type	Data source	Brief description
117	Early impacts of covid-19 and government's intervention and prevention actions in South Africa	Migrant workers; students	Nyamukamba, P.	2020	South Africa	Literature review	Secondary data	The article examines the effects of COVID-19 on socio-economic conditions in South Africa and what policy measures that the government to mitigate the negative effects and control the virus spread.
118	Exploring the impact of COVID-19 pandemic lockdown on informal settlements in Tshwane Gauteng Province, South Africa	Low-income populations, particularly people living in informal settlements and their children	Nyashanu, M.	2020	South Africa	Exploratory qualitative study (Interview and interpretive phenomenological analysis)	Primary interview data	The article investigates the impact of the COVID-19 lockdown on populations living in informal settlements.
119	An Informal Education Intervention in Response to the Covid-19 Pandemic: Homework Mentorships in a Berlin Refugee Shelter	Refugees	O'Connell, C.	2021	Germany	Descriptive research (qualitative)	Secondary data	This article sheds light on the difficult situations of refugees before COVID-19 and how it has worsened during the pandemic. It subsequently proposes an educational solution to bridge the gap between the refugees and their access to education.
120	Umuntu ngumuntu ngabantu' (a person is a person because of other people): reflections on student's experiences of social isolation and the impact of a peer to peer mental health support group during covid-19 lockdown in South Africa	Students, including students from low-income families	Obuaku-Igwe, C.	2020	South Africa	Qualitative study (Participatory health approach)	Primary data	The article examines the effect of social isolation incurred by the COVID-19 lockdown on the mental health of undergraduate students in South Africa, and the potential usefulness of peer-to-peer mental health support groups.
121	The Homeless, Inmates and Refugees in Africa in the Face of COVID-19 Outbreak	Low-income populations (especially the homeless); refugees; people in prison	Oladimeji, O.	2020	Africa	Editorial	Secondary data	It discusses the well-being and care for the homeless, people in prison and refugees amid the COVID-19 crisis in Africa and compares COVID-19 policy responses taken by the African countries with those taken by the western countries.
122	Determining the optimal strategy for reopening schools, the impact of test and trace interventions, and the risk of occurrence of a second COVID-19 epidemic wave in the UK: a modelling study	Children/Students	Panovska-Griffiths, J.	2020	UK	Model simulation	Secondary data	The article simulates policy scenarios for school reopening in the UK, involving physical distancing, testing and contact tracing and isolated of diagnosed cases.
123	Early intervention reduces the spread of covid-19 in long-term care facilities in the Republic of Korea	Older people	Park, S. Y.	2020	South Korea	Comparative case study (Comparing	Secondary data	It highlights that early detection and intervention is the most effective way to control the spread of COVID-19.

No.	Title	Population of interest	First author	Year	Country	Study design/type	Data source	Brief description
						three LTC facilities)		
124	"School Never Stops": Measures and Experience in Italian Schools during the COVID-19 Lockdown	Students, including students from low-income families	Pellegrini, M.	2020	Italy	Single country case study	Secondary data	The article describes the educational measures and experience in schools in Italy and discusses concerns about distance learning during the lockdown.
125	Ageing out of place in COVID-19 pandemic era: How does the situation look like for older refugees in camps?	Refugees	Peprah, P.	2020	Not specific	Letter	Secondary data	This letter shares about the experiences of the older people who lives in refugee camps during COVID-19.
126	The COVID-19 Pandemic and Mental Health of Refugees, Asylum Seekers, and Migrants	Refugees	Pinzón-Espinosa, J.	2021	Not specific	Corresponden ce	Secondary data	This article highlights the impact of COVID-19 on the mental well-being of displaced persons.
127	Covid-19: Local implementation of tracing and testing programmes could enable some schools to reopen	Children/Students	Pollock, A.M.	2020	UK	Letter	Secondary data	Rather than the blanket closure of schools, the article argues for school reopening with enhancement of contact tracing and testing among local communities.
128	Vulnerability to COVID-19-related Harms Among Transgender Women with and Without HIV Infection in the Eastern and Southern US	People from sexual and gender minorities	Poteat, T.C.	2020	USA	Descriptive research	Primary survey data	The article examines the vulnerability of women with and without HIV infection to COVID-19 related harms.
129	Social Distancing Effects on the Teaching Systems and Teacher Education Programmes in Brazil: Reinventing without Distorting Teaching	Children/Students	Prata-Linhares, M.M.	2020	Brazil	Cross-sectional documentary study	Secondary data	It presents a retrospective summary of the social distancing measures taken by Brazil and the effects of the social distancing on teacher education programmes and the teaching systems.
130	Impact of COVID-19 on the Socioeconomic life and Psychology of Transgenders: Insights from India	People from sexual and gender minorities	Priyadarshini, S.	2020	India	Qualitative study (content analysis)	Secondary data	The article examines the COVID-19 impact on the mental health and socioeconomic life of transgender people.
131	Is it really time to go back to school?	Children/Students	Puntis, J. W. L.	2021	UK	Letter	Secondary data	It discusses the feasibility of school reopening in the UK in June 2020, and argues that decisions on school reopening should take into account contextual factors.
132	The impact of COVID-19 control measures on social contacts and transmission in Kenyan informal settlements	Low-income populations, particularly people living in	Quaife, M.	2020	Kenya	Quantitative study (Survey and statistical analysis)	Primary survey data	The article evaluates how COVID-19 control policy measures affected social contacts and the basic reproduction number in informal settlements of Kenya.

No.	Title	Population of interest	First author	Year	Country	Study design/type	Data source	Brief description
		informal settlements						
133	Reopening Washington Schools 2020: District Planning Guide	Children/Students	Reykdal, C.	2020	USA	District planning guide	Secondary data	The report suggests school districts to provide face-to-face instructions as much as possible while maintaining health and safety measures.
134	Community action for people with HIV and sex workers during the COVID-19 pandemic in India	Sex workers	Reza-Paul, S.	2020	India	Perspective piece	Secondary data	It presents community-led efforts to provide health care and social protection for sex workers and people with HIV, through analysing the case of the Ashodaya Samithi organisation.
135	A Rapid Qualitative Assessment of the Impact of the COVID-19 Pandemic on a Racially/Ethnically Diverse Sample of Gay, Bisexual, and Other Men who Have Sex with Men Living with HIV in the US South	People from sexual and gender minorities	Rhodes, S.D.	2021	USA	Qualitative study (interview and ground theory building)	Primary interview data	It assesses the impact of the COVID-19 on a small sample of the GBMSM (gay, bisexual, and other men who have sex with men) population.
136	Effect of school closures on mortality from coronavirus disease 2019: old and new predictions	Children/Students	Rice, K.	2020	UK	Model simulation	Secondary data	The article simulates the COVID-19 spread in Great Britain and Northern Ireland and predicts the effectiveness of school closures on mitigating mortality.
137	Associations of Social Isolation with Anxiety and Depression During the Early COVID-19 Pandemic: A Survey of Older Adults in London, UK	Older people	Robb, C.E.	2020	UK	Cross-sectional study (Longitudinal survey and regression analysis)	Primary survey data	This article surfaces the correlation between social isolation and mental health conditions like depression and anxiety among the older people in UK.
138	Health and economic measures in response to the COVID-19 pandemic- Effect on street vendors	Low-income populations, particularly street vendors	Romero-Michel, J. C.	2021	Mexico	Case-control study (Interview and statistical analysis)	Primary interview data	The article describes the policy measures taken to contain the COVID-19 pandemic in Mexico and how they affected street vendors.
139	Modelling suggests limited change in the reproduction number from reopening Norwegian kindergartens and schools during the COVID-19 pandemic	Children/Students	Rypdal, M.	2021	Norway	Model simulation	Secondary data	The study simulates whether opening kindergartens or schools in Norway has an effect on the reproduction number (R) and whether the estimated R could be used to estimate transmission rates among children.

No.	Title	Population of interest	First author	Year	Country	Study design/type	Data source	Brief description
140	How the largest slum in India flattened the covid curve? A case study	Low-income populations, particularly people living in slums	Sahu, M.	2020	India	Single case study	Secondary data	The article analyses the COVID-19 management experiences of Daharavi, the largest slum in India, that successfully flattened the curve within 100 days.
141	Sexual and Gender Minority Stress Amid the COVID-19 Pandemic: Implications for LGBTQ Young Persons' Mental Health and Well-Being	People from sexual and gender minorities	Salerno, J. P.	2020	Not specific	Comment	Secondary data	It shows concerns about the potential effects of the COVID-19 pandemic on the mental health of LGBTQ young persons.
142	A plea to apply principles of quarantine ethics to prisoners and immigration detainees during the COVID-19 crisis	People in prison	Schotland, S. D.	2020	USA	Qualitative study	Secondary data	It argues that enhanced social distancing measures should be taken to ensure the safety and healthcare of people in prison and immigration detainees.
143	Compounding education disengagement: COVID-19 lockdown, the digital divide and wrap-around services	Children/Students	Seymour, K.	2020	Australia	Viewpoint piece	Secondary data	It shows the confounding effect of digital inequality on students who were already vulnerable and disengaged from secondary education during the COVID-19 lockdown.
144	Reductions in mortality resulting from COVID-19 quarantine measures in China	Older people	Shen, J.	2021	China	Quantitative study (time series analysis)	Secondary data	This article reaffirms that the measures undertaken by the Chinese government is effective in lowering mortality rates.
145	Social distancing, social justice, and risk during the COVID-19 pandemic	Low-income populations (particularly the homeless); migrant workers	Silva, D. S.	2020	Not specific	Comment	Secondary data	It presents the risks associated with COVID-19 and social distancing measures faced by marginalised groups and calls for social justice.
146	Impacts of the Covid-19 lockdown and relevant vulnerabilities on capability well-being, mental health and social support: an Austrian survey study	General vulnerable	Simon, J.	2021	Austria	Cross-sectional study (survey and regression analysis)	Primary survey data	The article examines the impact of lockdown in Austria and existing vulnerabilities on people's mental health, and social connections.
147	Capability impacts of the Covid-19 lockdown in association with mental well-being, social connections and existing vulnerabilities: an Austrian survey study	General vulnerable	Simon, J.	2020	Austria	Cross-sectional study (Survey and regression analysis)	Primary survey data	The article examines the impact of lockdown in Austria on people's mental health, and social connections.
148	Impacts of introducing and lifting nonpharmaceutical interventions on COVID-19 daily growth rate and compliance in the United States	Low-income populations; ethnic minority	Singh, S.	2021	USA	Quantitative study (Difference-in-differences method)	Secondary data	The article assesses the impact of nonpharmaceutical measures on the COVID-19 case growth and compliance with social distancing.

No.	Title	Population of interest	First author	Year	Country	Study design/type	Data source	Brief description
149	Income and Employment Changes Under COVID-19 Lockdown: A Study of Urban Punjab	Low-income populations; ethnic minority	Singh, I.	2020	India	Mixed methods (Interview and statistical analysis)	Primary interview data	The article shows changes in income and employment at various phases of the lockdown in India.
150	Impact of COVID-19 and lockdown on mental health of children and adolescents: A narrative review with recommendations	Students, including students with disabilities	Singh, S.	2020	Global	Narrative review	Secondary data	The article reviews the impact of COVID-19 and resultant lockdowns on mental health among adolescents and children.
151	Effects of the COVID-19 pandemic and lockdown in Spain: comparison between community controls and patients with a psychiatric disorder. Preliminary results from the BRIS-MHC STUDY	People with disabilities, particularly people with psychiatric disability	Solé, B.	2021	Spain	Cross-sectional study (Survey and statistical analysis)	Primary survey data	The article presents the difference regarding effects of the COVID-19 and lockdown between patients with mental illness and community controls.
152	An analysis of school absences in England during the Covid-19 pandemic	Children/Students	Southall, E.	2021	UK	Quantitative study	Secondary data	The article investigates student absences at schools due to COVID-19 infections after the school reopening in England.
153	Challenges posed by COVID-19 to refugee camps on the Greek islands: We are all humans after all	Refugees	Spernovasilis, N.	2020	Greece	Letter	Secondary data	This article surfaces the difficulties in managing COVID-19 amongst refugees in Greek Islands.
154	Sex in the Time of COVID-19: Results of an Online Survey of Gay, Bisexual and Other Men Who Have Sex with Men’s Experience of Sex and HIV Prevention During the US COVID-19 Epidemic	People from sexual and gender minorities	Stephenson, R.	2021	USA	Cross-sectional study (survey and regression analysis)	Primary survey data	The article shows changes in sexual behaviours and availability of HIV prevention measures for the GBMSM (gay, bisexual, and other men who have sex with men) population.
155	What the COVID-19 school closure left in its wake: Evidence from a regression discontinuity analysis in Japan	Children/Students	Takaku, R.	2021	Japan	Quantitative study (Regression discontinuity analysis)	Primary survey data	The article analyses the impact of elementary school closures on children and their families.
156	Effect of the Pandemic and Lockdown on Mental Health of Children	Children/Students	Thakur, K.	2020	India	Letter	Secondary data	It addresses the nagative effect of the lockdown on children's mental health and use of internet and TV.
157	Inequality in access to health and care services during lockdown - Findings from the	Ethnic minority	Topriceanu, C.-C.	2020	UK	Quantitative study (using longitudinal survey data	Primary survey data	The article displays the inequality in access to healthcare services amid the lockdown in the UK.

No.	Title	Population of interest	First author	Year	Country	Study design/type	Data source	Brief description
	COVID-19 survey in five UK national longitudinal studies					and regression analysis)		
158	Family Violence and COVID-19: Increased vulnerability and reduced options for support	Victims of domestic violence	Usher, K.	2020	Not specific	Editorial	Secondary data	It shows social isolation to control the COVID-19 may aggravate domestic violence and suggests reimagining networks to support people at risks of domestic violence during the pandemic.
159	Effectiveness of three versus six feet of physical distancing for controlling spread of COVID-19 among primary and secondary students and staff: A retrospective, state-wide cohort study	Children/Students	van den Berg, P.	2021	USA	Quantitative study (Regression analysis)	Secondary data	COVID-19 infected cases among students and staff in Massachusetts public schools are analysed when physical distance is implemented at three and six feet.
160	Mitigation of COVID-19 using social distancing of the elderly in Brazil: The vertical quarantine effects in hospitalizations and deaths	Older people	Venturieri, V. R.	2021	Brazil	Model simulation	Secondary data	It reveals that isolating the older people allows for a better control of the COVID-19 situation.
161	School closure and management practices during coronavirus outbreaks including COVID-19: a rapid systematic review	Children/Students	Viner, R. M.	2020	Not specific	Literature review	Secondary data	A rapid systematic review was conducted to identify the effectiveness of school closures and other policy practices in school settings in response to coronavirus outbreaks (i.e., SARS, COVID-19, or Middle East respiratory syndrome).
162	Contact with older people, ageism, and containment behaviours during the COVID-19 pandemic	Older people	Visintin, E. P.	2021	Italy	Quantitative study (survey and correlation analysis)	Primary survey data	It shows the link of containment behaviours to contact with the older people and ageism before the COVID-19 pandemic and
163	How Should Massachusetts Reopen Its K-12 Schools in the Fall? Lessons from Abroad and Other States. White Paper No. 211	Children/Students	von Schroeter, M.	2020	USA	White paper	Secondary data	The article reviews policy measures and experiences among other states and countries to inform reopening of K-12 schools in Massachusetts.
164	Remote data collection during COVID-19 restrictions: an example from a refugee and asylum-seeker participant group in the UK	Refugees	Walker, L.	2021	UK	Descriptive research (qualitative)	Primary survey data	This article explores the change in the method of gathering data due to the COVID-19 measures by utilising examples from voluntary people who are displaced in the UK.

No.	Title	Population of interest	First author	Year	Country	Study design/type	Data source	Brief description
165	South Africa's COVID-19 lockdown dilemma	Low-income populations	Ward, M.	2020	South Africa	Single country case study	Secondary data	The article presents the difficulties faced by South Africa during the COVID-19 accounting for the unintended economic consequences of the lockdown.
166	The impossibility of social distancing among the urban poor: the case of an Indian slum in the times of COVID-19	Low-income populations, particularly people living in slums	Wasdani, K. P.	2020	India	Observational study (single case analysis)	Primary observational data	The article illustrates the challenges faced by the residents in an Indian slum to follow the containment measures.
167	Social distancing responses to COVID-19 emergency declarations strongly differentiated by income	Low-income populations	Weill, J. A.	2020	USA	Quantitative study (Panel regression analysis)	Secondary data	The article examines the difference in adherence to social distancing among populations at different income levels.
168	Managing Public Education Resources during the Coronavirus Crisis: Practical Tips and Considerations for School District Leaders. Crisis Response Resource	Children/Students	Willis, J.	2020	USA	Policy Brief	Secondary data	This brief offers practical tips and guidance for school district and charter school leaders to manage public education resources strategically amid COVID-19 and the incurred school closures
169	Managing Public Education Resources in California during and after the Coronavirus Crisis: Practical Tips and Considerations for School District and Charter School Leaders. Crisis Response Resource	Children/Students	Willis, J.	2020	USA	Policy Brief	Secondary data	This brief offers practical tips and guidance for school district and charter school leaders in California to manage public education resources strategically amid COVID-19 and the incurred school closures
170	The Potential Impact of COVID-19 on Student Learning and How Schools Can Respond	Children/Students	Wyse, A.E.	2020	USA	Quantitative study	Secondary data	It examines the potential impact of the COVID-19 pandemic on student learning and discusses the measures that schools can take to facilitate student learning
171	Anxiety and Depression Among Health Sciences Students in Home Quarantine During the COVID-19 Pandemic in Selected Provinces of Nepal	Children/Students	Yadav, R. K.	2021	Nepal	Cross-sectional study (Survey and statistical analysis)	Primary survey data	The article examines the anxiety and depression issues among students at home quarantine, particularly health science students.
172	Flexibility in individual funding schemes: How well did Australia's National Disability Insurance Scheme support remote learning for students with disability during COVID-19?	Students with disabilities	Yates, S.	2020	Australia	Single case study (survey with qualitative and quantitative questions)	Primary survey data	The article analyses the Australia's National Disability Insurance Scheme (NDIS), an individual funding scheme, and how it worked to help students with disability to adapt to remote learning during the COVID-19.

No.	Title	Population of interest	First author	Year	Country	Study design/type	Data source	Brief description
173	Determination of Educational/Academic and Some Social Behavioral Trends of Elementary School Students in Turkey during Coronavirus (COVID-19) Pandemic Days.	Children/Students	Yayci, L.	2021	Turkey	Descriptive study (Survey and statistical analysis)	Primary survey data	The article examines educational/academic and social behaviors of students at elementary schools when schools were closed and they were attending distance learning.
174	Stressors and coping strategies of migrant workers diagnosed with COVID-19 in Singapore: a qualitative study	Migrant workers	Yee, K.	2021	Singapore	Observational study (qualitative)	Primary interview study	The article examines the mental stress experienced by migrant workers during the COVID-19 and their coping strategies.
175	COVID-19 and Unequal Social Distancing across Demographic Groups	Low-income populations	Yilmazkuday, H.	2020	USA	Quantitative study (Panel regression analysis)	Secondary data	The article illustrates that how adherence to social distancing have differed across demographic groups in the US.
176	Landscape Measure: Animating the University Campus to Promote Social Distancing	Children/Students	Yui, L.	2020	USA	Descriptive report	Secondary data	It suggests transforming university lawns to outdoor classrooms to promote social distancing.
177	Association of the Timing of School Closings and Behavioural Changes with the Evolution of the Coronavirus Disease 2019 Pandemic in the US	Children/Students	Zimmerman, F. J.	2021	USA	Cross-sectional study (Interrupted time series analysis)	Secondary data	The article examines how the COVID-19 incidence and mortality relate to the timing of school closures, and voluntary behavioral changes consistent with social distancing.
178	Pandemic school closures: risks and opportunities	Children/Students	No author name available	2020	Not specific	Editorial	Secondary data	It addresses risks of school closures in response to COVID-19 and the opportunities for the youth to develop their resilience and adaptability to the changing world.
179	Beginning the Conversation: Responding to Domestic Violence and Abuse in Higher Education Communities during COVID-19 Pandemic.	Victims of domestic violence	No author name available	2020	UK	Policy brief	Secondary data	It discusses measures to safeguard staff and students in higher education communities who are at greater risk of domestic violence and abuse.

Supplementary Table 2. List of studies added in 2022

No.	Title	Population of interest	First author	Year	Country	Study design/type	Data source	Brief description
1	Psychological effects of quarantine on Syrian refugees, compared to the Jordanian populations	Refugees	Al-Awaida, W.	2022	Jordan	Cross-sectional study (Survey and statistical analysis)	Primary data	It examined the effects of the COVID-19 quarantine on psychological status of Jordanian populations and Syrian refugees in Jordan.
2	Impact of COVID-19 lockdown on children with asthma in Jordan: A parental questionnaire	Student/Childre n	Al-Iede, M.	2021	Jordan	Cross-sectional study (Survey and statistical analysis)	Primary data	It examined how the national COVID-19 lockdown in Jordan impacted children with asthma, including a decrease in emergency presentations and hospitalisation rate for acute asthma exacerbations.
3	How does Covid-19 affect urban slums? Evidence from settlement leaders in India	Low-income populations	Auerbach, A. M.	2021	India	Cross-sectional study (Survey and statistical analysis)	Primary data	It explored how the COVID-19 pandemic in India affected urban slums and how slum leaders addressed the COVID-19 related risks in their communities.
4	Commercial sex work during novel coronavirus (SARS-CoV-2) era in the niger delta region: Relationships between knowledge, preventive practice, and transmission potential	Sex worker	Avwioro, G.	2021	Nigeria	Cross-sectional study (Survey and statistical analysis)	Primary data	It explored the COVID-19 related knowledge, awareness and prevention behaviors among female sex workers and addressed that sex work had high COVID-19 transmission potentials.
5	COVID Social Distancing and the Poor: An Analysis of the Evidence for England	Low-income populations	Basu, P.	2022	England	Quantitative study (Panel regression analysis)	Secondary data	It evaluated whether the low-income populations differred from other populations regarding their compliance with social distancing.
6	Impact of COVID-19 Mitigation efforts on adults with serious mental Illness	People with disabilities	Benjenk, I.	2021	USA	Qualitative study (interviews and thematic analysis)	Primary data	It explored the impact of COVID-19 mitigation policies on adults with serious mental illness, including decreased access to health care services.
7	Impact of containment and mitigation measures on children and youth with SD during the COVID-19 pandemic: Report from the ELENA cohort	Student/Childre n	Berard, M.	2021	France	Cross-sectional study (Survey and statistical analysis)	Primary data	It examined how the COVID-19 containment and mitigation measures impacted the behaviors (e.g., sleep, nutrition, communication) among youth and children with autism specturm disorders.

No.	Title	Population of interest	First author	Year	Country	Study design/type	Data source	Brief description
8	Psychosocial and mental health during the COVID-19 lockdown: A rapid qualitative study in migrant shelters at the Mexico-United States border	Migrant workers	Bojórquez, I.	2021	Mexico	Qualitative study (interviews and content analysis)	Primary data	The study examined the mental and psychological health consequences of the COVID-19 and related lockdown measures among migrant workers in shelters.
9	The impact of COVID-19 measures on parent-reported restricted and repetitive behaviours in pre-school children with autism	Student/Children with disabilities	Boterberg, S.	2022	Belgium	Cross-sectional study (Survey and regression analysis)	Primary data	The study examined the impact of COVID-19 restrictive measures on behaviours of pre-school children with autism.
10	The effects of living and learning conditions on the health-related quality of life of children and adolescents during the COVID-19 lockdown in the French Grand Est region	Student/Children	Bourion-Bédès, S.	2022	France	Cross-sectional study (survey and regression analysis)	Primary data	It analysed how living and learning conditions related to children and adolescents' health-related quality of life during the COVID-19 lockdown.
11	Unintended Consequences of COVID-19 Social Distancing Among Older Adults With Kidney Disease	Older people	Bowling, C. B.	2022	USA	Cross-sectional study (survey and regression analysis)	Primary data	It showed that COVID-19 social distancing measures had unintended health consequences among older people with chronic kidney diseases.
12	Life in lockdown: A telephone survey to investigate the impact of COVID-19 lockdown measures on the lives of older people (≥75 years)	Older people	Brown, L.	2021	UK	Cross-sectional study (Survey and statistical analysis)	Primary data	It examined how the COVID-19 lockdown measures impacted older people (aged 75 and above), including physical and mental health, and access to services.
13	Depressive symptoms in higher education students during the COVID-19 pandemic: the role of containment measures	Student/Children	Buffel, V.	2022	Global	Cross-sectional study (survey and regression analysis)	Primary data	It investigated how depression among higher education students related to various containment measures responding to the COVID-19 pandemic.
14	Effects of the COVID-19 lockdown on mental health in a UK student sample	Student/Children	Catling, J. C.	2022	UK	Cross-sectional study (Survey and statistical analysis)	Primary data	It examined effects of the lockdown in UK on students' mental health during the COVID-19 pandemic.
15	Impact of COVID-19 and lockdown on mental health and future orientation among young adult asylum seekers in Italy: A mixed-methods study	Refugee	Ceccon, C.	2022	Italy	Mixed methods study	Primary data	The study reported the impact of COVID-19 and related lockdown on the perceived mental health and future orientation among asylum seekers in Italy.

No.	Title	Population of interest	First author	Year	Country	Study design/type	Data source	Brief description
16	The impact of COVID-19 restrictions and care home strategies on residents with dementia as experienced by family carers in Italy	People with disabilities	Chirico, I.	2022	Italy	Qualitative study (interviews and thematic analysis)	Primary data	It investigated the impact of COVID-19 restrictive measures and care home strategies on care home residents with dementia.
17	Life during lockdown: a qualitative study of low-income New Zealanders' experience during the COVID-19 pandemic	Low-income populations	Choi, K.	2021	New Zealand	Qualitative study (interviews and content analysis)	Primary data	It described the livelihood of low-income populations during the COVID-19 lockdown in New Zealand.
18	Children's psychological well-being and problem behavior during the COVID-19 pandemic: An online study during the lockdown period in Germany	Student/Childre n	Christner, N.	2021	Germany	Cross-sectional study (Survey and regression analysis)	Primary data	The study reported the psychological well-being and problem behaviours among children (aged 3-10) and their parents during the COVID-19 pandemic lockdown in Germany.
19	Impact of the COVID-19 pandemic on vulnerable groups, including homeless persons and migrants, in France: A qualitative study	Migrant workers	Crouzet, L.	2022	France	Qualitative study (interviews and content analysis)	Primary data	It explored the impact of COVID-19 on homeless persons and migrant workers.
20	Psycho-social impact of social distancing and isolation due to the coVid-19 containment measures on patients with physical disabilities	People with disabilities	Dalise, S.	2021	Italy	Cross-sectional study (Survey and statistical analysis)	Primary data	The study identified the impact of the COVID-19 pandemic on the perceived mental health, social condition and illness among patients with physical disabilities.
21	The impact of physical distancing on socially vulnerable people needing care during the COVID-19 pandemic in the Netherlands	General vulnerable populations	de Vries, D.	2022	Netherlands	Qualitative research (interviews and surveys)	Primary data	It reported the impact of physical distancing on populations that were socially vulnerable and neended care during the first COVID-19 lockdown in the Netherlands.
22	Impact of the COVID-19 italian lockdown on the physiological and psychological well-being of children with fragile x syndrome and their families	Student/Childre n	Di Giorgio, E.	2021	Italy	Cross-sectional study (Survey and statistical analysis)	Primary data	The study analysed the impact of the COVID-19 lockdown on the mental and physiological well-being of children (aged 2-16) who had fragile X-syndrome and their caregivers.
23	Intimate partners violence against women during a covid-19 lockdown period: Results of an online survey in 7 provinces of the Democratic Republic of Congo	Victims of domestic violence	Ditekemena, J. D.	2021	Democratic Republic of Congo	Cross-sectional study (Survey and regression analysis)	Primary data	The study explored the levels of initimate partner violence against women during the COVID-19 lockdown in the Democratic Republic of Congo and the associated determiants.
24	COVID-19 outbreak in a state prison: a case study on the implementation of key public	People in prison	Duarte, C.	2022	USA	Case study	Secondary data	The study explored the factors that contributed to the containment and prevention of the COVID-19 outbreak in a Califonia prison.

No.	Title	Population of interest	First author	Year	Country	Study design/type	Data source	Brief description
	health recommendations for containment and prevention							
25	Intersections between COVID-19 and socio-economic mental health stressors in the lives of South African adolescent girls and young women	Adolescent	Duby, Z.	2022	South Africa	Mixed methods (Survey and qualitative interviews)	Primary data	It examined impacts of the COVID-19 on adolescent girls and young women in South Africa by discussing how COVID-19 intersected with pre-existing social-economic factors.
26	The Impact of COVID-19 Confinement on Cognition and Mental Health and Technology Use Among Socially Vulnerable Older People: Retrospective Cohort Study	Older people	Dura-Perez, E.	2022	Spain	Retrospective Cohort Study	Primary data	It examined the impact of COVID-19 restrictive measures on socially vulnerable older people in Spain, including effects on cognition, mental health and technology use.
27	Poverty on the rise: The impact of the COVID-19 lockdown on the informal sector of Gweru, Zimbabwe	Low-income populations	Dzawanda, B.	2021	Zimbabwe	Qualitative study (interviews and thematic analysis)	Primary data	The study stated the negative impact of the COVID-19 lockdown on the informal sector in Gweru and the responsive measures taken by the government.
28	An exploration of the impact of SARS-CoV-2 (COVID-19) restrictions on marginalised groups in the UK	Ethnic minority	Eshareturi, C.	2021	UK	Qualitative study (interviews and thematic analysis)	Primary data	It explored the impact of COVID-19 restrictive measures on the health and well-being status of marginalised populations who relied on closed social and community support services.
29	Impact of COVID-19 lockdown on glucose control of elderly people with type 2 diabetes in Italy	Older people	Falcetta, P.	2021	Italy	Retrospective Cohort Study	Primary data	It analysed the impact of Italian COVID-19 lockdown on glucose control among older people with type 2 diabetes.
30	Impact of COVID-19 lockdown on children with developmental disabilities and their parents	People with disabilities	Folostina, R.	2021	Romania	Cross-sectional study (Survey and statistical analysis)	Primary data	The study explored how the COVID-19 lockdown impacted the lifestyle and health conditions (mental and physical) among children with developmental disabilities and their parents.
31	Vulnerable migrants’ access to healthcare in the early stages of the COVID-19 pandemic in the UK	Migrant workers	Fu, L.	2022	UK	Mixed methods study	Secondary data	The study explored the vulnerable migrant workers' living conditions and accessibility to healthcare in the early stages of COVID-19 in UK.
32	Lockdown strictness and mental health effects among older populations in Europe	Older people	García-Prado, A.	2022	Europe	Quantitative study (difference-in-difference regression analysis)	Secondary data	It tested the causal effects of lockdown policies in Europe on mental health of adults aged 50 and above during the first COVID-19 pandemic wave.
33	Examining the Impacts of the Coronavirus Pandemic and Social Distancing on the Health	People with disabilities	Goddard, K. S.	2022	USA	Qualitative study (interviews	Primary data	It reported the negative impacts of COVID-19 and its social distancing measures on health and access to health care services among populations with mobility disabilities.

No.	Title	Population of interest	First author	Year	Country	Study design/type	Data source	Brief description
	of People With Mobility Disabilities					and thematic analysis)		
34	Reopening schools in a context of low COVID-19 contagion: consequences for teachers, students and their parents	Student/Children	Godøy, A.	2022	Norway	Quantitative study (difference-in-difference regression analysis)	Secondary data	It analysed the impact of school reopening on COVID-19 incidence among students, teachers and parents.
35	Life in lockdown: impact of COVID-19 lockdown measures on the lives of visually impaired school-age children and their families in India	Student/Children	Gothwal, V. K.	2022	India	Qualitative research (semi-structured interviews and content analysis)	Primary data	It investiged the impact of COVID-19 lockdown on school-age visually impaired children and their parents in India.
36	Decreased Physical Activity Among Youth Resulting From COVID-19 Pandemic-Related School Closures: Natural Experimental Study	Student/Children	Grimes, A.	2022	USA	Natural experimental design	Primary data	The natural experimental study showed that physical activity decreased among youth from before to during COVID-19–related school closures.
37	Venerable vulnerability or remarkable resilience? A prospective study of the impact of the first wave of the COVID-19 pandemic and quarantine measures on loneliness in Swedish older adults with home care	Older people	Gustafsson, P. E.	2022	Sweden	Prospective pretest–posttest and controlled interrupted time series designs	Secondary data	The study examined the impact of the COVID-19 and related quarantine measures on the mental health, particularly loneliness, of older people with home care.
38	Emotional and Mental Wellbeing Following COVID-19 Public Health Measures on People Living With Dementia and Carers	People with disabilities	Hanna, K.	2022	UK	Qualitative study (interviews and thematic analysis)	Primary data	It explored the impact of COVID-19 and its public health restrictions on the mental health of people living with dementia and carers.
39	Domestic Violence During a Global Pandemic: Lockdown Policies and Their Impacts Across Guatemala	Victims of domestic violence	Iesue, L.	2021	Guatemala	Quantitative study (Regression analysis)	Secondary data	It evaluated how the COVID-19 lockdown and reopening policies impacted domestic violence.
40	Unsafe at home: the increased trend of domestic violence during the COVID-19 pandemic in Bangladesh	Victims of domestic violence	Jahid, A.	2022	Bangladesh	Viewpoint	Secondary data	It summarised the COVID-19 and domestic violence issue in Bangladesh.
41	Covid-19 lockdown: A greater impact on women	Victims of domestic violence	Jain, B.	2022	Global	Qualitative study (narrative research)	Secondary data	It discussed the impact of COVID-19 lockdown on women, including risks of domestic violence and mental distress.

No.	Title	Population of interest	First author	Year	Country	Study design/type	Data source	Brief description
42	The effect of COVID-19-related school closures on students' well-being: Evidence from Danish nationwide panel data	Student/Children	Jensen, S. S.	2021	Denmark	Quantitative study (difference-in-difference regression analysis)	Secondary data	It examined the effect of school closures due to the COVID-19 pandemic on the well-being of students in Denmark.
43	Effect of Covid-19 lockdown on Indian children with autism	Student/Children	Kaur, R.	2022	India	Cross-sectional study (survey and regression analysis)	Primary data	It explored the negative effects of COVID-19 lockdown in India on children with autism.
44	The impact of lockdown during the COVID-19 pandemic on mental and social health of children and adolescents	Student/Children	Luijten, M. A. J.	2021	Netherlands	Cross-sectional study (Survey and regression analysis)	Primary data	The study examined how the COVID-19 lockdown impacted the mental and social health among children and adolescents.
45	Impact of the COVID-19 lockdown on intimate partner violence: Issues of non-reporting in Bangladesh	Victims of domestic violence	Mahmood, N.	2022	Bangladesh	Quantitative study (Poisson regression analysis)	Secondary data	It analysed how the COVID-19 lockdown in Bangladesh affected intimate partner violence reporting.
46	Impact of COVID-19 lockdown on health parameters and muscle strength of older women: A longitudinal study	Older people	Marcos-Pardo, P. J.	2022	Spain	Longitudinal study	Primary data	It tested the impact of Spain's COVID-19 lockdown on health status of women aged from 60 to 70.
47	The Impact of COVID-19 Restrictions on the Healthy Eating and Movement Behaviors of 0–12-Year-Old Children in Western Sydney, Australia	Student/Children	McNicholas, J.	2022	Australia	Cross-sectional study (survey and statistical analysis)	Primary data	It analysed how COVID-19 restrictions impacted movement and eating behaviors of children (aged 0-12).
48	Influence of COVID-19 pandemic lockdown on a sample of Egyptian children with Down syndrome	Student/Children	Meguid, N. A.	2022	Egypt	Cohort prospective study (Survey and logistic regression analysis)	Primary data	It investiged how the COVID-19 lockdown affected children with Down syndrome in Egypt.
49	The mental health crisis during the COVID-19 pandemic in older adults and the role of physical distancing interventions and social protection measures in 26 European countries	Older people	Mendez-Lopez, A.	2022	26 European countries	Quantitative study (regression analysis)	Secondary data	It examined how mental health of older people aged 50 and over related to COVID-19 policy responses and social protection measures.

No.	Title	Population of interest	First author	Year	Country	Study design/type	Data source	Brief description
50	Impact of quarantine due to COVID-19 pandemic on health and lifestyle conditions in older adults from Centro American countries	Older people	Mendoza-Ruvalcaba, N. M.	2022	Centro American countries	Cross-sectional study	Primary data	The study analysed the impact of COVID-19 quarantine on mental health, physical health and lifestyle among adults aged 60 and over in Centro American countries.
51	Caregiver-Reported Changes in the Socioemotional Wellbeing and Daily Habits of Children With Special Educational Needs During the First COVID-19 National Lockdown in the United Kingdom	Student/Children	Morgül, E.	2022	UK	Cross-sectional study (survey and regression analysis)	Primary data	It reported changes in socioemotional wellbeing and habits of children (aged 5-12 years) with special educational needs during UK's COVID-19 lockdown in the first wave.
52	Impact of COVID-19 Pandemic Lockdown on Mental Well-Being of Norwegian Adolescents During the First Wave—Socioeconomic Position and Gender Differences	Student/Children	Myhr, A.	2021	Norway	Cross-sectional study (Survey and regression analysis)	Primary data	The study evaluated the impact of the COVID-19 lockdown on adolescents' mental well-being, including loneliness and depressive symptoms, and how it differed across gender and socioeconomic groups.
53	Domesticated Poly-Violence Against Women During the 2020 Covid-19 Lockdown in South Africa	Victims of domestic violence	Nduna, M.	2021	South Africa	Qualitative study (narrative research)	Secondary data	It discussed the domesticated poly-violence issue experienced by womend during the COVID-19 lockdown.
54	Intimate partner violence during COVID-19 lockdown in Norway: the increase of police reports	Victims of domestic violence	Nesset, M. B.	2021	Norway	Quantitative study (pre-post statistical comparison)	Secondary data	It reported changes in frequency and characteristics of initimate partner violence reported in Norway before and during the COVID-19 lockdown.
55	The Homeless-Poor and the COVID-19 Stay-at-Home Policy of Government: Rethinking the Plight of Homelessness in Nigeria	Low-income populations	Obasi, C. O.	2021	Nigeria	Mixed methods study	Primary and secondary data	It examined the situations faced by the homeless poor when implementing the COVID-19 stay-at-home policy.
56	The impact of Covid-19-related distancing on the well-being of nursing home residents and their family members: a qualitative study	Older people	Paananen, J.	2021	Finland	Qualitative study (interviews and thematic analysis)	Primary data	It evaluated the COVID-19 related social distancing measures on the well-being of residents in nurding homes and their family members.
57	Depression deterioration of older adults during the first wave of the COVID-19 outbreak in Europe	Older people	Paccagnella, O.	2022	Europe	Quantitative study (logistic regression analysis)	Secondary data	It examined depression deterioration among adults aged 50 and above since the first COVID-19 pandemic wave in Europe.
58	COVID-19 and Its Effects on the Mental Health of People Living in Urban Slums in India	Low-income populations	Pandey, G.	2022	India	Mixed methods (interviews and	Primary data	The study examined the effects of COVID-19 on mental health of populations in urban slums in India.

No.	Title	Population of interest	First author	Year	Country	Study design/type	Data source	Brief description
						regression analysis)		
59	Effect of COVID-19 pandemic on older adults' emotion regulation and quality of life after lockdown in Italy	Older people	Pezzuti, L.	2021	Italy	Cross-sectional study (Survey and statistical analysis)	Primary data	It evaluated changes in older people's emotional regulation and quality of life after the COVID-19 lockdown in Italy.
60	HIV service delivery in the time of COVID-19: focus group discussions with key populations in India	Sex worker	Pollard, R.	2021	India	Qualitative study (Focus group discussion)	Primary data	It evaluated the impact of the COVID-19 on HIV service delivery among key populations.
61	Impact of lockdown and school closure on children's health and well-being during the first wave of COVID-19: A narrative review	Student/Childre n	Rajmil, L.	2021	Global	Narrative review	Secondary data	The study conducted a narrative literature review to summarise the impact of COVID-19 lockdown and school closures on the health and well-being among children in the first COVID-19 wave.
62	Impact of the COVID-19 National Lockdown on a Rural and Tribal Population of Tamil Nadu, Southern India: A Mixed-Methods Survey	Low-income populations	Ramesh, R. M.	2022	India	Mixed methods (Survey and focus group discussion)	Primary data	It explored how the COVID-19 lockdown in India impacted health, finances and livelihood of rural and tribal populations.
63	Impact of COVID-19 lockdown: Domestic and child abuse in Bridgend	Victims of domestic violence	Rengasamy, E. R.	2021	UK	Retrospective observational study	Secondary data	It examined how the COVID-19 lockdown in UK impacted incidence and reporting of domestic and child abuse.
64	The Medium-Term Changes in Health-Related Behaviours among Spanish Older People Lifestyles during Covid-19 Lockdown	Older people	Rodríguez-Gómez, I.	2022	Spain	Cohort prospective study	Primary data	The study evaluated changes in health-related behaviors among older adults, including diet quality, sedentary times, and physical activity, in the middle of and 7 months after the COVID-19 lockdown in Spain.
65	Epidemics, Lockdown Measures and Vulnerable Populations: A Mixed-Methods Systematic Review of the Evidence of Impacts on Mother and Child Health in Low-and Lower-Middle-Income Countries	Student/Childre n	Russo, G.	2021	Low- and lower-middle-income countries	Mixed-Methods Systematic Review	Secondary data	It summarised the impacts of epidemic related lockdown on the health status among women and children in low- and lower-middle-income countries.
66	Impact of COVID-19 and Lockdown on Children with ADHD and Their Families-An Online Survey and a Continuity Care Model	Student/Childre n	Shah, R.	2021	India	Cross-sectional survey study and online survey experiment	Primary data	The study evaluated the impact of the COVID-19 and lockdown among children with attention-deficit hyperkinetic disorders, and the feasibility of implementing a "text message-based" intervention.
67	The impact physical distancing during the COVID-19 pandemic on mental health	Student/Childre n	Sholihah, I. F.	2022	Global: Bangladesh, Belgium,	Systematic review	Secondary data	It reviewed COVID-19 physical distancing on mental health of adolescents.

No.	Title	Population of interest	First author	Year	Country	Study design/type	Data source	Brief description
	among adolescents: a systematic literature review				Swiss, Australia, USA, Ireland, Italy			
68	A national survey on COVID-19 second-wave lockdowns on older adults' mental wellbeing, health-seeking behaviours and social outcomes across Australia	Older people	Siette, J.	2021	Australia	Cross-sectional study (Survey and regression analysis)	Primary data	It evaluated the impact of the Australian lockdown in the second COVID-19 wave on quality of life (including mental wellbeing and health-seeking behaviours) among older people.
69	Impact of COVID-19 pandemic on the mental health of school-going adolescents: insights from Dhaka city, Bangladesh	Student/Children	Sifat, R. I.	2022	Bangladesh	Qualitative study (interviews and content analysis)	Primary data	It examined the impact of COVID-19 and its prevention measures on mental health among school-going adolescents.
70	Subjective Well-being Among Migrant Workers Residing in a Shelter Home During Lockdown: A Pilot Survey From North India	Migrant workers	Singh, G. P.	2021	India	Cross-sectional study (Survey and statistical analysis)	Primary data	It explored the impact of COVID-19 lockdown on perceived stress levels and well-being among migrant workers in a shelter home.
71	Explaining the Psychosocial Effects of COVID-19 Among Older Hong Kong Chinese People—A Qualitative Analysis	Older people	Sit, R. W. S.	2022	Hong Kong	Qualitative research (semi-structured interviews and ground theory building)	Primary data	It investigated effects of COVID-19 on psychosocial status among older people in Hong Kong.
72	COVID-19 induced impact on informal migrants in Bangladesh: a qualitative study	Migrant workers	Sohel, M. S.	2022	Bangladesh	Qualitative research (semi-structured interviews)	Primary data	It explored how COVID-19 induced income and occupation loss affected informal migrant workers.
73	Impact of covid-19 lockdown on physical and mental health of 5-12 years old children; from parents' perspective: A cross-sectional study	Student/Children	Tajane, I.	2021	India	Cross-sectional study (Survey and regression analysis)	Primary data	It identified the impact of COVID-19 lockdown in India on physical and mental health conditions of children aged 5-12 years.
74	COVID-19 and the shifting organisation of sex work markets in Singapore	Sex worker	Tan, R. K. J.	2021	Singapore	Mixed methods study	Primary data	It addressed that the COVID-19 and its lockdown measures have shifted the sex work markets in Singapore from in-person towards online sex work.
75	Older Europeans' experience of unmet health care during the COVID-19 pandemic (first wave)	Older people	Tavares, A. I.	2022	EU countries	Quantitative study (logistic regression analysis)	Secondary data	It examined the unmet health care among older adults in EU countries during the COVID-19.

No.	Title	Population of interest	First author	Year	Country	Study design/type	Data source	Brief description
76	The effects of COVID-19 restrictions on physical activity and mental health of children and young adults with physical and/or intellectual disabilities	Student/Children	Theis, N.	2021	UK	Cross-sectional study (Survey and statistical analysis)	Primary data	It addressed the negative effects of the COVID-19 lockdown on physical activity and mental health levels among children and young adults with disabilities.
77	Impact of COVID-19 Pandemic on Sexual Minority Populations in Brazil: An Analysis of Social/Racial Disparities in Maintaining Social Distancing and a Description of Sexual Behavior	Sexual & gender minority	Torres, T. S.	2021	Brazil	Cross-sectional study (Survey and statistical analysis)	Primary data	It assessed the impact of COVID-19 and its social distancing measures on sexual minority populations, including personal lives, sexual behavior and access to HIV related health services.
78	Social support and unmet needs among older trans and gender non-conforming people during the COVID-19 'lockdown' in the UK	Older people/Sexual & gender minority	Toze, M.	2021	UK	Cross-sectional study (Survey and statistical analysis)	Primary data	It discussed the social support networks, concerns and unmet needs among older trans and gender non-conforming populations during UK's COVID-19 lockdown.
79	Psychological impact of domestic violence on women in India due to COVID-19	Victims of domestic violence	Tripathi, P.	2022	India	Qualitative study	Secondary data	The study claimed that the economic instability induced by the COVID-19 pandemic worsened the psychological abuse against women in India.
80	The Effect of Prolonged Lockdown Due to COVID-19 on Greek Demented Patients of Different Stages and on Their Caregivers	People with disabilities	Tsapanou, A.	2021	Greece	Cross-sectional study (Survey and statistical analysis)	Primary data	It investigated the effect of the COVID-19 induced prolonged isolation on the physical and psychological burden of the population with dementia and their caregivers.
81	A quasi-experimental study of parent and child well-being in families of color in the context of COVID-19 related school closure	Student/Children	Ursache, A.	2022	India	Quasi-experimental study	Primary data	It explored how COVID-19 related school closure affected child and parent mental health and sleep in families of color.
82	Intimate partner violence against women during the COVID-19 lockdown in Spain	Victims of domestic violence	Vives-cases, C.	2021	Spain	Descriptive ecological study	Secondary data	It examined the evolution of intimate partner violence against women before and atfer the COVID-19 lockdown in Spain through analysing temporal and geographical changes of different indicators.
83	Longitudinal changes in mental health following the COVID-19 lockdown: Results from the Survey of Health, Ageing and Retirement in Europe	Older people	Wester, C. T.	2022	Europe	Longitudinal study	Secondary data	The study examined the longitudinal mental health changes among people aged 50 and above before and during the COVID-19 lockdown.
84	Psychosocial status of older adults aged 65 years and over during lockdown in Turkey and their perspectives on the outbreak	Older people	Yıldırım, H.	2022	Turkey	Qualitative study (interviews and thematic analysis)	Primary data	The study explored psychosocial status of adults aged 65 and over during COVID-19 lockdown in Turkey.

No.	Title	Population of interest	First author	Year	Country	Study design/type	Data source	Brief description
85	Mental and Social Health of Children and Adolescents With Pre-existing Mental or Somatic Problems During the COVID-19 Pandemic Lockdown	Student/Children	Zijlmans, J.	2021	Netherlands	Cross-sectional study (Survey and statistical analysis)	Primary data	The study explored the mental and social health conditions among children and adolescents who had pre-existing mental or somatic issues amid the COVID-19 lockdown period.
86	Impact of COVID-19 lockdowns on adolescent pregnancy and school dropout among secondary schoolgirls in Kenya	Student/Children	Zulaika, G.	2022	Kenya	Causal-comparative research design	Primary data	The study stated that the COVID-19 and its school closure measures deleteriously affected the sexual and reproductive health and school dropout among adolescent girls.

Supplementary Table 3. Distribution of studies across countries and vulnerable populations

Vulnerable population	Number of studies	Distribution across countries	Vulnerable population	Number of studies	Distribution across countries
Older people	37	Not specific (1 study): [1]<br>Global (1 study): [2]<br>EU countries (1 study): [3]<br>Europe (4 studies): [4,5,6,7]<br>France (1 study): [8]<br>Italy (3 studies): [9,10,11]<br>Spain (4 studies): [12,13,14,15]<br>Germany (1 study): [16]<br>Turkey (1 study): [17]<br>Finland (1 study): [18]<br>Sweden (1 study): [19]<br>South Korea (1 study): [20]<br>Brazil (1 study): [21]<br>China (2 studies): [22,23]<br>India (3 studies): [24–26]<br>US (4 studies): [27–29,30]<br>UK (3 studies): [31,32,33]<br>Canada (1 study): [34]<br>Centro American countries (1 study):[35]<br>sub-Saharan Africa (1 study): [36]<br>Australia (1 study): [37]	Children/students	96	Not specific (7 studies): [38–44]<br>Global (9 studies): [45–49, 50,51,52]<br>9 countries in Europe (1 study): [53]<br>Low- and lower-middle-income countries (1 study): [54]<br>Germany, Austria, Switzerland (1 study): [55]<br>Germany (1 study): [56]<br>UK (11 studies): [57–63,64,65,66,67]<br>US (19 studies): [68–85,86]<br>Brazil (2 studies): [87,88]<br>Finland and Sweden (1 study): [89]<br>Italy (3 studies): [90,91,92]<br>Norway (2 studies): [93,94-95]<br>Denmark (1 study): [96]<br>Belgium (1 study): [97]<br>Spain (1 study): [98]<br>Turkey (1 study): [99]<br>Portugal (1 study): [100]<br>Egypt (1 study): [101]<br>France (2 studies): [102,103]<br>Netherlands (2 studies): [104,105]<br>Australia (3 studies): [106,107,108]<br>Canada (1 study): [109]<br>China (1 study): [110]<br>India (11 studies): [111–116,117,118,119,120,121]<br>Bangladesh (1 study): [122]<br>Japan (3 studies): [123–125]<br>Nepal (1 study): [126]<br>Pakistan (1 study): [127]<br>South Africa (3 studies): [128–130]<br>Namibia (1 study): [131]<br>Kenya (1 study): [132]<br>Jordan (1 study): [133]
Low-income populations	58	Not specific (3 studies): [134–136]<br>Global (2 studies): [45,137]<br>UK (4 studies): [57,138,139,140]<br>US (11 studies): [141–151]<br>India (11 studies): [26,116,152–157,158,159,160]<br>Chile (1 study): [161]<br>Bangladesh (1 study): [162]<br>Brazil (1 study): [163]<br>Canada (2 studies): [109,164]<br>China (2 studies): [165,166]<br>Philippines (1 study): [167]<br>Italy (1 study): [168]<br>Mexico (1 study): [169]<br>Nigeria, Rwanda, South Africa, and Uganda (1 study): [170]<br>Kenya (1 study): [171]<br>Namibia (1 study): [131]<br>Kenya, South Africa (1 study): [172]<br>Africa (1 study): [173]<br>Kenya, South Africa, Nigeria (1 study): [174]<br>South Africa (6 studies): [129,130,175–178]<br>Nigeria (2 studies): [179,180]<br>Uganda (1 study): [181]<br>Zimbabwe (1 study): [182]<br>New Zealand (1 study): [183]	Migrant workers	14	Not specific (1 study): [135]<br>India (5 studies): [25,116,184,185,186]<br>South Africa (1 study): [128]<br>Malta (1 study): [187]<br>Singapore (2 studies): [188,189]<br>Bangladesh (1 study): [190]<br>France (1 study): [191]<br>UK (1 study): [192]<br>Mexico (1 study): [193]
People in prison	7	Global (1 study): [194]<br>Africa (1 study): [173]<br>Portugal (1 study): [195]<br>Sweden (1 study): [196]<br>UK (1 study): [197]<br>US (2 study): [198, 199]	People with disabilities	15	Global (1 study): [147]<br>Australia (1 study): [107]<br>India (1 study): [156]<br>Italy (4 studies): [90,200,201,200]<br>Spain (1 study): [202]<br>US (3 studies): [85,203,204]

Vulnerable population	Number of studies	Distribution across countries	Vulnerable population	Numbe r of studies	Distribution across countries
					Belgium (1 study): [97]<br>UK (1 study): [205]<br>Greece (1 study): [206]<br>Romania (1 study): [207]
Sex workers	9	Not specific (1 study): [208]<br>Africa (1 study): [209]<br>India (2 studies): [210,211]<br>Kenya (2 studies): [212,213]<br>Thailand (1 study): [214]<br>Singapore (1 study): [215]<br>Nigeria (1 study): [216]	Victims of domestic violence	16	Not specific (1 study): [217]<br>Global (1 study): [218]<br>Bangladesh (3 studies): [219,220,221]<br>India (2 studies): [26, 222]<br>UK (2 studies): [223,224]<br>Spain (1 study): [225]<br>US (2 studies): [74,226]<br>South Africa (1 study): [227]<br>Guatemala (1 study): [228]<br>Democratic Republic of Congo (1 study): [229]<br>Norway (1 study):[230]
Refugees	14	Not specific (4 studies): [231–234]<br>Africa (1 study): [173]<br>Germany (1 study): [235]<br>Greece (2 studies): [236,237]<br>South Africa (1 study): [238]<br>UK (1 study): [239]<br>US (2 studies): [240,241]<br>Jordan (1 study): [242]<br>Italy (1 study): [243]	Ethnic minority	10	India (1 study): [157]<br>UK (5 studies): [138,244–246,247]<br>US (4 studies): [70,147,248,249]
People from sexual and gender minorities	11	Not specific (2 studies): [250,251]<br>India (1 study): [252]<br>UK (2 studies): [253,33]<br>US (5 studies): [254–258]<br>Brazil (1 study): [259]	General vulnerable	5	Studies discussing more than three vulnerable groups or talking about vulnerable populations in general without specifying detailed population groups.<br><br>UK (1 study): [260]<br>South Korea (1 study): [261]<br>Austria (2 studies): [262,263]<br>Netherlands (1 study): [264]
Total	265				

Note: 1) “Not specific” indicates studies not mentioning specific geographic study scopes.

Supplementary Table 4. Summarized characteristics of included studies

Indicator	Details	Number of studies	Percent (% out of 179 studies)
Country of origin	Single country	218	82.26
	• US	48	18.11
	• India	30	11.32
	• UK	27	10.19
	Multiple countries/Global	28	10.57
	Not specific	19	7.17
Population of interest	Older people	37	13.96
	Children/students	96	36.23
	Low-income populations	58	21.89
	Migrant workers	14	5.28
	People in prison	7	2.64
	People with disabilities	15	5.66
	Sex workers	9	3.40
	Victims of domestic violence	16	6.04
	Refugees	14	5.28
	Ethnic minority	10	3.77
	People from sexual and gender minorities	11	4.15

Supplementary Table 5. Data for Fig. 2a global map

Country	Continent	Older people	Children/students	Low income populations	Migrant workers	People in prison	People with disabilities	Sex workers	Victims of domestic violence	Refugee	Ethnic minority	People from sexual and gender minorities
Congo DRC	Africa	0	0	0	0	0	0	0	1	0	0	0
Egypt	Africa	0	1	0	0	0	0	0	0	0	0	0
Kenya	Africa	0	1	3	0	0	0	2	0	0	0	0
Namibia	Africa	0	1	1	0	0	0	0	0	0	0	0
Nigeria	Africa	0	0	6	0	0	0	1	0	0	0	0
Rwanda	Africa	0	0	1	0	0	0	0	0	0	0	0
South Africa	Africa	0	3	9	1	0	0	0	1	1	0	0
Uganda	Africa	0	0	2	0	0	0	0	0	0	0	0
Zimbabwe	Africa	0	0	1	0	0	0	0	0	0	0	0
Bangladesh	Asia	0	1	1	1	0	0	0	3	0	0	0
China	Asia	2	1	2	0	0	0	0	0	0	0	0
India	Asia	3	11	11	5	0	1	2	2	0	1	1
Japan	Asia	0	3	0	0	0	0	0	0	0	0	0
Jordan	Asia	0	1	0	0	0	0	0	0	1	0	0
Nepal	Asia	0	1	0	0	0	0	0	0	0	0	0
Pakistan	Asia	0	1	0	0	0	0	0	0	0	0	0
Philippines	Asia	0	0	1	0	0	0	0	0	0	0	0
Singapore	Asia	0	0	0	2	0	0	1	0	0	0	0
South Korea	Asia	1	0	0	0	0	0	0	0	0	0	0
Thailand	Asia	0	0	0	0	0	0	1	0	0	0	0
Australia	Australia	1	3	0	0	0	1	0	0	0	0	0
Austria	Europe	0	1	0	0	0	0	0	0	0	0	0
Belgium	Europe	0	1	0	0	0	1	0	0	0	0	0
Denmark	Europe	0	1	0	0	0	0	0	0	0	0	0
Finland	Europe	1	1	0	0	0	0	0	0	0	0	0
France	Europe	1	3	0	1	0	0	0	0	0	0	0
Germany	Europe	1	1	0	0	0	0	0	0	1	0	0
Greece	Europe	0	0	0	0	0	1	0	0	2	0	0
Ireland	Europe	0	1	0	0	0	0	0	0	0	0	0
Italy	Europe	3	4	1	0	0	4	0	0	1	0	0
Luxembourg	Europe	0	1	0	0	0	0	0	0	0	0	0
Malta	Europe	0	1	0	1	0	0	0	0	0	0	0
Netherlands	Europe	0	2	0	0	0	0	0	0	0	0	0
Norway	Europe	0	3	0	0	0	0	0	1	0	0	0
Portugal	Europe	0	2	0	0	1	0	0	0	0	0	0
Romania	Europe	0	0	0	0	0	1	0	0	0	0	0

San Marino	Europe	0	1	0	0	0	0	0	0	0	0	0
Spain	Europe	4	2	0	0	0	1	0	1	0	0	0
Sweden	Europe	1	1	0	0	1	0	0	0	0	0	0
Switzerland	Europe	0	1	0	0	0	0	0	0	0	0	0
Turkey	Europe	1	1	0	0	0	0	0	0	0	0	0
United Kingdom	Europe	3	12	4	1	1	1	0	2	1	5	2
Canada	North America	1	1	2	0	0	0	0	0	0	0	0
Guatemala	North America	0	0	0	0	0	0	0	1	0	0	0
Mexico	North America	0	0	1	1	0	0	0	0	0	0	0
United States	North America	4	19	11	0	2	3	0	2	2	4	5
New Zealand	Oceania	0	0	1	0	0	0	0	0	0	0	0
Brazil	South America	1	2	1	0	0	0	0	0	0	0	1
Chile	South America	0	0	1	0	0	0	0	0	0	0	0

Supplementary Table 6. Data for Fig. 2b data distribution around Europe

Country	Continent	Older people	Children/students	Low income populations	Migrant workers	People in prison	People with disabilities	Sex workers	Victims of domestic violence	Refugee	Ethnic minority	People from sexual and gender minorities
Austria	Europe	0	1	0	0	0	0	0	0	0	0	0
Belgium	Europe	0	1	0	0	0	1	0	0	0	0	0
Denmark	Europe	0	1	0	0	0	0	0	0	0	0	0
Finland	Europe	1	1	0	0	0	0	0	0	0	0	0
France	Europe	1	3	0	1	0	0	0	0	0	0	0
Germany	Europe	1	1	0	0	0	0	0	0	1	0	0
Greece	Europe	0	0	0	0	0	1	0	0	2	0	0
Ireland	Europe	0	1	0	0	0	0	0	0	0	0	0
Italy	Europe	3	4	1	0	0	4	0	0	1	0	0
Luxembourg	Europe	0	1	0	0	0	0	0	0	0	0	0
Malta	Europe	0	1	0	1	0	0	0	0	0	0	0
Netherlands	Europe	0	2	0	0	0	0	0	0	0	0	0
Norway	Europe	0	3	0	0	0	0	0	1	0	0	0
Portugal	Europe	0	2	0	0	1	0	0	0	0	0	0
Romania	Europe	0	0	0	0	0	1	0	0	0	0	0

San Marino	Europe	0	1	0	0	0	0	0	0	0	0	0
Spain	Europe	4	2	0	0	0	1	0	1	0	0	0
Sweden	Europe	1	1	0	0	1	0	0	0	0	0	0
Switzerland	Europe	0	1	0	0	0	0	0	0	0	0	0
Turkey	Europe	1	1	0	0	0	0	0	0	0	0	0
United Kingdom	Europe	3	12	4	1	1	1	0	2	1	5	2

Supplementary Table 7. Data for Fig. 2c data distribution around Asia

Country	Continent	Older people	Children/students	Low income populations	Migrant workers	People in prison	People with disabilities	Sex workers	Victims of domestic violence	Refugee	Ethnic minority	People from sexual and gender minorities
Bangladesh	Asia	0	1	1	1	0	0	0	3	0	0	0
China	Asia	2	1	2	0	0	0	0	0	0	0	0
India	Asia	3	11	11	5	0	1	2	2	0	1	1
Japan	Asia	0	3	0	0	0	0	0	0	0	0	0
Jordan	Asia	0	1	0	0	0	0	0	0	1	0	0
Nepal	Asia	0	1	0	0	0	0	0	0	0	0	0
Pakistan	Asia	0	1	0	0	0	0	0	0	0	0	0
Philippines	Asia	0	0	1	0	0	0	0	0	0	0	0
Singapore	Asia	0	0	0	2	0	0	1	0	0	0	0
South Korea	Asia	1	0	0	0	0	0	0	0	0	0	0
Thailand	Asia	0	0	0	0	0	0	1	0	0	0	0

Supplementary Text

A1. Scope of physical distancing measures

Physical distancing measures range from lockdowns, school closures, to restrictions on social gatherings at homes and public places (see Supplementary Fig. 1). For instance, most countries in the world have, at various stages of the pandemic, implemented strong policy measures like lockdowns or stay-at-home/shelter-in-place orders to curb the COVID-19 transmission; these include China [265], India [153], US [266], UK [246], Italy [267], and Germany [16].

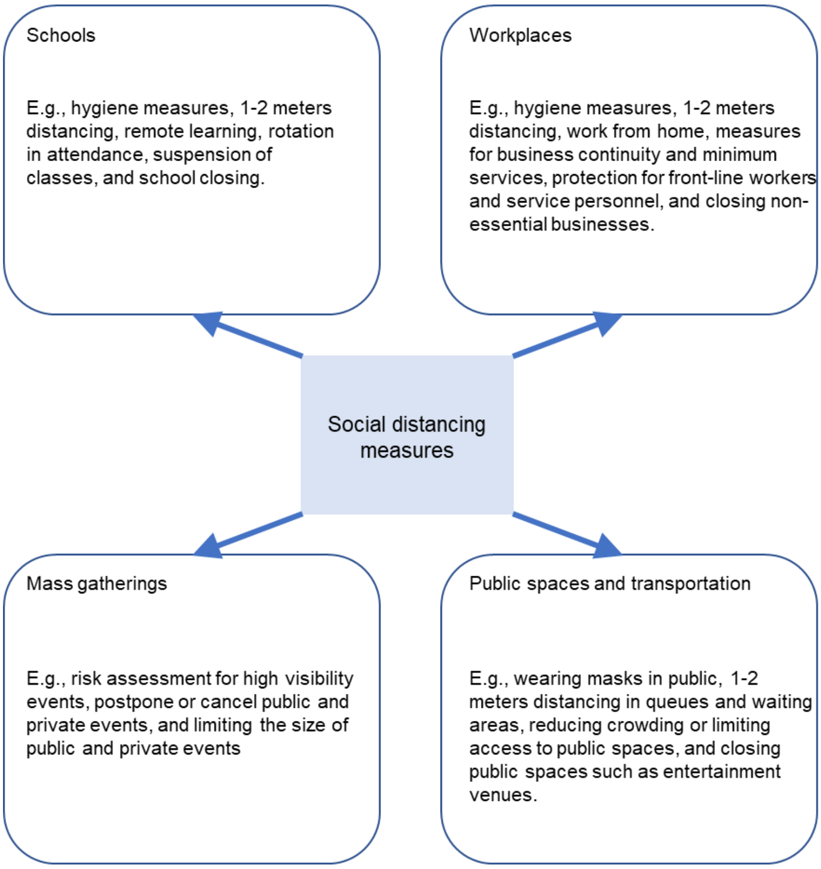

Supplementary Fig. 1. Physical distancing measures listed by World Health Organization (WHO). Based on WHO's Overview of public health and social measures in the context of COVID-19 (Interim guidance) [268]

A2. Classification of vulnerability

Following the classification of vulnerability from the National Bioethics Advisory Committee (NBAC) which necessitates research involving human participants to identify the vulnerability in human subjects [269,270], we classify vulnerability as 1) cognitive or communicative vulnerability due to insufficient ability to comprehend information and make decisions; 2) institutional or deferential vulnerability due to the circumstances where people's decisions/behaviours are subject to the authority of others; 3) health vulnerability, such as long-term sick or disabled persons; 4) economic vulnerability when people are disadvantaged in income, housing and other goods and services; and, 5) social vulnerability when a person belongs to undervalued social groups. Sources of these vulnerability[17] may either stem from intrinsic characteristics, such as lack of cognitive capacity (e.g., children); or induced by external shocks such as unemployment, impoverishment, or incarceration.

Vulnerability is sensitive to specific contexts [270]. For example, older people are vulnerable to COVID-19 as they face higher risks of developing severe illnesses and complications once they contract with the COVID-19 viruses [271,272], even though they may not be considered vulnerable in the pre-pandemic contexts [273]. In the pandemic context [274], sources of health vulnerability include "the likelihood of exposure, of contracting the disease if exposed, and of timely and effective response or treatment" [274(p.234)].

Social and economic vulnerabilities may exacerbate health vulnerabilities, or contribute to the development of health vulnerabilities [275,276]. Mikolai et al. [277] states that household-level vulnerabilities can be examined by digital and connectivity, housing conditions, employment conditions, financial conditions and health conditions. Social, economic, and health conditions can intersect and co-produce vulnerability [278]. For instance, low-income populations often face poor housing condition; overcrowding and poorly ventilated housing increases health risk; as well as poor health jeopardizing their employment and income [278]. OECD[279] also considers vulnerable groups not limited to those in poor physical or mental health, but also those in disadvantaged social and economic conditions, such as those in disadvantaged employment or financial conditions and those facing poor housing conditions.

A public health crisis such as COVID-19 would hit the vulnerable groups the hardest, and physical distancing measures could heavily impact them from financial and psychological perspectives [280]. The World Bank[281] also claims that the most vulnerable populations often lack the essential services to prevent a COVID-19 outbreak. For the sake of social justice and health equity, the state and public health agencies are obligated to protect populations that are especially vulnerable because of their disadvantaged status [280].

A3. Data and methods

We conducted the searches twice respectively to search for relevant publications, with the first search happening from March 2021 to April 2021, and the updated search happening in June 2022. The search strategy for updated search is generally consistent with the first search, with additional search terms of vulnerable populations to enhance search precision.

Supplementary Table 8. Key terms included in updated search

Concepts	Key terms in search strings
Covid-19	"ncov" OR "2019 ncov" OR "Covid-19" OR "Covid19" OR "Covid-2019" OR "Covid2019" OR "sars-cov-2" OR "sars cov-2" OR "sarscov2" OR "sarscov-2" OR "sars-coronavirus-2" OR "sars corona virus" OR "sars-like coronavirus" OR "novel coronavirus" OR "novel corona virus" OR "Covid*" OR "coronavirus 2" OR "coronavirus infection*" OR "coronavirus disease" OR "corona virus disease" OR "new coronavirus" OR "new corona virus" OR "new coronaviruses" OR "novel coronaviruses" OR "severe acute respiratory syndrome coronavirus 2" OR "coronavirus" OR "sars-cov"
Social distancing	"social distancing" OR "social isolation" OR "physical distancing" OR "physical distance" OR "safe distancing" OR "lockdown" OR "lock down" OR "quarantine" OR "stay-at-home" OR "stay at home" OR "self isolation" OR "self-isolation" OR "remote work" OR "school closure" OR "workplace closure"
Policy measure	"act" OR "design" OR "govern*" OR "intervention" OR "law" OR "legislation" OR "politics" OR "regulation" OR "policy" OR "policies" OR "policy measure" OR "policy instrument" OR "policy mix" OR "policy bundle" OR "policy package"
Vulnerable groups	"aged" OR "long-term care" OR "long term care" OR "long-term residential care" OR "long-term senior care" OR "senior*" OR "elder*" OR "older adult*" OR "nursing home" OR "nursing care" OR "child" OR "schoolchild*" OR "school age*" OR "preschool*" OR "kid*" OR "adoles*" OR "teen*" OR "minor" OR "minors" OR "nursery school*" OR "kindergar*" OR "primary school*" OR "secondary school*" OR "elementary school*" OR "high school*" OR "highschool*" OR "student*" OR "poverty" OR "poor" OR "impoverish*" OR "lower-income" OR "lower income" OR "destitute*" OR "destitution" OR "low income" OR "low-income" OR "ghetto*" OR "slum*" OR "transient worker*" OR "migrant*" OR "migrant worker*" OR "foreign worker*" OR "domestic helper*" OR "prison*" OR "jail*" OR "prisoner*" OR "inmate*" OR "disabilit*" OR "disabled" OR "handicap*" OR "physically challenged" OR "amputee*" OR "mentally ill" OR "hearing impair*" OR "hearing disabled" OR "deaf" OR "mentally disabled" OR "mentally handicapped" OR "intellectually disabled" OR "intellectual disability*" OR "mentally retarded" OR "mental deficienc*" OR "visually impair*" OR "blind" OR "learning disabilit*" OR "dyslexia" OR "sex worker*" OR "prostitut*" OR "brothels" OR (("domestic" OR "child*" OR "elder" OR "spouse" OR "spousal" OR "partner" OR "wife" OR "women" OR "woman" OR "family") AND ("abuse" OR "violence" OR "violent")) OR "refugee*" OR "asylum seeker*" OR "displaced person*" OR "minority group*" OR "ethnic minorit*" OR "racial minorit*" OR "marginalised group" OR "marginalized group" OR "sexual minorit*" OR "gender minorit*" OR "intersex person*" OR "transgender person*" OR "transsexual*" OR "transgender*" OR "LGBT" OR "LGBTQ*" OR "lesbigay" OR "non-heterosexual" OR "lesbian*" OR "bisexual" OR "queer*" OR "homosexual"

Data extraction mainly focus on 1) study characteristics such as main research aims and findings, research methodology, vulnerable population examined setting (i.e., country/region/city); 2) physical distancing measures and their impacts on the vulnerable populations, 3) Policy measures designed to ringfence vulnerable populations. Relevant narratives in the included articles were extracted to a spreadsheet.

A4. Results

1) Older people, vulnerability & physical distancing

The older people, especially those with chronic diseases, face health vulnerability as they have high risks of developing severe COVID-19 symptoms once contracted. They are also vulnerable to developing mental health problems such as anxiety and depression when isolated at home [26,31].

In most countries, despite physical distancing measures were neutrally applied to all populations, they may harbour negative impacts on the older people. Physical distancing measures were found to be effective in mitigating COVID-19 infection and deaths among the older people [22,27]. Thus, in Brazil and France, there were discussions on whether governments should continue to isolate the older people while lifting the physical distancing measures for other age groups [8,21]. However, physical distancing policy measures, particularly isolation at homes, were reported to have negatively affected the older people's mental health such as prolonged loneliness due to limited social activities and reduced close contact with family members in the UK, the US and India [26,28,29,31], and resulted in decreased utilisation of hospital services (i.e., decreased physician consultations and hospital admissions for diseases other than the COVID-19) in Germany [16]. These impacts further predisposed the older people to greater risks of cardiovascular, autoimmune, and neurocognitive diseases [29]. Some older people were also prone to economic vulnerability which may exacerbate their health vulnerability due to decreased access to essential living needs or medical services. For instance, a study on India reported that the income loss amount was higher among the older migrant workers than other age groups in sectors that remained non-operational during the lockdown [25].

Rather than lockdowns, other more cost-effective policy measures were analysed in literature. As an example, distributing hygiene kits is considered effective in preventing COVID-19 spread while mitigating unintended policy consequences, especially in low- and middle- income countries [36]. To mitigate risks of COVID-19 outbreaks within nursing homes or LTC facilities, early screening, detection and contact tracing were found to be effective [20]. For instance, the Korean government conducted a nationwide surveillance of 1470 LTC facilities from February to March 2020 to check if they applied restriction to visitors, and whether identification and isolation of patients with COVID-19

symptoms, and quarantine of employees who had recently travelled to high-risk countries/regions were strictly adhered [20]. Distribution of tools for detection and remote monitoring of cases can also facilitate the COVID-19 control in LTCs [12,34].

To address mental health issues faced by the older people when socially isolated, the use of technology was considered useful [1,12,24,34]. Information and communication technologies (ICT) help the older people to maintain social connections with their loved ones and attend virtual recreational activities (e.g., playing games) to mitigate the loneliness and maintain their wellbeing when they were isolated at home or long-term care (LTC) facilities [24]. For instance, in New Brunswick in Canada, the government provided one iPad for every 10 residents residing in LTC facilities to enable them to connect with their family members virtually [34]. Besides, the deployment of student volunteers was found to reduce poor mental health outcomes among the older population [34]. Furthermore, the use of technologies such as online shopping and digital government/banking services make the older people less dependent on other people in India [24].

2) Children/students, vulnerability & physical distancing

Children are subject to cognitive or communicative vulnerability at young ages. Children are dependent persons (subject to economic vulnerability) relying on their parents/guardians for living needs and financial support [282,283]. Poor economic conditions, such as lack of access to schooling, health and social services, and inadequate clothing, contribute to the vulnerability of children [283].

Almost all included studies on children/students discussing school closing and remote learning. Many countries and states implemented school closures in different periods to reduce COVID-19 transmission in school settings [77]. Nonetheless, there were debates on the extent to which school closures were effective in breaking the transmission and the necessity of school reopening to facilitate effective learning [40,48,53,63,71,88,89,93,123]. Decisions on school closures or reopening should consider the severity of COVID-19 infections in the community and the ability of schools to enforce physical distancing as well as to detect and trace suspected cases on campus [53,63].

As shown in many countries, school reopening did not necessarily result in transmisión when health and safety protocols were adhered to. These include teaching children hygiene measures (e.g., washing hands), mask wearing mandates, 1- to 2- metre distancing, screening at entry, grouping students and reducing class sizes, limiting after-school activities, moving to outdoor classrooms, testing and tracing suspected cases, quarantine of confirmed cases, suggesting parents not to congregate outside the campus gates, staggered drop off and pick up times, air circulation enhancement, shortening school hours, and offering options of in-person, remote or hybrid learnings, disinfecting places [39,46,48,58–62,68,72,73,75,76,79,80,82,84,100,110]. Krishnaratne et al.[46] summarized that there were three types of measures in school settings to reduce COVID-19 spread while keeping schools open, including organisational measures (e.g., staggered drop off and pick up times), structural/environmental measures (air circulation), and surveillance and quarantine measures (e.g., contact tracing).

Studies in India, Italy, Japan, Nepal, Pakistan, Spain, South Africa, Switzerland, the US, and Turkey had reported that school closures left negative impacts on mental health of children, causing issues such as anxiety, loneliness, depression, irregular sleep pattern, aggression, disruptive and rule breaking behavioural problems, and addiction with internet, smartphones and laptops [44,55,83,91,98,99,111–116,125–127,129]. To address children's mental health issues, parents were suggested to communicate constructively with their children about the current COVID-19 pandemic situations, in accordance with their maturity level and ability to understand the crisis [42,47]. In India, Pakistan, South Africa and the US, mental health support programs were established to help children with mental health issues when isolated at home [83,111,127,129]. And schools and families must pay attention to assessment and identification of children's mental health needs or issues [111,115].

Other unintended detrimental impacts of school closures include negative impacts on educational outcomes (differing across learning courses), physical health of children (e.g., weight gain) [43,124]. School closures caused widespread concerns about children's suboptimal learning at home [38]. Remote learning (also known as distance learning) during school closures exerted pressures on teachers' technical capacities to operate digital teaching and often increased their working time as they learned to navigate the new virtual space to follow up with students' learning progress and feedbacks [87].

Research had shown that the teachers' capacity in operating digital teaching is positively associated with the students' learning task control [55].

Across the world, remote learning sparked concerns about the widening of the learning gaps between students from different socioeconomic statuses. A virtual learning environment caused inevitable disruptions. Children from low-income families were more significantly affected by disruptions in learning and deprived of services and supports that they could have received in schools [45,57,109,129,131]. They were likely to lack access to the internet and other digital resources such as computers, and faced issues such as lack of regular meals and parental support, home environment not conducive for learning, and high risk of exposure to virus in crowding housing conditions [57,90,109,130,131]. Besides, the loss of working hours of parents due to childcare added to household financial difficulties [109], and countries such as South Korea developed childcare programs for parents who needed help with childcare due to school closures [41].

To enhance remote learning, especially to support vulnerable students from low-income families, solutions ranged from circular printed materials for learning from home [55], applying television broadcast related to courses or other media [99], identifying children's needs and offering target instructions [69,78,81], and offering support of laptops, helplines, free-of-charge data sim cards, and food programs [70,106]. In South Africa, civic society organisations stepped up to offer classrooms for students to maintain remote learning [128].

Many governments also established funds or fiscal stimulus to meet educational needs during COVID-19 [45,69,78]. For instance, in the US, the federal government approved a $13.5 billion funding for schools via a federal stimulus package – the Coronavirus Aid, Relief, and Economic Security (CARES) Act. CARES Act utilised the Governor's Education Relief Fund and the Elementary and Secondary School Emergency Relief Fund to allocate funds for K-12 education (i.e., from kindergarten to 12th grade). In addition to these one-time funds, governments attempted to secure short-term and long-term funds for school education to improve their technical equipment and personnel resources. In Italy, after the "Cura Italia" (Care Italy) Decree-Law (no.18/2020) was implemented, 85 million euros was allocated to schools to enhance remote learning [90]: In particular, more than half of the funds were

earmarked for procurement of digital devices which can be given to students from low-income families on loan for free use, while the rest being utilised to build e-learning platforms and capacity building for the staff. In Australia, individualized funding schemes such as the National Disability Insurance Scheme (NDIS) offered financial support for children and their families to access services or technologies that can facilitate remote learning for children [107]. Health vulnerability, which include all forms of disabilities (physically, mentally, intellectually, or sensorially), makes it harder for children to adjust to remote learning, as documented in Australia and the US [47,85,107]. In addition to digital and connectivity conditions, students with disabilities may not have essential technologies to facilitate their learning at home which they can access easily in school, and this situation is most notably reported among the braille readers [85]. In the United States, school districts plan and deliver learning supports for students with disabilities, but it was proven to be challenging due to variations in the types of services needed and the length of specialized instruction required for each child [85].

3) Low-income populations, vulnerability & physical distancing

For the low-income population, economic vulnerability is the primary vulnerability. The World Bank defines living under US$1.90 per day as extreme poverty and uses the poverty line to measure global extreme poverty [284,285]. In addition, the World Bank uses the US$3.20-per-day and US$5.50-per-day poverty lines to measure poverty in lower-middle-income and upper-middle-income countries respectively [284,285]. The COVID-19 pandemic crisis caused sharp decline in economic activities around the world [286]. As estimated, the pandemic has pushed approximately 119 to124 million population into extreme poverty in 2020 [287]. The COVID-19-induced new poor are likely to be people living in congested urban settings and working in sectors such as informal services and manufacturing that are most affected by lockdowns and physical distancing restrictions [288].

As the definition suggests, low-income populations face adverse financial conditions. They may also face less favorable employment conditions, such as temporary contract, part-time employment, self-employment, and low-income informal sector workers. Low income levels also correlate with poor health [146,289], and poor housing condition or even homelessness [146]. As low-income population typically

have little savings, job and income loss can further worsen the financial situation, causing other health risks, such as food insecurity [148].

The challenge of low-income populations to adhere strictly to various physical distancing measures were consistent among studies. This is due to congregate living spaces, dearth of sanitation facilities, unable to work from home (e.g., manufacturing and processing industries), lack of access to job protections or paid leave, food shortage and insecurity, worse financial conditions and living off savings, no regular access to basic hygiene, and lack of resources and infrastructure to do testing, isolation and contract tracing [26,116,130,137,141,142,144–153,155,157,163,167–169,171,173,175,178]. Lockdowns were reported to be less effective in containing and reducing new COVID-19 cases in low-income countries or communities [134,142,161,179]. Besides, social vulnerability intersects with economic vulnerability of the low-income populations, contributing to their health vulnerability. For instance, in slums or informal settlements, residents were exposed to poor living conditions, a dearth of sanitation facilities, and a lack of clean drinking water; the high population density in slums rendered 1-meter or 2-meter distancing impractical [26,130,154,155,171,172,179]. These have negative repercussions for the government in terms of tracing, testing and treating the COVID-19 disease. As many low-income populations are daily bread winners in informal sectors such as street vendors or daily wage workers in essential sectors that remain operating during COVID-19, the options for them to work remotely is limited [140,149]. Physical distancing measures did not significantly reduce work-related trips for the low-income group, making them bear a disproportionate risk of exposure to virus [149]. They also face barriers to access healthcare due to income loss and reduced employer-sponsored health care [151,178]. Access to government aid or relief packages offered by governments is limited [135,169,177,179]. Furthermore, students from low-income background face various challenges to adapt to school closure and remote learning as discussed above [45,57,109,129,131]. Mental distress of the low-income group during COVID-19 was also discussed in literature [130,156,177].

As for the low-income populations in rural areas, they may have insufficient personal hygiene and living materials, weak awareness on infection prevention and control, decreased non-farm income and limited sources of income, lack of food or monetary support, lack of access to basic needs such as

groceries, lack of access to clean water and health items, and lack of access to smartphone and internet, lack of access to health care, or lack of access to COVID-19 information and guidelines [141,156,166,177,181].

To control COVID-19 spread among low-income communities, physical distancing measures should be context specific [167]. Intensive screening, testing, contract tracing and isolation of infected cases were useful to keep cases low [162,173,174]. For instance, a large-scale slum in India, Dharavi, flattened the curve quickly through screening, contract tracing and quarantine measures [154]. Other measures such as offering affordable masks, clean water, sanitizer, and soaps, and decongestion are also necessary [172–174]. A program in Canada offered mobile devices for the low-income group, which enabled participants to use mobile phone to access medical specialists and attend telemedicine appointments [164]. For rural populations, case detection and surveillance capacity must be improved, and basic amenities such as internet access, drinking water, public transport plans and electricity need to be made available [165,166].

Greater economic support has been called for to assist the low-income population [45,138,149,155,157,168,176]. For instance, government transfer payments targeted the low-income were found to be helpful [157,176]. NGOs worked with governments to offer food and other necessities (e.g., fuel) in countries such as India, Chile, and Nigeria [137,154,157,179]. Access to unemployment insurance, paid medical leave and reemployment services were also strengthened for the low-income [139,148,149]. For instance, the UK government implemented the Coronavirus Job Retention Scheme to support low paid young workers in the form of a furlough payment that provides wages for workers on a temporary layoff or involuntary leave due to COVID-19 [139].

A more effective communication channel between the low-income population and governments can be established [149]. Community-led efforts by NGOs were instrumental to aid the low-income population during the pandemic. Their efforts ranged from offering food, distributing COVID-19 information and education through social media or SMS, facilitating contract tracing and quarantine, delivering materials groceries and other essential items to patients in quarantine, to establishing longitudinal clinical and social support for them through the involvement of community health workers and public health specialists [136,143,154,170].

4) Migrant workers, vulnerability & physical distancing

Migrant workers are defined as "a person who is to be engaged, is engaged or has been engaged in a remunerated activity in a state of which he or she is not a national" [290]. They move away from their place of usual residence across the international border to a different country or simply to a different place within a country [291]. The latter group are called internal migrant workers [291]. Migrant workers are mainly exposed to the economic vulnerability caused by adverse employment conditions, such as precarious recruitment processes, absence of accurate information on terms and conditions of employment contracts, and lack of labour law coverage in the destination state [292]. They may also face mental health vulnerability [184] due to social and cultural isolations at the destination state.

Due to lockdowns/stay-at-home orders implemented to control COVID-19, migrant workers (inter-state or inter-country) faced a variety of challenges which include job losses and incurred income losses [25], and food insecurity [25,185], high exposure risks in crowded dormitories living places [188] [187], and limited access to primary healthcare and COVID-19 treatment in destination countries [135,187]. Migrant workers tend not to qualify for many COVID-19 related social benefits (e.g., food rations) provided by the destination country and were likely to be repatriated to their home countries [128]. Migrant workers also suffered significant mental stress and loneliness due to financial instability, isolation of migrant worker dormitories, language and cultural barriers, inability to send remittances to families, and barriers to return home to see their family members [116,184,187,188].

In India, inter-state migrant workers faced various challenges during the lockdown [25,116,184,185]. The migrant workers faced travel restrictions and attempted to walk back to their home villages from the cities but were arrested in various inter-state borders for violating the lockdown mandate, which started on 24th March 2020 [25]. Meanwhile, the consequent shortage of labour forces in the urban areas forced the available migrant workers to work for longer hours than usual for business owner to keep up with the production [184], leading to health risks resulted from sleep deprivation and fatigue. To help the migrant workers return home, the Indian Government's order on 29th April 2020 announced the resumption of transport facilities such as buses and trains for migrant workers, on the condition that both the destination state government and inception state government could make a joint request to the

central government to apply for the resumption of transportation [185]. Nonetheless, the order engendered chaos due to ineffective communications between Indian states and Union territories [185]. During the lockdown, the Indian central government and some state governments suggested employers to continue paying full wages and salaries to employees. These advisories were met with resistance because many small businesses could not afford to adhere to the advisories [185].

On the contrary, Singapore government and non-profit organisations such as HealthServe took a bundle of policy measures to successfully protect migrant workers after the initial COVID-19 outbreaks in dormitories through enhancing access to support and information and meeting their basic needs of food, housing, and healthcare [188,189]. Singapore government employed extensive testing, established quarantine facilities, offered financial support for employers to pay migrant workers' salaries, supported internet connectivity and prepaid phone cards, and provided timely and free-of-charge medical care for migrant workers infected [188]. In addition to governmental efforts, HealthServe supported migrant workers through several other measures such as building a specific information webpage about COVID-19 that was accessible in different languages and setting up hotline and web-based counselling services [189].

5) People in prison, vulnerability & physical distancing

Institutional or deferential vulnerability is the primary vulnerability that people in prison face as they are deprived of liberty. Besides, they are socially isolated and live in overcrowding spaces, which can lead to social vulnerability. The spatial density in prisons was associated with outcomes of infectious and communicable diseases [293,294]. Vulnerability to mental health issues [295] and violence [296] are also typical among people in prison.

Enforcing social isolation or distancing in prisons had shown to exacerbate mental health issues or even suicide rates among people in prison [297]. Poor communication about the pandemic from prison staff contributed to increased stress and anxiety among people in prison [197]. There had been prison escapes and riots reported in Brazil, Italy, Sudan and Nigeria due to increased social isolation measures such as restrictions on visitors [173,297]. On the contrary, Sweden took additional measures to address the potential negative impacts to people in prison resulted from the enforcement of physical distancing

measures [298]. At the start, Sweden was quick to detect and quarantine suspected cases. In addition, the Swedish Prison and Probation Service (SPPS) provided tablets for people in prison to enable phone calls or video calls with their children or their other family members and friends [298]. Many prison units also organized weekly information meetings to update people in prison about the COVID-19 situation and the policy measures taken to preserve their safety in prisons [298]. These measures were helpful to mitigate fear of contracting the virus among the people in prison and preserve their mental health. Similarly, the UK prisons also allowed virtual or video visits from families [197]. To reduce population density and reduce the spread of COVID-19 in prisons, some governments released a number of people from prisons primarily to home confinement or community supervision, such as in the US, Portugal, Iran, Ireland, Morocco, Libya, Burkina Faso, Uganda and Nigeria [173,195,198,297]. Certain groups of people in prison were considered for rapid release [297], such as those who were approaching their actual release date, eligible for medical release, approved for community supervision, held on minor charges, or held pretrial on bail.

This policy measure can cause unintended problems without deliberate policy design. For instance, in Portugal, the decision was made in a short period without detailed considerations [195] – the president promulgated the policy measure to release people in prison five days after the idea was proposed in the parliament. About 10% of the prison population were released, and they include people in prison serving sentences of under two years, to those nearing the end of their sentences, and to those aged 65 years and above with underlying health conditions. These people in prison were given only one day notice before they were released and had no means of transport to return home at a time when physical distancing measures such as restrictions on public transport were already implemented.

6) People with disabilities, vulnerability & physical distancing

A disability is defined as any physical body or mind condition (impairment) that results in activity limitation (difficulty to do certain activities e.g., difficulty seeing, hearing, walking, or problem solving) or participation restrictions (difficulty participating in normal daily activities e.g., working, social activities, or accessing health care and preventive services)[299]. Health vulnerability is typical for people with disabilities. Economic vulnerability is also a prominent vulnerability reported among the

people with disabilities as they often have less access to educational and employment opportunities, and more likely to live in poverty or live on low incomes [300,301]. Due to the economic vulnerability of people with disabilities, any substantial shock to their financial stability can threaten their access to necessary nutrition, medical care, and proper housing, which may result in further health and economic vulnerabilities [301].

When physical distancing measures were enforced, particularly during the lockdowns, reduction of social connection and reduced access to healthcare services were the challenges that people with disabilities have to face [200]. For instance, in-person supportive services such as community-based rehabilitation may be disrupted [261], and people with mental disabilities (e.g., children with autism) may face worse mental health outcomes due to increased social isolation and suspension of therapy [47,202]. Besides, their reduced mobility and social connection had limited their access to social welfare services such as food rations [156].

In this review, one of the most affected people with disabilities were children with disabilities. When the schools were closed during the pandemic, the lack of necessary technologies (i.e., braille readers) and services (i.e., help and support from special education teachers) at home may lead to their difficulties to participate in remote learning [90]. To support for children with disabilities, school districts in the US, for instance, modified instructions and learning goals to account for the limitations of remote learning, held virtual meetings with school officials, parents and students (when appropriate), and increased collaborations between teachers and parents [85]. Even so, families without computers were unable to attend meetings and webinars. Other solutions to help children with disabilities such as allowing them to borrow learning devices from their schools [90], offering financial support for their educational needs [107], and encouraging parents to facilitate their children to understand the COVID-19 pandemic [47] were also discussed. By and large, it has been challenging for school districts to provide remote learning to students with disabilities because the services and the number of specialised instruction hours may differ for each student[85].

7) Sex workers, vulnerability & physical distancing

By the occupational nature, sex workers face health vulnerability, due to their susceptibility to sexually transmitted infections and social vulnerability such as increased risk to violence [302]. They often face structural barriers when accessing health care, including long waits, unwelcoming spaces, restrictive hours, legal status requirements, lack of transportation, and lack of confidentiality [303]. Some sex workers also face economic vulnerability, such as adverse financial conditions as a result of incurring debts and arrears [304].

In the context of the COVID-19 pandemic, sex workers living with HIV were reported to have experienced the lack of access to testing and treatment for sexually transmitted infections during the lockdown periods [210,212,214]. Besides, they also had limited access to COVID-19 social services and safety nets offered by governments [209]. As an example, in Mysore city, India, antiretroviral therapy (ART) access was disrupted when the city imposed a lockdown. This is because the government-run district hospitals, which were previously tasked with the majority of ART distribution in the city, were designated as testing and treatment centres for COVID-19 in the city at the beginning of the lockdown [210]. Community-based organizations stepped up efforts to provide food, financial aid, COVID-19 safety guideline, and community-driven health interventions including ART, to meet basic needs of sex workers during the COVID-19 pandemic [208,214]. In India, considering that ART was disrupted, the organisation, Ashodaya Samithi, formed a community-led system to distribute ART at private and discreet sites and utilised WhatsApp messaging to share information related to the pandemic [210]. In Thailand, to secure the basic hygiene and personal protection needs for sex workers, community-led organisations which include the Raks Thai Foundation, Dannok Health and Development Community Volunteers and SWING provided food, hand sanitisers, condoms and face masks to the sex workers population [214]. In Africa, community-led outreach also played a role in helping the sex workers by distributing food packs to sex workers [213].

8) Victims of domestic violence, vulnerability & physical distancing

When isolated at home, victims of domestic violence are more socially isolated and have reduced access to institutional support [217]. In many cases, having a disability status can exacerbate the vulnerability to domestic violence [305,306].

Countries/cities such as Argentina, Australia, Bangladesh, Brazil, China, France, Portland, the UK, and the US all observed varying degrees of increase in domestic violence reports or cases during the enforcement of physical distancing measures and lockdowns [217,219,223,226]. Victims of domestic violence may face difficulties in leaving home to access institutional support during the lockdown. School closures that forced children to be home bound had predisposed them to higher risks of violence [74]. In the UK, due to the alarming trends in domestic violence, several national campaigns were organised to raise domestic abuse awareness and highlighted available help services [223]. Helpline services were extended for victims of domestic violence in the UK, and the government had provided funds to support these helplines and other online support services since April 2020 [223]. Victims of domestic violence were allowed to bypass the lockdown restrictions and travelled to sheltered accommodations to seek refuge [223]. Schools and universities had also extended accommodations to students who were vulnerable to domestic violence and ensured student counselling and support services were rendered to support them [223]. Remote counselling services through virtual platforms can be helpful [26]. In France and Italy, governments also commissioned hotels as shelters for victims of domestic violence [217].

9) Refugees, vulnerability & physical distancing

Refugees refer to people who leave their country of origin due to feared persecution, generalized violence, conflict, or “other circumstances that have seriously disturbed public order and, as a result, require international protection” [307]. Vulnerabilities of refugees identified in literature include economic vulnerability [308], social vulnerability (e.g., socially isolated; living in some informal refugee camps that are crowding, lack of water and sanitation facilities, and vulnerable to violent attack) [309,310], and health vulnerability, particularly vulnerability to mental health problems [311].

During the pandemic, precarious and overcrowding housing conditions make it difficult for the refugees to maintain physical distancing or self-isolated at homes when required [231]. Moreover, refugees were usually excluded from financial and social support programs (e.g., food relief program) offered by the destination countries where they live [238]. Due to the shutting down of government services and decreasing number of volunteers serving refugee camps during the pandemic, the refugees resorted to live with limited access to food, basic sanitation, and medical care [231,312]. These situations had exacerbated the mental health issues of refugees who were already living with posttraumatic stress disorders or other mental illness. For some refugees, owing to their sensitive nature of the trauma histories, their forced isolations can conjure up their traumatized memories of the past [231–234,241].

Solutions such as encouraging mask wearing, limiting mobility, sectoring and setting up quarantine areas, and quickly detecting and isolating suspected or confirmed cases, were implemented to limit the virus spread in refugee camps [173,236,237,240]. Mental health services were provided for refugees through phone or video conferencing emotional therapies, as well as through some other voluntary mental health services in a targeted attempt to reach out to the refugee families [232,241]. Mukumbang et al. [238] and O'Connell & Lucić [235] addressed the need to ensure refugees' access to food, financial aid, social support, and offer online learning support for children living in refugee camps.

10) Ethnic minority, vulnerability & physical distancing

An ethnic group normally shares common characteristics such as race, language, religion, or a combination thereof [313]. An ethnic minority refers to an ethnic or racial group of people in a given country where they are in a non-dominant position, and differs from the dominant group in cultural origin, language, religion or race, and have numerically smaller population size than the dominant population [313]. For instance, in the UK, the ethnic minorities are "all ethnic groups except the White British group" [314].

Social vulnerability is typical to ethnic minority group as they are socially isolated/marginalised. They tend to have less access to education and health services than the ethnic majority [313]. Ethnic minorities also face economic vulnerability. In any given country, members of the ethnic minority are

more likely to live in poverty than the ethnic majority on average, and less likely to work in high or semi-skilled jobs [313].

Lockdowns can be particularly challenging for the ethnic minority groups as they were less able to work from home or self-isolated at home [147]. People from the ethnic minority background were more likely to work in adverse employment conditions and experience more financial concerns than the ethnic majority [138,157]. Government assistance programmes in India such as food rations were helpful to households in shortage of food during the COVID-19 period [157]. In some ethnic minority communities in the UK and US, many households comprised multigenerational family members who lived in crowding housing condition which makes it difficult for them to adhere to physical distancing measures [244,249]. Physical distancing measures were also found to substantially impact the mental health of ethnic minorities [248]. Language barrier of the ethnic minorities was also highlighted as an issue. For instance, Khan et al.[245], reported that key public information about the pandemic from the UK government was not intellectual accessible to the Black and Asian ethnic minority groups, that is, lack of translated or visually supportive materials to non-English speakers. This can hamper their understanding of the pandemic and hampered the government's efforts to enforce physical distancing measures [245]. For students from ethnic minority backgrounds, K-12 principals (from kindergarten to 12th grade) in the US offered special education services to support remote learning for non-English speakers [70].

11) People from sexual and gender minorities, vulnerability & physical distancing

Lesbian, gay, bisexual, and transgender (LGBT) is an umbrella term that refers to various sexual and gender minorities [315]. The language and discourse of sexual and gender minority population are dynamic and evolving. Under the umbrella term of LGBT, there are many variants [316], encompassing GLBT (i.e., gay, lesbian, bisexual, and transgender), LGBTI (with the "I" standing for intersex), LGBTQ (with the "Q" standing for queer or questioning), or LGBTIQ. Terms of LGBT+ or LGBTQIA+ (with the "A" standing for asexual, agender, or aromantic) are also common to represent spectrums of sexual and gender minorities. Here we use the term 'people from sexual and gender minorities.' The people from sexual and gender minorities are often socially vulnerable, being socially isolated [316] or vulnerable to violence [317], as well as health vulnerability, particularly vulnerability to HIV

risks [316,317] or mental health illness [251]. The marginalised social identities of people from sexual and gender minorities reinforce and intersect with their health vulnerability. This review has shown that the physical distancing measures to control COVID-19 can exacerbate the social and health vulnerabilities of the people from sexual and gender minorities [251,256,318,319]. Primarily, enforcing physical distancing may limit their access to essential medical services including HIV testing and treatment [256,318]. For those living with mental illness, or those vulnerable to mental illness related to discrimination and lack of family acceptance, the enforcement of physical distancing measures directly cut off their access to supportive friends and partners, reduced the sense of social connectedness, and aggravated feelings of loneliness [258].

To maintain the mental health of the people from sexual and gender minorities, telehealth interventions have been provided in some communities in the UK and US in the forms of web and text-based chats, online video/audio counselling service, or other web-based programs [253,254,257]. There were also practices of offering temporary housing, financial aid, food, and free masks for the people from sexual and gender minorities in India and the US [252,257].

12) Summary of results

As summarised in Table 1, this review has revealed the negative impacts that physical distancing measures had on different vulnerable populations. For the older people, the enforcement of physical distancing measures negatively affected the older people's utilisation of hospital services (i.e., decreased physician consultations and hospital admissions for diseases other than the COVID-19) and resulted in worse mental health outcomes when isolated at home or LTC facilities. In responding to these circumstances, the use of technologies such as virtual meetings had shown to be useful in reducing their loneliness. For children/students, school closures led to concerns about their learning disruptions and decreased social interactions with peers, especially for children from low-income families. To address this, the governments should consider allocating both long-term and short-term funds to help the schools to build remote teaching capacities. Schools should secure provisions for vulnerable children in low-income families, for instance, by redeploying staff to support learning for the vulnerable

children or sending printed materials to those who do not have laptops or printers. For low-income population and migrant workers, job loss and incurred financial challenges were the common challenges faced during lockdowns. Provisions of direct subsidies, job retention schemes, or reemployment services would help lessen these negative impacts. For migrant workers, arrangements should be made for them to return home or connect with their families virtually.

To control the COVID-19 virus transmission in prison settings, some states/countries released a proportion of people in prison, but this hasty measure is not enough to address the situation of the pandemic and should be combined with other policy measures to maintain physical distancing in prisons. For people with disabilities, reduction of family cohesion and reduced access to healthcare support were major problems that occurred when implementing physical distancing measures. Governments are suggested to ensure continued provision of essential healthcare services and special focus on the social protection of the vulnerable such as people with disabilities. For victims of domestic violence, many countries reported increased incidence of domestic violence during the lockdown periods or when stay-at-home orders were executed. Public information campaigns can be organised to raise awareness, and victims of domestic violence should be allowed to bypass the stringent lockdown rules to seek help. Shuttering of government services and decreased number of volunteers serving refugee camps further limit refugees' access to food, basic sanitation and medical care. Dividing refugee camps into subunits and enhance detection and isolation of suspected cases may be helpful to control spread of COVID-19 without affecting the normal life of refugees. In ethnic minority communities, lack of translated or visually supportive materials about COVID-19 to non-English speakers is a challenge and public information distribution measures should be enhanced to help ethnic minority populations to understand COVID-19 and the physical distancing measures. Sex workers and LGBT often experienced disruptions of access to testing and treatment for sexually transmitted infections. Thus, community-led organisations or NGOs play critical roles in offering them hygiene equipment, and essential healthcare services

References

1. Amundsen, D. Digital technologies as a panacea for social isolation and loneliness among older adults: An intervention model for flourishing and wellbeing visual technologies as a panacea for social isolation. *Video J. Educ. Pedagog.* **5**, 1–14 (2021).
2. Daoust, J.-F. Elderly people and responses to COVID-19 in 27 Countries. *PLoS One* **15**, (2020).
3. Tavares, A. I. Older Europeans' experience of unmet health care during the COVID-19 pandemic (first wave). *BMC Health Serv. Res.* **22**, 1–13 (2022).
4. Paccagnella, O. & Pongiglione, B. Depression deterioration of older adults during the first wave of the COVID-19 outbreak in Europe. *Soc. Sci. Med.* **299**, (2022).
5. García-Prado, A., González, P. & Rebollo-Sanz, Y. F. Lockdown strictness and mental health effects among older populations in Europe. *Econ. Hum. Biol.* **45**, (2022).
6. Mendez-Lopez, A., Stuckler, D., McKee, M., Semenza, J. C. & Lazarus, J. V. The mental health crisis during the COVID-19 pandemic in older adults and the role of physical distancing interventions and social protection measures in 26 European countries. *SSM - Popul. Heal.* **17**, (2022).
7. Wester, C. T. *et al.* Longitudinal changes in mental health following the COVID-19 lockdown: Results from the Survey of Health, Ageing and Retirement in Europe. *Ann. Epidemiol.* (2022).
8. Claverie, J.-M. A scenario to safely ease the covid-19 lockdown while allowing economic recovery. *Virologie* **24**, E23–E25 (2020).
9. Visintin, E. P. Contact with older people, ageism, and containment behaviours during the COVID-19 pandemic. *J Community Appl Soc Psychol* **31**, 314–325 (2021).
10. Falcetta, P. *et al.* Impact of COVID-19 lockdown on glucose control of elderly people with type 2 diabetes in Italy. *Diabetes Res. Clin. Pract.* **174**, (2021).
11. Pezzuti, L., Figus, M. & Lauriola, M. Effect of COVID-19 pandemic on older adults' emotion regulation and quality of life after lockdown in Italy. *Psychol. Hub* **38**, 7–13 (2021).
12. Echeverría, P. *et al.* COVIDApp as an innovative strategy for the management and follow-up of COVID-19 cases in long-term care facilities in Catalonia: Implementation study. *JMIR Public Heal. Surveill.* **6**, (2020).
13. Marcos-Pardo, P. J. *et al.* Impact of COVID-19 lockdown on health parameters and muscle strength of older women: A longitudinal study. *Exp. Gerontol.* **164**, (2022).
14. Rodríguez-Gómez, I. *et al.* The medium-term changes in health-related behaviours among Spanish older people lifestyles during COVID-19 lockdown. *J. Nutr. Heal. Aging* **26**, 485–494 (2022).
15. Dura-Perez, E. *et al.* The impact of COVID-19 confinement on cognition and mental health and technology use among socially vulnerable older people: Retrospective cohort study. *J. Med. Internet Res.* **24**, (2022).
16. Michalowsky, B., Hoffmann, W., Bohlken, J. & Kostev, K. Effect of the COVID-19 lockdown on disease recognition and utilisation of healthcare services in the older population in Germany: a cross-sectional study. *Age Ageing* **50**, 317–325 (2021).
17. Yıldırım, H. Psychosocial status of older adults aged 65 years and over during lockdown in Turkey and their perspectives on the outbreak. *Heal. Soc. Care Community* **30**, 899–907 (2022).

18. Paananen, J., Rannikko, J., Harju, M. & Pirhonen, J. The impact of Covid-19-related distancing on the well-being of nursing home residents and their family members: a qualitative study. *Int. J. Nurs. Stud. Adv.* **3**, (2021).

19. Gustafsson, P. E., Nilsson, I. & San Sebastian, M. Venerable vulnerability or remarkable resilience? A prospective study of the impact of the first wave of the COVID-19 pandemic and quarantine measures on loneliness in Swedish older adults with home care. *BMJ Open* **12**, (2022).

20. Park, S. Y. *et al.* Early intervention reduces the spread of covid-19 in long-term care facilities in the Republic of Korea. *Osong Public Heal. Res. Perspect.* **11**, 259–264 (2020).

21. Venturieri, V. R., Goncalves, M. S. & Fuck, V. R. Mitigation of COVID-19 using social distancing of the elderly in Brazil The vertical quarantine effects in hospitalizations and deaths. *medRxiv* **2752**, 1–28 (2021).

22. Shen, J., Wang, C., Dong, C., Tang, Z. & Sun, H. Reductions in mortality resulting from COVID-19 quarantine measures in China. *J. Public Health (Bangkok).* **43**, 254–60 (2021).

23. Sit, R. W. S. *et al.* Explaining the psychosocial effects of covid-19 among older Hong Kong Chinese people—A qualitative analysis. *J. Geriatr. Psychiatry Neurol.* **35**, 206–214 (2022).

24. Bakshi, T. & Bhattacharyya, A. Socially Distanced or Socially Connected? Well-being through ICT Usage among the Indian Elderly during COVID-19. *Millenn. Asia* **12**, 190–208 (2021).

25. Guha, P., Islam, B. & Hussain, M. A. COVID-19 lockdown and penalty of joblessness on income and remittances: A study of inter-state migrant labourers from Assam, India. *J. Public Aff.* **21**, e2470 (2020).

26. Mishra, M. & Majumdar, P. Social distancing during COVID-19: Will it change the Indian society? *J. Health Manag.* **22**, 224–235 (2020).

27. Juhn, Y. J. *et al.* Adherence to public health measures mitigates the risk of COVID-19 infection in older adults: A community-based study. *Mayo Clin. Proc.* **96**, 912–920 (2021).

28. Choi, E. Y., Farina, M. P., Wu, Q. & Ailshire, J. COVID-19 social distancing measures and loneliness among older adults. *Journals Gerontol. Ser. B* 1–12 (2021). doi:10.1093/geronb/gbab009

29. Callow, M. A., Callow, D. D. & Smith, C. Older adults' intention to socially isolate once COVID-19 stay-at-home orders are replaced with "Safer-at-Home" public health advisories: A survey of respondents in Maryland. *J. Appl. Gerontol.* **39**, 1175–1183 (2020).

30. Bowling, C. B. *et al.* Unintended consequences of COVID-19 social distancing among older adults with kidney disease. *Journals Gerontol. - Ser. A Biol. Sci. Med. Sci.* **77**, E133–E137 (2022).

31. Robb, C. E. *et al.* Associations of social isolation with anxiety and depression during the early COVID-19 pandemic: A survey of older adults in London, UK. *Front. Psychiatry* **11**, 591120 (2020).

32. Brown, L. *et al.* Life in lockdown: a telephone survey to investigate the impact of COVID-19 lockdown measures on the lives of older people (≥75 years). *Age Ageing* **50**, 341–346 (2021).

33. Toze, M., Westwood, S. & Hafford-Letchfield, T. Social support and unmet needs among older trans and gender non-conforming people during the COVID-19 'lockdown' in the UK. *Int. J. Transgender Heal.* (2021). doi:10.1080/26895269.2021.1977210

34. McArthur, C. *et al.* Evaluating the effect of COVID-19 pandemic lockdown on long-term care residents' mental health: A data-driven approach in New Brunswick. *J. Am. Med. Dir. Assoc.*

22, 187–192 (2021).

35. Mendoza-Ruvalcaba, N. M. *et al.* Impact of quarantine due to COVID-19 pandemic on health and lifestyle conditions in older adults from Centro American countries. *PLoS One* **17**, (2022).

36. Colebunders, R., Siewe Fodjo, J. N., Vanham, G. & Van den Bergh, R. A call for strengthened evidence on targeted, non-pharmaceutical interventions against COVID-19 for the protection of vulnerable individuals in sub-Saharan Africa. *Int. J. Infect. Dis.* **99**, 482–484 (2020).

37. Siette, J. *et al.* A national survey on COVID-19 second-wave lockdowns on older adults' mental wellbeing, health-seeking behaviours and social outcomes across Australia. *BMC Geriatr.* **21**, (2021).

38. Burzynska, K. & Contreras, G. Gendered effects of school closures during the COVID-19 pandemic. *Lancet* **395**, 1968 (2020).

39. Fantini, M. P., Reno, C., Biserni, G. B., Savoia, E. & Lanari, M. COVID-19 and the re-opening of schools: A policy maker's dilemma. *Ital. J. Pediatr.* **46**, 1–3 (2020).

40. Cheng, S. O. & Liu, A. Debates around the role of school closures in the Coronavirus 2019 Pandemic. *JAMA Pediatr.* **175**, 105–106 (2021).

41. Bahn, G. H. Coronavirus disease 2019, school closures, and children's mental health. *J. Korean Acad. Child Adolesc. Psychiatry* **31**, 74–79 (2020).

42. Ghia, J.-E., Gaulin, S., Ghia, L., Garancher, L. & Flamand, C. Informing children citizens efficiently to better engage them in the fight against COVID-19 pandemic. *PLoS Negl. Trop. Dis.* **14**, e0008828 (2020).

43. Viner, R. M. *et al.* School closure and management practices during coronavirus outbreaks including COVID-19: a rapid systematic review. *Lancet Child Adolesc. Heal.* **4**, 397–404 (2020).

44. The Lancet Child and Adolescent Health. Pandemic school closures: risks and opportunities. *Lancet Child Adolesc. Heal.* **4**, 341 (2020).

45. Fitzpatrick, R., Korin, A. & Riggall, A. *An international review of plans and actions for school reopening*. *Education Development Trust* (2020).

46. Krishnaratne, S. *et al.* Measures implemented in the school setting to contain the COVID-19 pandemic: a rapid scoping review. *Cochrane Database Syst. Rev.* 1–60 (2020). doi:10.1002/14651858.CD013812

47. Singh, S. *et al.* Impact of COVID-19 and lockdown on mental health of children and adolescents: A narrative review with recommendations. *Psychiatry Res.* **293**, 1–10 (2020).

48. Esposito, S. & Principi, N. School closure during the Coronavirus Disease 2019 (COVID-19) Pandemic: An effective intervention at the global level? *JAMA Pediatr.* **174**, 922–923 (2020).

49. Klimek-Tulwin, M. & Tulwin, T. Early school closures can reduce the first-wave of the COVID-19 pandemic development. *J. Public Heal.* (2020). doi:10.1007/s10389-020-01391-z

50. Buffel, V. *et al.* Depressive symptoms in higher education students during the COVID-19 pandemic: The role of containment measures. *Eur. J. Public Health* **32**, 481–487 (2022).

51. Sholihah, I. F., Nurmala, I., Sulistyowati, M. & Devy, S. R. The impact physical distancing during the COVID-19 pandemic on mental health among adolescents: a systematic literature. *Int. J. Public Health* **11**, 69–76 (2022).

52. Rajmil, L. *et al.* Impact of lockdown and school closure on children's health and well-being during the first wave of COVID-19: A narrative review. *BMJ Paediatr. Open* **5**, (2021).

53. Lo Moro, G. *et al.* Reopening schools during the COVID-19 pandemic: Overview and rapid systematic review of guidelines and recommendations on preventive measures and the management of cases. *Int. J. Environ. Res. Public Health* **17**, 1–21 (2020).

54. Russo, G., Jesus, T. S., Deane, K., Osman, A. Y. & McCoy, D. Epidemics, lockdown measures and vulnerable populations: A mixed-methods systematic review of the evidence of impacts on mother and child health in low-and-lower-middle-income countries. *Int. J. Heal. Policy Manag.* Early-Access (2021).

55. Huber, S. G. & Helm, C. COVID-19 and schooling: evaluation, assessment and accountability in times of crises—reacting quickly to explore key issues for policy, practice and research with the school barometer. *Educ. Assessment, Eval. Account.* **32**, 237–270 (2020).

56. Christner, N., Essler, S., Hazzam, A. & Paulus, M. Children's psychological well-being and problem behavior during the COVID-19 pandemic: An online study during the lockdown period in Germany. *PLoS One* **16**, (2021).

57. Montacute, C. & Cullinane, R. Covid-19 and social mobility impact brief #1: School shutdown. *Sutt. Trust* 1–11 (2020).

58. Brooks-Pollock, E., Read, J. M., Mclean, A., Keeling, M. J. & Danon, L. Using social contact data to predict and compare the impact of social distancing policies with implications for school re-opening. *medRxiv* **8**, (2020).

59. Rice, K., Wynne, B., Martin, V., Ackland, G. J. & Ackland. Effect of school closures on mortality from coronavirus disease 2019: Old and new predictions. *BMJ* **371**, (2020).

60. Panovska-Griffiths, J. *et al.* Determining the optimal strategy for reopening schools, the impact of test and trace interventions, and the risk of occurrence of a second COVID-19 epidemic wave in the UK: a modelling study. *Lancet Child Adolesc. Heal.* **4**, 817–827 (2020).

61. Southall, E. *et al.* An analysis of school absences in England during the Covid-19 pandemic. *BMC Med.* **19**, 1–14 (2021).

62. Pollock, A. M. Covid-19: Local implementation of tracing and testing programmes could enable some schools to reopen. *BMJ* **368**, 1–2 (2020).

63. Puntis, J. W. L. Is it really time to go back to school? *Arch Dis Child* **106**, (2021).

64. Catling, J. C., Bayley, A., Begum, Z., Wardzinski, C. & Wood, A. Effects of the COVID-19 lockdown on mental health in a UK student sample. *BMC Psychol.* **10**, (2022).

65. Duby, Z. *et al.* Intersections between COVID-19 and socio-economic mental health stressors in the lives of South African adolescent girls and young women. *Child Adolesc. Psychiatry Ment. Health* **16**, (2022).

66. Morgül, E., Kallitsoglou, A., Essau, C. A. & Castro-Kemp, S. Caregiver-reported changes in the socioemotional wellbeing and daily habits of children with special educational needs during the first COVID-19 national lockdown in the United Kingdom. *Front. Educ.* **7**, (2022).

67. Theis, N., Campbell, N., De Leeuw, J., Owen, M. & Schenke, K. C. The effects of COVID-19 restrictions on physical activity and mental health of children and young adults with physical and/or intellectual disabilities. *Disabil. Health J.* **14**, 101064 (2021).

68. Yui, L. *Landscape measure: Animating the university campus to promote social distancing. New England Journal of Higher Education* (2020).

69. Willis, J., Krausen, K. & Caparas, R. Managing public education resources in California during and after the Coronavirus Crisis: Practical tips and considerations for school district and charter school leaders. Crisis response resource. *WestEd* (2020).

70. Grooms, A. A. & Childs, J. “We Need to Do Better by Kids”: Changing routines in U.S. schools in response to COVID-19 school closures. *J. Educ. Students Placed Risk* **26**, 135–156 (2021).

71. Zimmerman, F. J. & Anderson, N. W. Association of the timing of school closings and behavioral changes with the evolution of the Coronavirus Disease 2019 Pandemic in the US. *JAMA Pediatr.* **175**, 501–509 (2021).

72. Krishnamachari, B. *et al.* The role of mask mandates, stay at home orders and school closure in curbing the COVID-19 pandemic prior to vaccination. *Am. J. Infect. Control* **49**, 1036–1042 (2021).

73. van den Berg, P. *et al.* Effectiveness of 3 versus 6 ft of physical distancing for controlling spread of coronavirus disease 2019 among primary and secondary students and staff: A retrospective, statewide cohort study. *Clin. Infect. Dis.* **02132**, 1–8 (2021).

74. Baron, E. J., Goldstein, E. G. & Wallace, C. T. Suffering in silence: How COVID- 19 school closures inhibit the reporting of child maltreatment. *J. Public Econ.* **190**, 104258 (2020).

75. Burns, A. A. C. & Gutfraind, A. Effectiveness of isolation policies in schools: Evidence from a mathematical model of influenza and COVID-19. *PeerJ* **9**, e11211 (2021).

76. Malkus, N. *Reopening in the shadow of Covid-19: Beginning the first full coronavirus school year*. *American Enterprise Institute* **October**, (2020).

77. Auger, K. A. *et al.* Association between statewide school closure and COVID-19 incidence and mortality in the US. *JAMA - J. Am. Med. Assoc.* **324**, 859–870 (2020).

78. Willis, J., Krausen, K. & Outlaw, L. Managing public education resources during the Coronavirus Crisis: Practical tips and considerations for school district leaders. Crisis response resource. *WestEd* (2020).

79. Melnick, H. & Darling-Hammond, L. *Reopening schools in the context of COVID-19: Health and safety guidelines from other countries. Policy Brief*. *Learning Policy Institute* (2020).

80. Reykdal, C. *Reopening Washington Schools 2020: District Planning Guide*. *Washington Office of Superintendent of Public Instruction* (2020).

81. Wyse, A. E., Stickney, E. M., Butz, D., Beckler, A. & Close, C. N. The potential impact of COVID-19 on student learning and how schools can respond. *Educ. Meas. Issues Pract.* **39**, 60–64 (2020).

82. Donohue, J. M. & Miller, E. COVID-19 and school closures. *JAMA - J. Am. Med. Assoc.* **324**, 845–847 (2020).

83. DeArmond, M., Chu, L. & Gundapaneni, P. How are school districts addressing student social-emotional needs during the pandemic? *Center on Reinventing Public Education* (2021).

84. von Schroeter, M., Weiss, N. & O’rourke, T. *How should Massachusetts reopen its K-12 schools in the fall? Lessons from abroad and other states*. *Pioneer Institute for Public Policy Research* (2020).

85. Nowicki, J. M. *Distance learning: Challenges providing services to K-12 English learners and students with disabilities during COVID-19*. *Government Accountability Office (GAO) Report to Congressional Committees* (2020).

86. Grimes, A. *et al.* Decreased physical activity among youth resulting from COVID-19 pandemic-related school closures: Natural experimental study. *JMIR Form. Res.* **6**, (2022).

87. Prata- Linhares, M. M., Cardoso, T. D. S. G., Lopes-Jr, D. S. & Zukowsky-Tavares, C. Social distancing effects on the teaching systems and teacher education programmes in Brazil:

reinventing without distorting teaching. *J. Educ. Teach.* **46**, 554–564 (2020).

88. Friedrich, F. *et al.* Early Impact of social distancing in response to COVID-19 on hospitalizations for acute bronchiolitis in infants in Brazil. *Clin. Infect. Dis.* **72**, 2071–2075 (2021).

89. Loima, J. Socio-educational policies and Covid-19 – A case study on Finland and Sweden in the Spring 2020. *Int. J. Educ. Lit. Stud.* **8**, 59–75 (2020).

90. Pellegrini, M. & Maltinti, C. "School never stops": Measures and experience in Italian schools during the COVID-19 lockdown. *Best Evid. Chinese Educ.* **5**, 649–663 (2020).

91. Caroppo, E. *et al.* Unequal effects of the national lockdown on mental and social health in Italy. *Ann. Ist. Super. Sanita* **56**, 497–501 (2020).

92. Di Giorgio, E., Polli, R., Lunghi, M. & Murgia, A. Impact of the COVID-19 italian lockdown on the physiological and psychological well-being of children with fragile x syndrome and their families. *Int. J. Environ. Res. Public Health* **18**, (2021).

93. Rypdal, M. *et al.* Modelling suggests limited change in the reproduction number from reopening norwegian kindergartens and schools during the COVID-19 pandemic. *PLoS One* **16**, e0238268 (2021).

94. Myhr, A., Naper, L. R., Samarawickrema, I. & Vesterbekkmo, R. K. Impact of COVID-19 pandemic lockdown on mental well-being of Norwegian adolescents during the first wave—Socioeconomic position and gender differences. *Front. Public Heal.* **9**, (2021).

95. Godøy, A., Grøtting, M. W. & Hart, R. K. Reopening schools in a context of low COVID-19 contagion: consequences for teachers, students and their parents. *J. Popul. Econ.* **35**, 935–961 (2022).

96. Jensen, S. S. & Reimer, D. The effect of COVID-19-related school closures on students' well-being: Evidence from Danish nationwide panel data. *SSM - Popul. Heal.* **16**, (2021).

97. Boterberg, S. *et al.* The impact of COVID-19 measures on parent-reported restricted and repetitive behaviours in pre-school children with autism. *Heliyon* **8**, (2022).

98. Lindell-Postigo, D., Zurita-Ortega, F., Ortiz-Franco, M. & González-Valero, G. Cross-sectional study of self-concept and gender in relation to physical activity and martial arts in Spanish adolescents during the COVID-19 lockdown. *Educ. Sci.* **10**, 1–10 (2020).

99. Yayci, L. & Kendirci, A. Determination of educational/academic and some social behavioral trends of elementary school students in Turkey during Coronavirus (Covid-19) pandemic days. *Int. Online J. Educ. Teach.* **8**, 358–380 (2021).

100. Formosinho, J. From schoolification of children to schoolification of parents?–educational policies in COVID times. *Eur. Early Child. Educ. Res. J.* **29**, 141–152 (2021).

101. Meguid, N. A. *et al.* Influence of COVID-19 pandemic lockdown on a sample of Egyptian children with Down syndrome. *Egypt. J. Med. Hum. Genet.* **23**, 1–8 (2022).

102. Bourion-Bédès, S. *et al.* The effects of living and learning conditions on the health-related quality of life of children and adolescents during the COVID-19 lockdown in the French Grand Est region. *BMC Public Health* **22**, (2022).

103. Berard, M. *et al.* Impact of containment and mitigation measures on children and youth with SD during the COVID-19 pandemic: Report from the ELENA cohort. *J. Psychiatr. Res.* **137**, 73–80 (2021).

104. Luijten, M. A. J. *et al.* The impact of lockdown during the COVID-19 pandemic on mental and social health of children and adolescents. *Qual. Life Res.* **30**, 2795–2804 (2021).

105. Zijlmans, J. *et al.* Mental and social health of children and adolescents with pre-existing mental or somatic problems during the COVID-19 pandemic lockdown. *Front. Psychiatry* **12**, (2021).

106. Seymour, K., Skattebol, J. & Pook, B. Compounding education disengagement: COVID-19 lockdown, the digital divide and wrap-around services. *J. Child. Serv.* **15**, 243–251 (2020).

107. Yates, S., Dickinson, H., Smith, C. & Tani, M. Flexibility in individual funding schemes: How well did Australia's National Disability Insurance Scheme support remote learning for students with disability during COVID-19? *Soc. Policy Adm.* **55**, 906–920 (2020).

108. McNicholas, J., Hammersley, M. L., Hopkins, S., McDermott, S. & Plaskett, J. The impact of COVID-19 restrictions on the healthy eating and movement behaviors of 0–12-year-old children in Western Sydney, Australia. *Front. Public Heal.* **10**, (2022).

109. Naimark, D. *et al.* Simulation-based estimation of Sars-cov-2 infections associated with school closures and community-based nonpharmaceutical interventions in Ontario, Canada. *JAMA Netw Open* **4**, 1–13 (2021).

110. Lee, B., Hanley, J. P., Nowak, S., Bates, J. H. T. & Hébert-Dufresne, L. Modeling the impact of school reopening on SARS-CoV-2 transmission using contact structure data from Shanghai. *BMC Public Health* **20**, 1–9 (2020).

111. Biswas, S. & Biswas, A. Anxiety level among students of different college and universities in India during lock down in connection to the COVID-19 pandemic. *J. Public Heal.* **31**, 49–55 (2021).

112. Babu, T. A. & Selvapandiyan, J. The psychological effects of COVID-19 Pandemic related lockdown in children. *Indian Pediatr.* **57**, 1087 (2020).

113. Manjareeka, M. & Pathak, M. COVID-19 lockdown anxieties: Is student a vulnerable group? *J. Indian Assoc. Child Adolesc. Ment. Heal.* **17**, 72–80 (2020).

114. Thakur, K., Kumar, N. & Sharma, N. R. Effect of the Pandemic and Lockdown on Mental Health of Children. *Indian J. Pediatr.* **87**, 552 (2020).

115. Chitapure, T., Bhodaji, S., Zanwar, M. & Malani, R. Impact of covid-19 pandemic lockdown on mental and physical health of students: A cross-sectional study. *Int. J. Res. Pharm. Sci.* **11**, 1710–1714 (2020).

116. Kumar, S. *et al.* Socio-economic impact of first 21 days nationwide lockdown- 1 on the spread of SARS-COV-2 in India in relation to health. *J. Fam. Med. Prim. Care* **9**, 4557–4562 (2020).

117. Kaur, R., Boobna, T. & Kallingal, P. Effect of Covid-19 lockdown on Indian children with autism. *Res. Dev. Disabil.* **125**, 104230 (2022).

118. Ursache, A. *et al.* A quasi-experimental study of parent and child well-being in families of color in the context of COVID-19 related school closure. *SSM - Popul. Heal.* **17**, (2022).

119. Gothwal, V. K., Kodavati, K. & Subramanian, A. Life in lockdown: impact of COVID-19 lockdown measures on the lives of visually impaired school-age children and their families in India. *Ophthalmic Physiol. Opt.* **42**, 301–310 (2022).

120. Tajane, I., Golwala, A., Nangia, D. & Chavan, I. Impact of covid-19 lockdown on physical and mental health of 5-12 years old children; from parents' perspective: A cross-sectional study. *Asia Pacific J. Heal. Manag.* **16**, (2021).

121. Shah, R., Raju, V. V, Sharma, A. & Grover, S. Impact of COVID-19 and lockdown on children with ADHD and their families-An online survey and a continuity care model. *J. Neurosci. Rural Pract.* **12**, 71–79 (2021).

122. Sifat, R. I., Ruponty, M. M., Rahim Shuvo, M. K., Chowdhury, M. & Suha, S. M. Impact of COVID-19 pandemic on the mental health of school-going adolescents: Insights from Dhaka city, Bangladesh. *Heliyon* **8**, (2022).

123. Iwata, K., Doi, A. & Miyakoshi, C. Was school closure effective in mitigating coronavirus disease 2019 (COVID-19)? Time series analysis using Bayesian inference. *Int. J. Infect. Dis.* **99**, 57–61 (2020).

124. Takaku, R. & Yokoyama, I. What the COVID-19 school closure left in its wake: Evidence from a regression discontinuity analysis in Japan. *J. Public Econ.* **195**, 104364 (2021).

125. Nomura, K. *et al.* Cross-sectional survey of depressive symptoms and suicide-related ideation at a Japanese national university during the COVID-19 stay-home order. *Environ. Health Prev. Med.* **26**, (2021).

126. Yadav, R. K. *et al.* Anxiety and depression among health sciences students in home quarantine during the COVID-19 Pandemic in selected provinces of Nepal. *Front. Public Heal.* **9**, (2021).

127. Ali, A., Siddiqui, A. A., Arshad, M. S., Iqbal, F. & Arif, T. Bin. Effects of COVID-19 pandemic and lockdown on lifestyle and mental health of students: A retrospective study from Karachi, Pakistan. *Ann. Med. Psychol. (Paris).* (2021). doi:10.1016/j.amp.2021.02.004

128. Nyamukamba, P., Mujuru, N. M. & Isaacs, S. Early impacts of covid-19 and government's intervention and prevention actions in South Africa. *J. Hum. Ecol.* **71**, 266–276 (2020).

129. Obuaku-Igwe, C. 'Umuntu ngumuntu ngabantu' (a person is a person because of other people): reflections on student's experiences of social isolation and the impact of a peer to peer mental health support group during covid-19 lockdown in South Africa. *Youth Voice J.* **2020**, 58–71 (2020).

130. Nyashanu, M., Simbanegavi, P. & Gibson, L. Exploring the impact of COVID-19 pandemic lockdown on informal settlements in Tshwane Gauteng Province, South Africa. *Glob. Public Health* **15**, 1443–1453 (2020).

131. Kaisara, G. & Bwalya, K. J. Investigating the e-learning challenges faced by students during COVID-19 in Namibia. *International Journal of Higher Education* **10**, 308–318 (2021).

132. Zulaika, G. *et al.* Impact of COVID-19 lockdowns on adolescent pregnancy and school dropout among secondary schoolgirls in Kenya. *BMJ Glob. Heal.* **7**, (2022).

133. Al-Iede, M. *et al.* Impact of COVID-19 lockdown on children with asthma in Jordan: A parental questionnaire. *BMJ Paediatr. Open* **5**, e001136 (2021).

134. Alon, T., Kim, M., Lagakos, D. & Vanvuren, M. How should policy responses to the covid-19 pandemic differ in the developing world? Working Paper Series No. 27273. *Natl. Bur. Econ. Res.* (2020).

135. Silva, D. S. & Smith, M. J. Social distancing, social justice, and risk during the COVID-19 pandemic. *Can. J. Public Heal.* **111**, 459–461 (2020).

136. Dahab, M. *et al.* COVID-19 control in low-income settings and displaced populations: What can realistically be done? *Confl. Health* **14**, 1–6 (2020).

137. Barnett-Howell, Z., Mobarak, A. M. & Watson, O. J. The benefits and costs of social distancing in high- and low-income countries. *Trans R Soc Trop Med Hyg* **115**, 807–819 (2021).

138. Denford, S. *et al.* Understanding patterns of adherence to COVID-19 mitigation measures: a qualitative interview study. *J. Public Health (Bangkok).* **43**, 508–516 (2021).

139. Basu, P., Bell, C. & Edwards, T. H. COVID social distancing and the poor: An analysis of the

evidence for England. *B.E. J. Macroecon.* **22**, 211–240 (2022).

140. Basu, P., Bell, C. & Edwards, T. H. COVID social distancing and the poor: An analysis of the evidence for England. *B.E. J. Macroecon.* **22**, 211–240 (2022).

141. Banerjee, T., Nayak, A. & Zhao, H. Y. A county-level study of the effects of state-mandated COVID-19 lockdowns on urban and rural restaurant visits using consumers' cell phone geo-location data. *J. Public Heal. From Theory to Pract.* **31**, 249–258 (2021).

142. Jung, J., Manley, J. & Shrestha, V. Coronavirus infections and deaths by poverty status: The effects of social distancing. *J. Econ. Behav. Organ.* **182**, 311–330 (2021).

143. Kerkhoff, A. D. *et al.* Evaluation of a novel community-based COVID-19 'Test-to-Care' model for low-income populations. *PLoS One* **15**, e0239400 (2020).

144. Yilmazkuday, H. COVID-19 and unequal social distancing across demographic groups. *Reg. Sci. Policy Pract.* **12**, 1235–1248 (2020).

145. Chang, H.-Y. *et al.* Differential impact of mitigation policies and socioeconomic status on COVID-19 prevalence and social distancing in the United States. *BMC Public Health* **21**, 1–10 (2021).

146. Alderman, J. & Harjoto, M. COVID-19: US shelter-in-place orders and demographic characteristics linked to cases, mortality, and recovery rates. *Transform. Gov. People, Process Policy* **15**, 627–644 (2020).

147. Singh, S., Shaikh, M., Hauck, K. & Miraldo, M. Impacts of introducing and lifting nonpharmaceutical interventions on COVID-19 daily growth rate and compliance in the United States. *Proc. Natl. Acad. Sci.* **118**, e2021359118 (2021).

148. Jay, J. *et al.* Neighbourhood income and physical distancing during the COVID-19 pandemic in the United States. *Nat. Hum. Behav.* **4**, 1294–1302 (2020).

149. Lou, J., Shen, X. & Niemeier, D. Are stay-at-home orders more difficult to follow for low-income groups? *J. Transp. Geogr.* **89**, 102894 (2020).

150. Weill, J. A., Stigler, M., Deschenes, O. & Springborn, M. R. Social distancing responses to COVID-19 emergency declarations strongly differentiated by income. *Proc. Natl. Acad. Sci.* **117**, 19658–19660 (2020).

151. Garnier, R., Benetka, J. R., Kraemer, J. & Bansal, S. Socioeconomic disparities in social distancing during the COVID-19 pandemic in the United States: Observational study. *J. Med. Internet Res.* **23**, 1–8 (2021).

152. Mandal, K. K., Alam, S., Ranjan, A., Dharanirajan, K. & Kumari, P. A review of India's response to COVID-19 outbreak and impact of lockdown on Indian economy. *J. Crit. Rev.* **7**, 402–408 (2020).

153. Agoramoorthy, G. & Hsu, M. J. How the Coronavirus lockdown impacts the impoverished in India. *J. Racial Ethn. Heal. Disparities* **8**, 1–6 (2021).

154. Sahu, M. & Dobe, M. How the largest slum in india flattened the covid curve? A case study. *South East. Eur. J. Public Heal.* **14**, 1–15 (2020).

155. Wasdani, K. P. & Prasad, A. The impossibility of social distancing among the urban poor: the case of an Indian slum in the times of COVID-19. *Local Environ.* **25**, 414–418 (2020).

156. Mathias, K., Rawat, M., Philip, S. & Grills, N. 'We've got through hard times before': acute mental distress and coping among disadvantaged groups during COVID-19 lockdown in North India - a qualitative study. *Int. J. Equity Health* **19**, 1–12 (2020).

157. Singh, I., Singh, J. & Baruah, A. Income and employment changes under COVID-19 lockdown: A study of Urban Punjab. *Millenn. Asia* **11**, 391–412 (2020).

158. Ramesh, R. M. *et al.* Impact of the COVID-19 national lockdown on a rural and tribal population of Tamil Nadu, Southern India: A mixed-methods Survey. *Am. J. Trop. Med. Hyg.* **106**, 1498–1506 (2022).

159. Pandey, G., Moghal, S., Barodia, R. & Carey, W. COVID-19 and its effects on the mental health of people living in urban slums in India. *J. Inf. Knowl. Manag.* **21**, 2240003 (2022).

160. Auerbach, A. M. & Thachil, T. How does Covid-19 affect urban slums? Evidence from settlement leaders in India. *World Dev.* **140**, (2021).

161. Bennett, M. All things equal? Heterogeneity in policy effectiveness against COVID-19 spread in Chile. *World Dev.* **137**, 105208 (2021).

162. Khan, M., Roy, P., Matin, I., Rabbani, M. & Chowdhury, R. An adaptive governance and health system response for the COVID-19 emergency. *World Dev.* **137**, (2021).

163. Jorge, D. C. P. *et al.* Assessing the nationwide impact of Covid-19 mitigation policies on the transmission rate of SARS-CoV-2 in Brazil. *Epidemics* **35**, 100465(1–11) (2021).

164. Kazevman, G., Mercado, M., Hulme, J. & Somers, A. Prescribing phones to address health equity needs in the COVID-19 Era: The PHONE-CONNECT program. *J. Med. Internet Res.* **23**, (2021).

165. Liu, X., Zhang, D., Sun, T., Li, X. & Zhang, H. Containing COVID-19 in rural and remote areas: Experiences from China. *J. Travel Med.* **27**, 1–4 (2020).

166. Ma, L. *et al.* Knowledge, beliefs/attitudes, and practices of rural residents in the prevention and control of COVID-19: An online questionnaire survey. *Am. J. Trop. Med. Hyg.* **103**, 2357–2367 (2020).

167. Lau, L. L., Hung, N. & Wilson, K. COVID-19 response strategies: Considering inequalities between and within countries. *Int. J. Equity Health* **19**, 1–3 (2020).

168. Bonaccorsi, G. *et al.* Economic and social consequences of human mobility restrictions under COVID-19. *Proc. Natl. Acad. Sci.* **117**, 15530–15535 (2020).

169. Romero-Michel, J. C. *et al.* Health and economic measures in response to the COVID-19 pandemic- Effect on street vendors. *J. Infect. Dev. Ctries.* **15**, 198–203 (2021).

170. Nachega, J. B. *et al.* Contact tracing and the COVID-19 response in Africa: Best practices, Key Challenges, and lessons learned from Nigeria, Rwanda, South Africa, and Uganda. *Am. J. Trop. Med. Hyg.* **104**, 1179–1187 (2021).

171. Quaife, M. *et al.* The impact of COVID-19 control measures on social contacts and transmission in Kenyan informal settlements. *BMC Med.* **18**, 1–11 (2020).

172. Mbogo, R. W. & Odhiambo, J. W. COVID-19 outbreak, social distancing and mass testing in Kenya-insights from a mathematical model. *Afrika Mat.* (2021). doi:10.1007/s13370-020-00859-1

173. Oladimeji, O., Atiba, B. P., A Mbokazi, J. & Hyera, F. L. M. The Homeless, Inmates and Refugees in Africa in the Face of COVID-19 Outbreak. *Open Public Health J.* **13**, 306–308 (2020).

174. Jiang, X., Gong, W., Gao, Y., Fraser, C. & Holmes, C. Modelling the impact of rapid tests, tracing and distancing in lower-income countries suggest optimal policies varies with rural-urban settings. *medRxiv* (2021). doi:10.1101/2021.03.17.21253853

175. Ward, M. South Africa's COVID-19 lockdown dilemma. *Emerald Emerg. Mark. Case Stud.* **10**, 1–10 (2020).

176. Arndt, C. *et al.* Covid-19 lockdowns, income distribution, and food security: An analysis for South Africa. *Glob. Food Sec.* **26**, 100410 (2020).

177. Iwara, I. O., Musvipwa, F., Amaechi, K. E. & Raselekoane, R. COVID-19 lock-down socio-economic challenges faced by households in rural areas: A perspective from Vhembe district, South Africa. *Sociol. Pregl.* **54**, 761–798 (2020).

178. McIntosh, A. *et al.* Effect of COVID-19 lockdown on hospital admissions and mortality in rural KwaZulu-Natal, South Africa: Interrupted time series analysis. *BMJ Open* **11**, 1–10 (2021).

179. Iwuoha, V. C. & Aniche, E. T. Covid-19 lockdown and physical distancing policies are elitist: towards an indigenous (Afro-centred) approach to containing the pandemic in sub-urban slums in Nigeria. *Local Environ.* **25**, 631–640 (2020).

180. Obasi, C. O. & Anierobi, C. M. The homeless-poor and the COVID-19 stay-at-home policy of government: Rethinking the plight of homelessness in Nigeria. *SAGE Open* **11**, (2021).

181. Mahmud, M. & Riley, E. Household response to an extreme shock: Evidence on the immediate impact of the Covid-19 lockdown on economic outcomes and well-being in rural Uganda. *World Dev.* **140**, (2021).

182. Dzawanda, B., Matsa, M. & Nicolau, M. Poverty on the rise: The impact of the COVID-19 lockdown on the informal sector of Gweru, Zimbabwe. *Int. Soc. Sci. J.* **71**, 81–96 (2021).

183. Choi, K. *et al.* Life during lockdown: a qualitative study of low-income New Zealanders' experience during the COVID-19 pandemic. *N. Z. Med. J.* **134**, 52–67 (2021).

184. Choudhari, R. COVID 19 pandemic: Mental health challenges of internal migrant workers of India. *Asian J. Psychiatry J.* **54**, 102254 (2020).

185. Kumar, S. & Choudhury, S. Migrant workers and human rights: A critical study on India's COVID-19 lockdown policy. *Soc. Sci. Humanit. Open* **3**, 100130 (2021).

186. Singh, G. P. & Arun, P. Subjective well-being among migrant workers residing in a shelter home during lockdown: A pilot survey from North India. *Prim. care companion CNS Disord.* **24**, 38916 (2021).

187. Melillo, T. Public health measures to control the spread of COVID-19 amongst the migrant population in Malta. *Eur. J. Public Health* **30**, ckaa165.429 (2020).

188. Yee, K. *et al.* Stressors and coping strategies of migrant workers diagnosed with COVID-19 in Singapore: A qualitative study. *BMJ Open* **11**, 1–8 (2021).

189. Chan, L. G. & Kuan, B. Mental health and holistic care of migrant workers in Singapore during the Covid-19 pandemic. *J. Glob. Health* **10**, 1–5 (2020).

190. Sohel, M. S. *et al.* COVID-19 induced impact on informal migrants in Bangladesh: A qualitative study. *Int. J. Sociol. Soc. Policy* **42**, 473–497 (2022).

191. Crouzet, L. *et al.* Impact of the COVID-19 pandemic on vulnerable groups, including homeless persons and migrants, in France: A qualitative study. *Prev. Med. Reports* **26**, 101727 (2022).

192. Fu, L., Lindenmeyer, A., Phillimore, J. & Lessard-Phillips, L. Vulnerable migrants' access to healthcare in the early stages of the COVID-19 pandemic in the UK. *Public Health* **203**, 36–42 (2022).

193. Bojórquez, I., Odgers-Ortiz, O. & Olivas-Hernández, O. L. Psychosocial and mental health during the COVID-19 lockdown: A rapid qualitative study in migrant shelters at the Mexico-United States border. *Salud Ment.* **44**, 167–175 (2021).

194. Henry, B. F. Social distancing and incarceration: policy and management strategies to reduce COVID-19 transmission and promote health equity through decarceration. *Heal. Educ. Behav.* **47**, 536–539 (2020).

195. Fróis, C. COVID-19 pandemic and social distancing in prisons. *Anthropol. Today* **36**, 25–26 (2020).

196. Lindström, M., Ahlstrand, E. & Kärrholm, J. Sweden's Response to the COVID-19 Outbreak. *Vict. Offenders* **15**, 1203–1214 (2020).

197. Maycock, M. & Dickson, G. Analysing the views of people in custody about the management of the COVID-19 pandemic in the Scottish Prison Estate. *Int. J. Prison. Health* (2021). doi:10.1108/IJPH-09-2020-0065

198. Schotland, S. D. A plea to apply principles of quarantine ethics to prisoners and immigration detainees during the COVID-19 crisis. *J. Law Biosci.* 1–11 (2020). doi:10.1093/jlb/lsaa070

199. Duarte, C. *et al.* COVID-19 outbreak in a state prison: a case study on the implementation of key public health recommendations for containment and prevention. *BMC Public Health* **22**, (2022).

200. Dalise, S. *et al.* Psycho-social impact of social distancing and isolation due to the COVID-19 containment measures on patients with physical disabilities. *Eur. J. Phys. Rehabil. Med.* **57**, 158–165 (2021).

201. Chirico, I. *et al.* The impact of COVID-19 restrictions and care home strategies on residents with dementia as experienced by family carers in Italy. *Aging Ment. Heal.* (2022). doi:10.1080/13607863.2022.2056137

202. Solé, B. *et al.* Effects of the COVID-19 pandemic and lockdown in Spain: comparison between community controls and patients with a psychiatric disorder. Preliminary results from the BRIS-MHC STUDY. *J. Affect. Disord.* **281**, 13–23 (2021).

203. Goddard, K. S., Schulz, J., Nzuki, I. & Hall, J. P. Examining the impacts of the coronavirus pandemic and social distancing on the health of people with mobility disabilities. *Front. Public Heal.* **10**, 875880 (2022).

204. Benjenk, I. *et al.* Impact of COVID-19 Mitigation efforts on adults with serious mental Illness. *J. Nerv. Ment. Dis.* **209**, 892–898 (2021).

205. Hanna, K. *et al.* Emotional and mental wellbeing following COVID-19 public health measures on people living with dementia and carers. *J. Geriatr. Psychiatry Neurol.* **35**, 344–352 (2022).

206. Tsapanou, A., Zoi, P., Kalligerou, F., Blekou, P. & Sakka, P. The effect of prolonged lockdown due to COVID-19 on Greek demented patients of different stages and on their caregivers. *J. Alzheimer's Dis.* **83**, 907–913 (2021).

207. Folostina, R. & Iacob, C. I. Impact of COVID-19 lockdown on children with developmental disabilities and their parents. *Cypriot J. Educ. Sci.* **16**, 1878–1892 (2021).

208. Callander, D. *et al.* Sex workers are returning to work and require enhanced support in the face of COVID-19: Results from a longitudinal analysis of online sex work activity and a content analysis of safer sex work guidelines. *Sex. Health* **17**, 384–386 (2020).

209. Adebisi, Y. A. *et al.* Sex Workers Should not Be Forgotten in Africa's COVID-19 Response. *Am. J. Trop. Med. Hyg.* **103**, 1780–1782 (2020).

210. Reza-Paul, S. *et al.* Community action for people with HIV and sex workers during the COVID-19 pandemic in India. *WHO South-East Asia J. public Heal.* **9**, 104–106 (2020).

211. Pollard, R. *et al.* HIV service delivery in the time of COVID-19: focus group discussions with key populations in India. *J. Int. AIDS Soc.* **24**, (2021).

212. Macharia, P. *et al.* Sexual health among Kenyan male sex workers in a time of COVID-19. *Health Educ. J.* **80**, 119–127 (2021).

213. Gichuna, S. *et al.* Access to healthcare in a time of COVID-19: Sex workers in crisis in Nairobi, Kenya. *Glob. Public Health* **15**, 1430–1442 (2020).

214. Janyam, S. *et al.* Protecting sex workers in Thailand during the COVID-19 pandemic: opportunities to build back better. *WHO South-East Asia J. public Heal.* **9**, 100–103 (2020).

215. Tan, R. K. J. *et al.* COVID-19 and the shifting organisation of sex work markets in Singapore. *Cult. Heal. Sex.* (2021). doi:10.1080/13691058.2021.2014975

216. Avwioro, G. *et al.* Commercial sex work during novel coronavirus (SARS-CoV-2) era in the niger delta region: Relationships between knowledge, preventive practice, and transmission potential. *Int. J. Womens. Health* **13**, 509–523 (2021).

217. Usher, K., Bhullar, N., Durkin, J., Gyamfi, N. & Jackson, D. Family violence and COVID-19: Increased vulnerability and reduced options for support. *Int. J. Ment. Health Nurs.* **29**, 549–552 (2020).

218. Jain, B., Jain, S. & Khan, A. Covid-19 lockdown: A greater impact on women. *Natl. Med. J. India* **34**, 238–239 (2022).

219. Hamadani, J. D. *et al.* Immediate impact of stay-at-home orders to control COVID-19 transmission on socioeconomic conditions, food insecurity, mental health, and intimate partner violence in Bangladeshi women and their families: an interrupted time series. *Lancet Glob. Heal.* **8**, e1380–e1389 (2020).

220. Mahmood, N., Kamruzzaman, M., Rahman, A., Reidpath, D. D. & Akhter, S. Impact of the COVID-19 lockdown on intimate partner violence: Issues of non-reporting in Bangladesh. *Women's Heal.* **18**, (2022).

221. Jahid, A. Unsafe at home: the increased trend of domestic violence during the COVID-19 pandemic in Bangladesh. *J. Adult Prot.* **24**, 15–21 (2022).

222. Tripathi, P., Dwivedi, P. S. & Sharma, S. Psychological impact of domestic violence on women in India due to COVID-19. *Int. J. Hum. Rights Healthc.* (2022). doi:10.1108/IJHRH-12-2021-0208

223. Universities UK. *Beginning the conversation: responding to domestic violence and abuse in higher education communities during Covid-19 pandemic*. **April**, (2020).

224. Rengasamy, E. R. *et al.* Impact of COVID-19 lockdown: Domestic and child abuse in Bridgend. *Child Abus. Negl.* **130**, (2022).

225. Vives-cases, C., La Parra-Casado, D., Estévez, J. F., Torrubiano-domínguez, J. & Sanz-barbero, B. Intimate partner violence against women during the COVID-19 lockdown in Spain. *Int. J. Environ. Res. Public Health* **18**, (2021).

226. Boserup, B., McKenney, M. & Elkbuli, A. Alarming trends in US domestic violence during the COVID-19 pandemic. *Am. J. Emerg. Med.* **38**, 2753–2755 (2020).

227. Nduna, M. & Tshona, S. O. Domesticated poly-violence against women during the 2020 Covid-19 lockdown in South Africa. *Psychol. Stud. (Mysore).* **66**, 347–353 (2021).

228. Iesue, L., Casanova, F. O. & Piquero, A. R. Domestic violence during a global pandemic: Lockdown policies and their impacts across Guatemala. *J. Contemp. Crim. Justice* **37**, 589–614 (2021).

229. Ditekemena, J. D. *et al.* Intimate partners violence against women during a covid-19 lockdown period: Results of an online survey in 7 provinces of the Democratic Republic of Congo. *Int. J. Environ. Res. Public Health* **18**, (2021).

230. Nesset, M. B., Gudde, C. B., Mentzoni, G. E. & Palmstierna, T. Intimate partner violence during COVID-19 lockdown in Norway: the increase of police reports. *BMC Public Health* **21**, (2021).

231. Júnior, J. G. *et al.* A crisis within the crisis: The mental health situation of refugees in the world during the 2019 coronavirus (2019-nCoV) outbreak. *Psychiatry Res.* **288**, 1–2 (2020).

232. Pinzón-Espinosa, J., Valdés-Florido, M. J., Riboldi, I., Baysak, E. & Vieta, E. The COVID-19 Pandemic and Mental Health of Refugees, Asylum Seekers, and Migrants. *J. Affect. Disord.* **280**, 407–408 (2021).

233. Peprah, P. Ageing out of place in COVID-19 pandemic era: How does the situation look like for older refugees in camps? *Arch. Gerontol. Geriatr.* **90**, (2020).

234. Alemi, Q., Stempel, C., Siddiq, H. & Kim, E. Refugees and covid-19: Achieving a comprehensive public health response. *Bull. World Health Organ.* **98**, 510-510A (2020).

235. O'Connell, C. & Lucić, L. An informal education intervention in response to the Covid-19 pandemic: homework mentorships in a Berlin refugee shelter. *Hum. Arenas* **4**, 616–631 (2021).

236. Spernovasilis, N., Markaki, L. & Tsioutis, C. Challenges posed by COVID-19 to refugee camps on the Greek islands: We are all humans after all Letter to the Editor. *Pneumon* **33**, (2020).

237. Gilman, R. T., Mahroof-Shaffi, S., Harkensee, C. & Chamberlain, A. T. Modelling interventions to control COVID-19 outbreaks in a refugee camp. *BMJ Glob. Heal.* **5**, 1–9 (2020).

238. Mukumbang, F. C., Ambe, A. N. & Adebiyi, B. O. Unspoken inequality: How COVID-19 has exacerbated existing vulnerabilities of asylum-seekers, refugees, and undocumented migrants in South Africa. *Int. J. Equity Health* **19**, 1–7 (2020).

239. Walker, L., Bailey, D., Churchill, R. & Peckham, E. Remote data collection during COVID-19 restrictions: an example from a refugee and asylum-seeker participant group in the UK. *Trials* **22**, (2021).

240. Leiner, A., Sammon, M., Perry, H. & Dunavant, S. Facing COVID-19 and refugee camps on the U.S. Border. *J. Emerg. Med.* **59**, 143–145 (2020).

241. Mattar, S. & Piwowarczyk, L. A. COVID-19 and U.S.-based refugee populations: Commentary. *Psychol Trauma* **12**, 228–229 (2020).

242. Al-Awaida, W. *et al.* Psychological effects of quarantine on Syrian refugees, compared to the Jordanian populations. *Int. Migr.* (2021). doi:10.1111/imig.12847

243. Ceccon, C. & Moscardino, U. Impact of COVID-19 and lockdown on mental health and future orientation among young adult asylum seekers in Italy: A mixed-methods study. *Transcult. Psychiatry* (2022). doi:10.1177/13634615221098306

244. Mathur, R., Bear, L., Khunti, K. & Eggo, R. M. Urgent actions and policies needed to address COVID-19 among UK ethnic minorities. *Lancet* **396**, 1866–1868 (2020).

245. Khan, S., Asif, A. & Jaffery, A. E. Language in a time of COVID-19: literacy bias ethnic minorities face during COVID-19 from online information in the UK. *J. Racial Ethn. Heal. Disparities* 1–7 (2020). doi:10.1007/s40615-020-00883-8

246. Topriceanu, C.-C. *et al.* Inequality in access to health and care services during lockdown – Findings from the COVID-19 survey in five UK national longitudinal studies. *BMJ Open* **11**, e045813 (2020).

247. Eshareturi, C., Wareham, A., Rattray, M., Haith-Cooper, M. & McCarthy, R. An exploration of the impact of SARS-CoV-2 (COVID-19) restrictions on marginalised groups in the UK. *Public Health* **197**, 6–10 (2021).

248. Le, K. & Nguyen, M. The psychological consequences of COVID-19 lockdowns. *Int. Rev. Appl. Econ.* **35**, 147–163 (2021).

249. Hamman, M. K. Disparities in COVID-19 mortality by county racial composition and the role of spring social distancing measures. *Econ. Hum. Biol.* **41**, 100953 (2021).

250. Holloway, I. W. *et al.* Associations Between Physical Distancing and Mental Health, Sexual Health and Technology Use Among Gay, Bisexual and Other Men Who Have Sex With Men During the COVID-19 Pandemic. *J. Homosex.* **68**, 692–708 (2021).

251. Salerno, J. P., Devadas, J., Pease, M., Nketia, B. & Fish, J. N. Sexual and gender minority stress amid the COVID-19 pandemic: implications for LGBTQ young persons' mental health and well-being. *Public Health Rep.* **135**, 721–727 (2020).

252. Priyadarshini, S. & Swain, S. C. Impact of covid-19 on the socioeconomic life and psychology of transgenders: Insights from india. *Int. J. Res. Pharm. Sci.* **11**, 126–132 (2020).

253. Jones, B. A., Bowe, M., McNamara, N., Guerin, E. & Carter, T. Exploring the mental health experiences of young trans and gender diverse people during the Covid-19 pandemic. *Int. J. Transgender Heal.* 1–13 (2021). doi:10.1080/26895269.2021.1890301

254. Stephenson, R. *et al.* Sex in the time of COVID-19: results of an online survey of gay, bisexual and other men who have sex with men's experience of sex and HIV prevention during the US COVID-19 epidemic. *AIDS Behav.* **25**, 40–48 (2020).

255. Melin, K., Santiago Quiñones, D. & Rodríguez-Díaz, C. E. Socially distant and out of reach: Unintended consequences of COVID-19 prevention efforts on transgender and gender non-binary populations in Puerto Rico. *J. Subst. Abuse Treat.* **122**, (2021).

256. Rhodes, S. D. *et al.* A rapid qualitative assessment of the impact of the COVID-19 pandemic on a racially/ethnically diverse sample of gay, bisexual, and other men who have sex with men living with HIV in the US South. *AIDS Behav.* **25**, 58–67 (2021).

257. Poteat, T. C., Reisner, S. L., Miller, M. & Wirtz, A. L. Vulnerability to COVID-19-related harms among transgender women with and without HIV infection in the Eastern and Southern U.S. *J. Acquir. Immune Defic. Syndr.* **85**, e67–e69 (2020).

258. Nelson, K. M., Gordon, A. R., John, S. A., Stout, C. D. & Macapagal, K. "Physical Sex Is Over for Now": impact of COVID-19 on the well-being and sexual health of adolescent sexual minority males in the U.S. *J. Adolesc. Heal.* **67**, 756–762 (2020).

259. Torres, T. S. *et al.* Impact of COVID-19 pandemic on sexual minority populations in Brazil: An analysis of social/racial disparities in maintaining social distancing and a description of sexual behavior. *AIDS Behav.* **25**, 73–84 (2021).

260. Michie, S. *et al.* Reducing SARS-CoV-2 transmission in the UK: A behavioural science approach to identifying options for increasing adherence to social distancing and shielding vulnerable people. *Br. J. Health Psychol.* **25**, 945–956 (2020).

261. Kim, J. Y., Han, J.-O. & Lee, H. Recommendation for response to the COVID-19 pandemic: Korean context of "distancing in daily life," considering vulnerable population. *Int. J. Equity Health* **19**, 1–5 (2020).

262. Simon, J., Helter, T. M., White, R. G., Boor, C. van der & Łaszewska, A. Impacts of the Covid-19 lockdown and relevant vulnerabilities on capability well-being, mental health and social support: an Austrian survey study. *BMC Public Health* **21**, 1–12 (2021).

263. Simon, J., Helter, T. M., White, R. G., van der Boor, C. & Łaszewska, A. Capability impacts of the Covid-19 lockdown in association with mental well-being, social connections and existing vulnerabilities: An Austrian survey study. *medRxiv* (2020). doi:10.1101/2020.11.14.20231142

264. de Vries, D., Pols, J., M'charek, A. & van Weert, J. The impact of physical distancing on socially vulnerable people needing care during the COVID-19 pandemic in the Netherlands. *Int. J. Care Caring* **6**, 123–140 (2022).

265. Yang, X. *et al.* Social, cognitive, and eHealth Mechanisms of COVID-19-related lockdown and mandatory quarantine that potentially affect the mental health of pregnant women in China: Cross-sectional survey study. *J. Med. Internet Res.* **23**, 1–17 (2021).

266. Djulbegovic, B., Weiss, D. J. & Hozo, I. Evaluation of the U.S. governors' decision when to issue stay-at-home orders. *J. Eval. Clin. Pract.* **26**, 1347–1351 (2020).

267. Coppola, I. & Rania, N. Psychological impact of the lockdown in Italy due to the COVID-19 outbreak: Are there gender differences? *Front Psychol* **12**, 1–11 (2021).

268. WHO. *Overview of public health and social measures in the context of COVID-19 (Interim guidance). World Health Organization 2020* (2020).

269. Yale University. Categories of vulnerability – specific vulnerable populations. *HSP Module 7: Protecting Vulnerable Subjects* Available at: https://assessment-module.yale.edu/human-subjects-protection/categories-vulnerability-specific-vulnerable-populations. (Accessed: 27th July 2021)

270. NBAC (National Bioethics Advisory Commission of the United States). *Ethical and policy issues in research involving human participants*. *Report and Recommendations of the National Bioethics Advisory Commission* (2001).

271. WHO. COVID-19: vulnerable and high risk groups. (2021). Available at: https://www.who.int/westernpacific/emergencies/Covid-19/information/high-risk-groups. (Accessed: 20th March 2022)

272. Public Health England. Guidance on social distancing for everyone in the UK. *Gov.UK* (2020). Available at: https://www.gov.uk/government/publications/covid-19-guidance-on-social-distancing-and-for-vulnerable-people/guidance-on-social-distancing-for-everyone-in-the-uk-and-protecting-older-people-and-vulnerable-adults. (Accessed: 3rd August 2021)

273. Schneider, A. L. & Ingram, H. M. Social construction of target populations: Implications for politics and policy. *Am. Polit. Sci. Rev.* **87**, 334–347 (1993).

274. Vaughan, E. & Tinker, T. Effective health risk communication about pandemic influenza for vulnerable populations. *Am. J. Public Health* **99**, 324–332 (2009).

275. Mikolai, J., Keenan, K. & Kulu, H. Intersecting household-level health and socio-economic vulnerabilities and the COVID-19 crisis: An analysis from the UK. *SSM - Popul. Heal.* **12**, 100628 (2020).

276. Mishra, S. V, Gayen, A. & Haque, S. M. COVID-19 and urban vulnerability in India. *Habitat Int.* **103**, (2020).

277. Mikolai, J., Keenan, K. & Kulu, H. Intersecting household-level health and socio-economic vulnerabilities and the COVID-19 crisis: An analysis from the UK. *SSM - Popul. Heal.* **12**, (2020).

278. Team, V. & Manderson, L. How COVID-19 Reveals Structures of Vulnerability. *Med. Anthropol. Cross Cult. Stud. Heal. Illn.* **39**, 671–674 (2020).

279. OECD. *COVID-19: Protecting people and societies*. *OECD Policy Responses to Coronavirus (COVID-19)* (OECD, 2020). doi:10.1787/9870c393-en

280. Bioethics Advisory Committee of Singapore. *The future of bioethics in Singapore*. **June**, (2021).

281. Bank, W. Social sustainability and inclusion - COVID-19 (Coronavirus) response [WWW Document]. (2021).

282. Skinner, D. *et al.* Towards a definition of orphaned and vulnerable children. *AIDS Behav.* **10**, 619–626 (2006).

283. Skinner, D. *Defining orphaned and vulnerable children*. (HSRC press, 2004).

284. World Bank. COVID-19 to add as many as 150 million extreme poor by 2021. *The World Bank: Press Release* (2020). Available at: https://www.worldbank.org/en/news/press-release/2020/10/07/covid-19-to-add-as-many-as-150-million-extreme-poor-by-2021. (Accessed: 17th September 2021)

285. World Bank. *Poverty and shared prosperity 2020: Reversals of fortune*. *Lecture Notes in Computer Science (including subseries Lecture Notes in Artificial Intelligence and Lecture Notes in Bioinformatics)* **3678 LNBI**, (World Bank, 2020).

286. United Nations (UN). Impacts of COVID-19 disproportionately affect poor and vulnerable: UN chief. *UN News: Global perspective Human stories* (2020). Available at: https://news.un.org/en/story/2020/06/1067502. (Accessed: 17th September 2021)

287. Lakner, C., Yonzan, N., Mahler, D. G., Aguilar, R. A. C. & Wu, H. Updated estimates of the impact of COVID-19 on global poverty: Looking back at 2020 and the outlook for 2021. *World Bank Blogs* (2021). Available at: https://blogs.worldbank.org/opendata/updated-estimates-impact-covid-19-global-poverty-looking-back-2020-and-outlook-2021?cid=pov_tt_poverty_en_ext. (Accessed: 17th September 2021)

288. World Bank. Understanding poverty. (2021). Available at: https://www.worldbank.org/en/topic/poverty. (Accessed: 17th September 2021)

289. Rogers, A. C. Vulnerability, health and health care. *J. Adv. Nurs.* **26**, 65–72 (1997).

290. United Nations. International Convention on the Protection of the Rights of All Migrant Workers and Members of Their Families (adopted 18 December 1990, entered into force 1 July 2003). *United Nations Treaty Ser. (Doc. A/RES/45/158)* **2220**, (2003).

291. United Nations. Researching the impact of the pandemic on internal migrant workers in India. *United Nations: Academic Impact* (2021). Available at: https://www.un.org/en/academic-impact/researching-impact-pandemic-internal-migrant-workers-india. (Accessed: 17th September 2021)

292. International Labour Organization (ILO). Migrant domestic workers. (2021). Available at: https://www.ilo.org/global/topics/labour-migration/policy-areas/migrant-domestic-workers/lang--en/index.htm. (Accessed: 31st August 2021)

293. Simpson, P. L. & Butler, T. G. Covid-19, prison crowding, and release policies. *BMJ* **369**, 1–2 (2020).

294. Simpson, P. L., Simpson, M., Adily, A., Grant, L. & Butler, T. Prison cell spatial density and infectious and communicable diseases: A systematic review. *BMJ Open* **9**, e026806 (2019).

295. Liebling, A. Vulnerability and prison suicide. *Br. J. Criminol.* **35**, 173–187 (1995).

296. Morash, M., Jeong, S., Bohmert, M. N. & Bush, D. R. Men's vulnerability to prisoner-on-prisoner sexual violence: A state correctional system case study. *Prison J.* **92**, 290–311 (2012).

297. Henry, B. F. Social distancing and incarceration: policy and management strategies to reduce COVID-19 transmission and promote health equity through decarceration. *Heal. Educ. Behav.* **47**, 536–539 (2020).

298. Lindström, M., Ahlstrand, E. & Kärrholm, J. Sweden's response to the COVID-19 outbreak. *Vict. Offenders* **15**, 1203–1214 (2020).

299. Centers for Disease Control and Prevention (USA). Disability and health overview. (2020). Available at: https://www.cdc.gov/ncbddd/disabilityandhealth/disability.html. (Accessed: 31st August 2021)

300. World Health Organization (WHO). Disability. Available at: https://www.who.int/health-topics/disability#tab=tab_1. (Accessed: 17th September 2021)

301. Batavia, A. I. & Beaulaurier, R. L. The financial vulnerability of people with disabilities: Assessing poverty risks. *J. Sociol. Soc. Welf.* **28**, 139–162 (2001).

302. Pyett, P. M. & Warr, D. J. Vulnerability on the streets: Female sex workers and HIV risk. *AIDS Care - Psychol. Socio-Medical Asp. AIDS/HIV* **9**, 539–547 (1997).

303. Rekart, M. L. Caring for sex workers. *BMJ* **351**, 26–30 (2015).

304. Ranebennur, V., Gaikwad, S., Ramesh, S. & Bhende, A. Addressing vulnerabilities of female sex workers in an HIV prevention intervention in Mumbai and Thane: Experiences from the Aastha project. *HIV/AIDS - Res. Palliat. Care* **6**, 9–18 (2014).

305. Calderbank, R. Abuse and disabled people: Vulnerability or social indifference? *Disabil. Soc.* **15**, 521–534 (2000).

306. Hague, G., Thiara, R. & Mullender, A. Disabled women, domestic violence and social care: The risk of isolation, vulnerability and neglect. *Br. J. Soc. Work* **41**, 148–165 (2011).

307. The UN Refugee Agency (UNHCR). 'Refugees' and 'Migrants' – Frequently Asked Questions (FAQs). (2016). Available at: https://www.unhcr.org/en-us/news/latest/2016/3/56e95c676/refugees-migrants-frequently-asked-questions-faqs.html. (Accessed: 17th September 2021)

308. Black, R. Livelihoods under stress: A case study of refugee vulnerability in Greece. *J. Refug. Stud.* **7**, 360–377 (1994).

309. Johnson, R. A. I. Refugee camp security: Decreasing vulnerability through demographic controls. *J. Refug. Stud.* **24**, 23–46 (2011).

310. Dhesi, S., Isakjee, A. & Davies, T. Public health in the Calais refugee camp: Environment, health and exclusion. *Crit. Public Health* **28**, 140–152 (2018).

311. Hameed, S., Sadiq, A. & Din, A. U. The increased vulnerability of refugee population to mental health disorders. *Kansas J. Med.* **11**, 20–23 (2019).

312. Pinzón-Espinosa, J., Valdés-Florido, M. J., Riboldi, I., Baysak, E. & Vieta, E. The Covid-19 pandemic and mental health of refugees, asylum seekers, and migrants. *J. Affect. Disord.* **280**, 407–408 (2021).

313. United Nations. Indigenous peoples and ethnic minorities: Marginalization is the norm. in

Promoting inclusion through social protection: The report on the world social situation 2018 97–108 (United Nations, 2018). doi:10.18356/14642ccc-en

314. GOV.UK. Writing about ethnicity. Available at: https://www.ethnicity-facts-figures.service.gov.uk/style-guide/writing-about-ethnicity. (Accessed: 17th September 2021)

315. Parent, M. C., DeBlaere, C. & Moradi, B. Approaches to research on intersectionality: Perspectives on gender, LGBT, and racial/ethnic identities. *Sex Roles* **68**, 639–645 (2013).

316. Littlejohn, T., Poteat, T. & Beyrer, C. Sexual and gender minorities, public health, and ethics. in *The Oxford Handbook of Public Health Ethics* (eds. Mastroianni, A. C., Kahn, J. P. & Kass, N. E.) 231–244 (Oxford University Press, 2019). doi:10.1093/oxfordhb/9780190245191.013.17

317. Ganju, D. & Saggurti, N. Stigma, violence and HIV vulnerability among transgender persons in sex work in Maharashtra, India. *Cult. Heal. Sex.* **19**, 903–917 (2017).

318. Melin, K., Santiago Quiñones, D. & Rodríguez-Díaz, C. E. Socially distant and out of reach: Unintended consequences of COVID-19 prevention efforts on transgender and gender non-binary populations in Puerto Rico. *J. Subst. Abuse Treat.* **122**, (2021).

319. Nelson, K. M., Gordon, A. R., John, S. A., Stout, C. D. & Macapagal, K. "Physical Sex Is Over for Now": impact of COVID-19 on the well-being and sexual health of adolescent sexual minority males in the U.S. *J. Adolesc. Heal.* **67**, 756–762 (2020).
