# Supplementary material for: A scoping review of the impacts of COVID-19 physical distancing measures on vulnerable population groups
Source: Nat Commun. 2023 Feb 3;14:599. doi: 10.1038/s41467-023-36267-9 (PMC9897623; doi:10.1038/s41467-023-36267-9)
Supplement: Supplementary file 3 — Reporting Summary [file 41467_2023_36267_MOESM3_ESM.pdf]

nature portfolio

Corresponding author(s): Araz Taeihagh

Last updated by author(s): Jan 10, 2023

Reporting Summary

Nature Portfolio wishes to improve the reproducibility of the work that we publish. This form provides structure for consistency and transparency in reporting. For further information on Nature Portfolio policies, see our Editorial Policies and the Editorial Policy Checklist.

Statistics

For all statistical analyses, confirm that the following items are present in the figure legend, table legend, main text, or Methods section.

n/a	Confirmed	
☒	☐	The exact sample size (*n*) for each experimental group/condition, given as a discrete number and unit of measurement
☒	☐	A statement on whether measurements were taken from distinct samples or whether the same sample was measured repeatedly
☒	☐	The statistical test(s) used AND whether they are one- or two-sided<br>*Only common tests should be described solely by name; describe more complex techniques in the Methods section.*
☒	☐	A description of all covariates tested
☒	☐	A description of any assumptions or corrections, such as tests of normality and adjustment for multiple comparisons
☒	☐	A full description of the statistical parameters including central tendency (e.g. means) or other basic estimates (e.g. regression coefficient) AND variation (e.g. standard deviation) or associated estimates of uncertainty (e.g. confidence intervals)
☒	☐	For null hypothesis testing, the test statistic (e.g. *F*, *t*, *r*) with confidence intervals, effect sizes, degrees of freedom and *P* value noted<br>*Give P values as exact values whenever suitable.*
☒	☐	For Bayesian analysis, information on the choice of priors and Markov chain Monte Carlo settings
☒	☐	For hierarchical and complex designs, identification of the appropriate level for tests and full reporting of outcomes
☒	☐	Estimates of effect sizes (e.g. Cohen's *d*, Pearson's *r*), indicating how they were calculated

Our web collection on statistics for biologists contains articles on many of the points above.

Software and code

Policy information about availability of computer code

Data collection	No software/tool/algorithm was used for data collection
Data analysis	Excel 16 (Microsoft Corporation)

For manuscripts utilizing custom algorithms or software that are central to the research but not yet described in published literature, software must be made available to editors and reviewers. We strongly encourage code deposition in a community repository (e.g. GitHub). See the Nature Portfolio guidelines for submitting code & software for further information.

Data

Policy information about availability of data

All manuscripts must include a data availability statement. This statement should provide the following information, where applicable:

- Accession codes, unique identifiers, or web links for publicly available datasets
- A description of any restrictions on data availability
- For clinical datasets or third party data, please ensure that the statement adheres to our policy

The authors declare that all summary data generated during this research were included in the manuscript. All the articles included in this scoping review (reported in Supplementary Table 1 and Table 2), were identified from a comprehensive search of ten electronic databases typically used in systematic/scoping review research (PubMed, Scopus, Web of Science, ProQuest, ProQuest Coronavirus Research Database, Embase, ERIC, LITCOVID, Cochrane database of systematic reviews and WHO's database of COVID-19 literature), and their full texts are accessible through open access or institutional or personal journal subscriptions.

Human research participants

Policy information about studies involving human research participants and Sex and Gender in Research.

Reporting on sex and gender	Not applicable. This is a scoping review, and no primary data collection was conducted. We reviewed the impacts of COVID-19 physical distancing measures on vulnerable populations, including people from sexual and gender minorities.
Population characteristics	Not applicable. This is a scoping review, and no primary data collection was conducted.
Recruitment	Not applicable. This is a scoping review, and no primary data collection was conducted.
Ethics oversight	Not applicable. This is a scoping review, and no primary data collection was conducted.

Note that full information on the approval of the study protocol must also be provided in the manuscript.

Field-specific reporting

Please select the one below that is the best fit for your research. If you are not sure, read the appropriate sections before making your selection.

☐ Life sciences ☒ Behavioural & social sciences ☐ Ecological, evolutionary & environmental sciences

For a reference copy of the document with all sections, see nature.com/documents/nr-reporting-summary-flat.pdf

Behavioural & social sciences study design

All studies must disclose on these points even when the disclosure is negative.

Study description	This is a scoping review that examines the impacts of COVID-19 physical distancing measures on vulnerable populations in the society. A systematic search of ten databases was conducted to identify relevant studies, including PubMed, Scopus, Web of Science, ProQuest, ProQuest Coronavirus Research Database, Embase, ERIC (Educational Resource Information Center) database, LITCOVID, Cochrane database of systematic reviews and WHO's database of COVID-19 literature. The studies included in this review consist of qualitative studies, quantitative studies, and mixed-methods studies.
Research sample	This is a scoping review, and we searched ten electronic databases typically used in systematic/scoping review research. No primary data collection was conducted.
Sampling strategy	This is a scoping review, and we searched ten electronic databases typically used in systematic/scoping review research. No primary data collection was conducted.
Data collection	We searched ten databases to identify articles relevant to the impacts of COVID-19 physical distancing measures on vulnerable population groups, including PubMed, Scopus, Web of Science, ProQuest, ProQuest Coronavirus Research Database, Embase, ERIC (Educational Resource Information Center) database, LITCOVID, Cochrane database of systematic reviews, and WHO's database of COVID-19 Global literature on coronavirus disease. We followed the below inclusion criteria to identify relevant studies.<br>Inclusion criteria:<br>1) Studies that examined vulnerable populations as populations of interests;<br>2) Studies that examined various physical distancing measures from the public policy and/or legal perspectives;<br>3) Peer-reviewed studies (empirical, conceptual and review studies), policy briefs, reports, editorials, commentaries, perspectives, and letters were included;<br>4) Studies employing jurisdictions (prefecture/district/city/county, state/province, single country, multi-country) as a unit of analysis;<br>5) Studies employing quantitative, qualitative or mixed methods as research designs;<br>6) Studies published as full-text articles in English language between November 2019 till June 2022.
Timing	This is a scoping review, and we searched ten electronic databases typically used in systematic/scoping review research from March 2021 to April 2021, and updated the search in June 2022. We collected relevant studies published between November 2019 till June 2022. No primary data collection was conducted.
Data exclusions	Exclusion criteria:<br>1) Studies that examined physical distancing measures but did not mention their impacts on vulnerable populations;<br>2) Clinical studies on the COVID-19 pandemic without public policy and/or law dimensions;<br>3) Studies published before November 2019;<br>4) Full-text articles not accessible or studies published in non-English languages
Non-participation	This is a scoping review, and no primary data collection was conducted.
Randomization	This is a scoping review, and no primary data collection was conducted.

Reporting for specific materials, systems and methods

We require information from authors about some types of materials, experimental systems and methods used in many studies. Here, indicate whether each material, system or method listed is relevant to your study. If you are not sure if a list item applies to your research, read the appropriate section before selecting a response.

Materials & experimental systems

n/a	Involved in the study
☒	☐ Antibodies
☒	☐ Eukaryotic cell lines
☒	☐ Palaeontology and archaeology
☒	☐ Animals and other organisms
☒	☐ Clinical data
☒	☐ Dual use research of concern

Methods

n/a	Involved in the study
☒	☐ ChIP-seq
☒	☐ Flow cytometry
☒	☐ MRI-based neuroimaging
